# Proceedings of International Conference on Cell Death in Cancer and Toxicology

**DOI:** 10.1002/cam4.1413

**Published:** 2018-02-19

## Signaling by apoptotic cells in development and cancer

**A. Perez‐Garijo & H. Steller**

*The Rockefeller University, New York, NY, USA*

*Email:* Hermann.Steller@rockefeller.edu

Apoptotic cells can produce signals to instruct cells in their local environment, including factors that stimulate proliferation. This apoptosis‐induced proliferation has been shown to play important roles in tissue regeneration, tumor growth and relapse. We recently identified a novel mode of communication by which apoptotic cells induce additional apoptosis in the same tissue. To investigate the role of apoptosis‐induced apoptosis during development we studied the coordinated cell death that occurs in the adult wing of *Drosophila*. We observe a non‐autonomous effect upon inhibition of apoptosis in the wing, suggesting there is a communication between dying cells to achieve synchronized cell death. Our results indicate there might be a wave of apoptosis traveling from the posterior compartment towards the anterior. Surprisingly, TNF and JNK pathways are not involved in this propagation of apoptosis, suggesting a different mechanism for apoptotic signaling in developmental and stress situations.

## Targeting phosphatidylserine/TAM receptor/PD‐L1 axis as vulnerability in cancer

**R. B. Birge, C. Kasikara, K. Geng, D. Calianese & V. Davra**

*Rutgers, Biomedical and Health Sciences, Department of Microbiology, Biochemistry and Molecular Genetics, New Jersey Medical School, Newark, NJ, USA*

*Email:* birgera@njms.rutgers.edu

The physiological fate of cells that die by apoptosis is their rapid recognition and clearance by both non‐professional and professional phagocytes (efferocytosis). Apoptotic cells, by externalizing phosphatidylserine (PS) from the internal to the external surface of the plasma membrane, act as potent negative regulators of immune responses, promoting immune‐suppression and the resolution of inflammation under physiological conditions to maintain tissue tolerance. In contrast to the homeostatic function of efferocytosis under physiological conditions, tumors, in particular, exist in a chronic dynamic balance of proliferation, metabolic stress, and apoptosis that magnify the post‐mortem immunosuppressive effects of apoptotic cells in the tumor microenvironment (TME). In turn, persistent apoptotic cells in the TME suppress host tumor immunity by engaging a series of PS receptors called TAM receptors (Tyro3, Axl, and Mertk). While TAMs are overexpressed on a vast array of tumor types, whereby the level of expression correlates with the tumor grade and the emergence of chemo and radio resistance to targeted therapeutics, they are also implicated as inhibitory receptors expressed on infiltrating myeloid‐derived cells, where they act as so‐called myeloid checkpoint inhibitors. We have recently shown that TAMs can act as PS‐sensing receptors not only to induce PS‐mediated efferocytosis but also to up‐regulate the immune checkpoint inhibitory ligand PD‐L1 demonstrating the existing of a PS/PS‐R (TAM‐receptor)/PD‐L1 axis that operates in the TME to drive immune escape. Finally, we will discuss the current molecular rationale that anti‐PS, anti‐TAM, and anti‐PD‐L1 based therapeutics may have therapeutic value as combinatorial checkpoint inhibitors in cancer immunotherapy.

## The many faces of mitochondria

**A. Gross**

*Department of Biological Regulation, Weizmann Institute, Rehovot, Israel*

*Email:* atan.gross@weizmann.ac.il

Mitochondria are highly dynamic organelles that play fundamental roles in pivotal cellular processes including energy production/metabolism, calcium homeostasis, and apoptosis. In our lab we are specifically interested in understanding how these different mitochondrial processes are regulated/coordinated to determine the fate of our cells. Many of our studies are focused on a novel mitochondrial protein named MTCH2 that acts as a receptor for the pro‐apoptotic BID protein. Interestingly, conditional knockout of MTCH2 in several different mouse tissues results in significant alterations to mitochondria function and structure leading to changes in cell fate and disease outcome. A better understanding of MTCH2's mechanism of action will likely uncover hidden connections between the many functions of mitochondria.

## The key role for a superoxide driven pro‐oxidant state in tumour cells chemo‐resistance and metastatic potential

**M. V. Clement**

*Department of Biochemistry, Yong Loo Lin School of Medicine, National University of Singapore, Singapore*

*Email:* bchmvc@nus.edu.sg

Whereas earlier reports highlighted a tumor suppressor role for the mitochondrial superoxide, MnSOD, many reports show an increase in the enzyme expression in a variety of human tumors including aggressive breast carcinoma. To that regards, we have provided evidence to link increased MnSOD expression with the aggressive basal‐like breast cancer subtype, and underscored the judicious use of PPAR*γ* ligands for specifically downregulating MnSOD to overcome the chemo‐resistance in this subtype of breast cancer. In addition, analysis of breast carcinomas transcriptome from The Cancer Genome Atlas (TCGA) database revealed strong positive correlation between tumours’ metastatic potential and the expression of MnSOD. The positive correlation between MnSOD and metastatic potential was significant and consistent across all breast cancer subtypes underscoring the involvement of MnSOD in the regulation of the switch between the epithelial and the mesenchymal‐associated phenotype. Interestingly, the mechanism involved in the regulation of tumor cells chemo‐resistance and switch from an epithelial to a mesenchymal phenotype by MnSOD is linked tumorigenic superoxide driven pro‐oxidant state.

## Caspase‐independent developmental cell death triggered by the lysosomal nuclease (DNaseII)‐induced DNA damage in *Drosophila*

**L. Tarayrah & E. Arama**

*Department of Molecular Genetics, Weizmann institute of Science, Rehovot, Israel*

*Email:* eli.arama@weizmann.ac.il

Programmed cell death (PCD) is an intracellular genetic program that can essentially be activated by all metazoan cells. Apoptosis is the most abundant form of PCD and is manifested by the activation of cysteine proteases called caspases. However, activation of caspases does not always lead to apoptosis, whereas cell death is sometimes executed by caspase‐independent alternative cell death (ACD) pathways. Considering that cancer cells have developed ways to evade apoptotic cell death, uncovering the molecular mechanisms underlying ACD pathways can be of tremendous therapeutic value. However, progress in this field has been considerably slow, primarily due to the very few physiological paradigms of ACD. In this study, we characterize the non‐apoptotic developmental cell death of primordial germ cells (PGCs) in the *Drosophila* embryo. During the early stages of embryo development, germ‐line progenitors, known as pole cells, originate at the posterior pole and must migrate to their somatic cell partners in order to form the gonads. Prior studies have demonstrated the involvement of guidance cues which drive PGC migration, while mismigrating germ cells that remain ectopic to the gonad are eliminated. However, little is known about the underlying pathways and mechanisms governing this elimination mechanism. Our results indicate that PGCs that do not coalesce into a gonad by stage 13 of embryo development undergo caspase‐independent cell death that involves the activity of lysosomes. We find that PGC death is triggered cell‐autonomously by the lysosomal (acidic) nuclease DNaseII and the co‐activator Apoptosis Inducing Factor (AIF). Our results support a model in which PGC death is primarily controlled by the DNA damage response pathway, wherein manipulating the DNA damage checkpoint and/or response pathways attenuate PGC death. These findings reveal a developmental ACD pathway, and provide an excellent physiological system to investigate caspase‐independent cell death mechanisms *in vivo*.

## Toxicity from vesicating agents and cell death mechanisms

**N. Tewari‐Singh**

*Department of Pharmaceutical Sciences, Skaggs School of Pharmacy and Pharmaceutical Sciences, University of Colorado Anschutz Medical Campus, Aurora, CO, USA*

*Email:* Neera.Tewari‐Singh@ucdenver.edu

Chemical threat agents (e.g. vesicating agents, nerve agents, choking agents, lachrymators and industrial chemicals), can be accidently released or intentionally used in warfare or terrorist activity due to their low cost of manufacturing, easy synthesis and devastating multi‐organ toxic effects. Vesicating agents like sulfur mustard (mustard gas; SM) have been most extensively used in various conflicts since World War I; these lead to the formation of vesicles/blisters apart from their ability to cause acute and debilitating injuries to multiple organs. Vesicating agents include: mustard agents such as SM [bis(2‐chloroethyl)sulfide] and nitrogen mustard [HN1 (bis(2‐chloroethyl) ethylamine; arsenical vesicants such as lewisite [L; dichloro(2‐chlorovinyl) arsine]; and nettle agent phosgene oxime (CX; dichloroformoxime). Vesicating agents are cytotoxic, mutagenic and possible carcinogenic agents that cause direct damage to the skin, ocular and respiratory tissues. Upon exposure, vesicating agents cause severe injury to the most exposed skin tissue that is associated with erythema, inflammation, blister formation/urticaria and cell death mainly of basal epidermal cells leading to painful skin lesions.

Although the mechanism of injury from vesicating agents is complex and not fully defined, their cytotoxicity is mainly due to their alkylating and/or thiol depleting properties. SM reacts with cellular molecules, mainly nucleic acids forming cross‐links and mono‐adducts with DNA causing DNA damage that could be a direct event or via formation of reactive oxygen and nitrogen species. Studies have indicated that DNA damage is one of the key events after vesicant exposure leading to H2A.X and p53 phosphorylation, Poly (ADP‐ribose) polymerase (PARP) activation, NAD+/ATP depletion, cell cycle arrest, and activation of DNA damage repair or apoptotic/necrotic cell death. In addition, vesicating agent‐induced cytotoxicity is associated with infiltration of macrophages, mast cells and neutrophils, enhanced production of inflammatory cytokines and chemokines, and increased oxidative stress. Our studies to elucidate mechanisms of cytotoxicity from exposure to vesicating agents in relevant skin injury models is anticipated to lead to the identification novel mechanism‐based therapies against SM and CX‐induced toxic effects from skin exposure, with the potential to be developed as effective countermeasures in a mass casualty event.

## Mitochondrial apoptosis is regulated by OXPHOS complexes, interleukins, and unfolded protein response in cancer

**S. Kumar, A. Chaudhary, R. Kumar, N. Yadav & D. Chandra**

*Department of Pharmacology and Therapeutics, Roswell Park Cancer Institute, Buffalo, NY, USA*

*Email:* Dhyan.Chandra@roswellpark.org

Apoptotic cell death machinery is defective in cancer leading to the development of resistance to current therapy. Thus cancer cells develop resistance to multiple types of anticancer agents, however, whether they adopt similar or differential mechanisms to evade cell death in response to a broad spectrum of cancer therapeutics is not fully defined. We recently demonstrated that DNA‐damaging agents (etoposide and doxorubicin), endoplasmic reticulum stressor (thapsigargin), and histone deacetylase inhibitor (apicidin) target oxidative phosphorylation (OXPHOS) for apoptosis induction, whereas other anticancer agents including staurosporine, taxol, and sorafenib induced apoptosis in an OXPHOS‐independent manner. Thapsigargin‐induced caspase activation was reduced upon abrogation of complex‐I or gross‐complexes, whereas a reverse trend was observed with apicidin. Cytokines and inflammation regulate mitochondrial complexes, cell survival, and cellular apoptosis. Our findings suggest that apicidin and thapsigargin target different OXPHOS complexes, we further investigated the effect of thapsigargin or apicidin on functional significance of cytokines in mitochondrial function and apoptotic cell death. We first screened modulation of various cytokines in response to anticancer agents and observed that interleukin‐8 (IL‐8) was most prominently released during stress. Then we further investigated the role of interleukin‐8 (IL‐8)in cell survival and apoptotic cell death. Altered release of cytokines including TGF‐*β*, IL‐8, IFN‐*γ* and TNF‐*α* were observed in the medium of colon and prostate cancer cells treated with anticancer drugs including apicidin and thapsigargin. Dose dependent up‐regulation of IL‐8 was observed upon apicidin and thapsigargin treatment, which was corroborated with cytochrome‐c release from mitochondrial compartment. Significantly elevated caspase‐3 was observed upon combined exposure of apicidin and thapsigargin with altered IL‐8 expression and release. Interestingly, knock down of *caspase‐8* and *caspase‐9* down‐regulated IL‐8 expression, suggesting the involvement of caspases on regulation of IL‐8‐mediated cellular and mitochondrial function. *TRADD* knock down attenuated apoptosis induction in response to these anticancer agents. These findings suggest that mitochondrial dysfunction and functional TRADD are required for IL‐8 mediated cancer cell survival. Further mechanistic analysis demonstrated that unfolded protein response plays a critical role in the regulation of IL‐8‐induced cellular proliferation and death. Using tumor xenograft mouse model, we further defined the role of unfolded protein response as well as IL‐8 in tumorigenesis and tumor regression. Together, we dissect the novel role of IL‐8, OXPHOS complexes, and unfolded protein response in regulating cancer cell survival and death, which may have significance in understanding tumorigenesis, drug resistance, and tumor recurrence.

## Role of crosstalk between estrogen receptors and tumor suppressor p53 signaling in therapeutic resistance of breast cancer

**G. M. Das^1^, S. Kulkarni^1,2,3^, C. Oturkar^1^, S. B. Edge^1^, J. Wang^1^, J. H. Wilton^1^, W. M. Swetzig^1^, A. Adjei^1^, R. Bies^1^, A. D. Hutson^1^, A. Groman^1^, C. D. Morrison^1^, J. Fetterly^1^, S. Kumar^1^ & H. H. Cappuccino^1^**

*^1^Roswell Park Cancer Institute, Buffalo, NY, USA; ^2^University of Chicago, Chicago, IL, USA; ^3^Northwestern University, Chicago, IL, USA*

*Email:* gokul.das@roswellpark.org

One of the focus areas of research in our laboratory is analyzing the role of estrogen receptors and tumor suppressor protein p53 in breast cancers. Although tamoxifen (TAM) is used widely to treat estrogen receptor alpha (ER*α*)‐positive luminal breast cancer, a large percentage of patients are resistant to TAM therapy. Although about 80% of ER*α*‐positive breast cancers express wild type p53 (wt‐p53), the p53 is largely dysfunctional in these cancers. We have previously reported that ER*α* binds to wt‐p53 and inactivates its tumor suppressor activity. This interaction is augmented by 17*β*‐estradiol and disrupted by TAM. Moreover, retrospective clinical studies have shown that ER‐positive breast cancer (BC) expressing wt p53 is more responsive to TAM therapy compared to those expressing mutant p53. We conducted a window‐of‐opportunity phase 2 clinical trial in 53 women with ER‐positive BC expressing wt p53 to test our hypothesis that TAM relieves the functional suppression of p53 by ER*α*. Proximity ligation assay (PLA) demonstrated that ER*α*‐p53 interaction was disrupted in majority of TAM‐treated patients. Global transcriptome analysis in surgically resected tumors was conducted by RNA‐seq. The multiplexed libraries were sequenced on HiSeq 2500 using 100 cycle paired end sequencing and an average 50 million paired end reads were generated for each sample. TAM metabolites were measured in the plasma, tumor and surrounding normal tissue using liquid chromatography coupled with tandem mass spectrometry (LC‐MS/MS). Polymorphisms affecting TAM metabolism enzymes were determined by genotyping. Differential gene expression (DGE) analysis using DESEq2 R package followed by GSEA pathway analysis showed that 300 genes were upregulated and 272 genes were downregulated (*P* < 0.05) in response to TAM therapy. DEG included those involved in the regulation of cell cycle, apoptosis, immune response, energy metabolism, growth factors and receptors, and membrane transporters. Importantly, several p53‐target genes responded strongly to TAM therapy (repression targets of p53 were downregulated and activation targets were upregulated) while pro‐proliferative ER*α* targets were downregulated. We show that in human luminal BC, in addition to its conventional effects, TAM disrupts the ER*α*‐p53 interaction leading to functional reactivation of p53. Our data 1) supports the role of ER*α*‐p53 crosstalk in endocrine resistance of ER*α*‐positive tumors, 2) provides insight into the mechanism underlying favorable response of tumors expressing wt p53 to TAM therapy, and 3) has implications toward stratifying ER*α*‐positive BC patients to those who will or will not be responsive to TAM therapy.

## Exosomic microRNAs and chemotherapy resistance within the neuroblastoma tumor microenvironment

**K. Challagundla, P. Nallasamy & S. Chava**

*Department of Biochemistry and Molecular Biology and the Fred and Pamela Buffett Cancer Center, University of Nebraska Medical Center, Omaha, NE, USA*

*Email:* kishore.challagundla@unmc.edu

Even with aggressive treatments, high‐risk neuroblastoma (NBL) remains one of the most difficult pediatric cancers to treat. MicroRNAs (miRNAs) are small noncoding RNAs (ncRNAs) with gene expression regulatory functions and are dysregulated in almost all human tumors, including Neuroblastoma. Studies have shown that extracellular microvesicles, called exosomes, transfer miRNA from one cell to another, and affect tumor microenvironment of NBL. However, how exosomic miRNAs contribute to the development of drug resistance in the context of the tumor microenvironment has not been previously described in NBL. We showed that NBL cells: (i) secrete miRNA‐21 in exosomes; (ii) up‐regulate miRNA‐21 and trans‐activate miRNA‐155‐5p (hereafter miRNA‐155) through miRNA21‐TLR8 (Toll‐like receptor 8)‐NFкB axis in co‐culture with monocytes and exosomes; (iii) up‐ regulate miRNA‐155, telomerase activity and down‐regulate negative regulator of Telomeric Repeat Binding Factor 1 (TERF1) protein after co‐culturing with monocytes; (iv) acquire resistance to cisplatin mediated cell death both *in vitro* and *in vivo*; and (v) tumors of Neuroblastoma patients express higher levels of markers (CD163) for tumor infiltrated microenvironment cells and miRNA‐155, as well as low TERF1. These data indicate a unique role of exosomic miR‐21 and miR‐155 in the cross‐talk between NBL cells and human monocytes in the resistance to chemotherapy, through a novel exosomic miR‐21/TLR8‐NF‐кB/exosomic miR‐155/TERF1 signaling pathway.

## Studying metastatic mechanisms of lung adenocarcinoma

**P. P. Shah^1^, Z. Kurlawala^2^, D. Saforo^2^, M. Doll^2^, L. Siskind^1,2^ & L. J. Beverly^1,2,3^**

*^1^James Graham Brown Cancer Center, University of Louisville, Louisville, KY, USA; ^2^Department of Pharmacology and Toxicology, University of Louisville, Louisville, KY, USA; ^3^Department of Medicine, University of Louisville, Louisville, KY, USA*

*Email:* Levi.Beverly@louisville.edu

Lung cancer is the leading cause of cancer mortality worldwide resulting in more than 1.3 million deaths each year^2^. Despite major advances in the detection, classification and treatment of lung cancers, the overall survival of lung cancer patients is still dismal, with barely 15% of lung cancer patients surviving beyond 5 years after initial diagnosis. Treatment outcomes rely heavily on the stage at which the disease was diagnosed and first treated. Unfortunately, most lung cancers are not diagnosed until they are already at an inoperable advanced stage. In fact, 40% of lung cancers are diagnosed only after distant metastasis has already taken place. Unfortunately, even early stage lung cancers that are “completely” resected by surgery and treated with adjuvant chemotherapy progress and kill the patient up to 55% of the time. Therefore, it is critical that we expand our knowledge of basic molecular mechanisms that contribute to lung cancer pathogenesis, continue to explore novel targets for the treatment of advanced lung cancer, and identify targets to block the metastatic progression of tumors that are diagnosed and treated at an early stage. Data from large, publicly available datasets indicate multiple somatic non‐synonymous recurrent mutations in Ubiquilin (UBQLN) family genes or loss of either UBQLN1 or UBQLN2 loci in over 50% of human lung adenocarcinoma patient samples. The UBQLN family consists of 5 related proteins (UBQLN1‐4 and UBQLNL) that all contain ubiquitin‐like (UBL) and ubiquitin‐associated (UBA) domains. Our work was the first to show that UBQLN proteins regulate processes involved in tumorigenesis. In fact, our work now conclusively demonstrates that Ubiquilin1 is a *bona fide* metastasis suppressors in human lung adenocarcinoma. Importantly, since the vast majority of patients that succumb to lung cancer, die from the metastatic disease and not from the primary tumor burden the combination of these data suggest that nearly 700,000 people die world‐wide every year from lung cancers that have decreased or altered UBQLN function. The exact mechanism by which loss of UBQLN proteins leads to metastatic progression remains unclear, however metastatic cancer progression requires cells to acquire new aggressive properties, such as increased migration, invasion, survival and growth at the metastatic site. We are combining the study of UBQLN proteins with more physiologically relevant models to study how lung cancers become metastatic with the hopes of identifying new treatments for metastatic disease.

## The ubiquitin E3 ligase HUWE1 regulates p53 activation by controlling the stability of MDM2 and MDMX

**W. Feng^1,2^, S. Bang^1^ & M. Kurokawa^1,2^**

*^1^Department of Biological Sciences, Kent State University, OH, USA; ^2^Department of Molecular & Systems Biology, Geisel School of Medicine at Dartmouth, NH, USA*

*Email:* mkurokaw@kent.edu

The p53 tumor suppressor protein plays an essential role in preventing the development of cancer. The ubiquitin E3 ligase MDM2 and its homolog, MDMX, are paramount in suppressing the activity of p53, and the MDM2/MDMX heterodimer inhibits the functions of p53 within a complex network of feedback/forward signals. In healthy cells, this network tightly regulates the stability of MDM2 and MDMX, which in turn controls and responds to p53 activity. In response to cellular stress, such as DNA damage, rapid ubiquitination and degradation of both MDM2 and MDMX leads to full p53 activation. Importantly, human cancers often overexpress MDM2 and MDMX, which can directly contribute to tumorigenesis in addition to perturbing the efficacy of conventional chemotherapy and radiation therapy. Despite the critical roles of MDM2 and MDMX, the mechanism that mediates their stability remains elusive. While MDM2 itself is known to ubiquitinate MDMX for proteasomal degradation, there has been much debate as to what suppresses MDM2 prior to p53 activation. We found that: (a) another E3 ligase, HUWE1 interacts with both MDM2 and MDMX proteins, and this interaction further increases with DNA damage; (b) DNA damage‐induced degradation of MDM2 and MDMX is markedly inhibited upon HUWE1 siRNA‐mediated knockdown; and (c) HUWE1 knockdown renders cells highly resistant to DNA damaging agents by stabilizing both MDM2 and MDMX. Furthermore, we have generated a tamoxifen‐inducible *Huwe1* KO mouse line. Using this mouse model, we found that *Huwe1* loss also render tissues resistant to DNA damage‐induced p53 activation *in vivo*. Taken together, our results strongly suggest that HUWE1 is the long‐sought‐after E3 ligase responsible for DNA damage‐induced degradation of both MDM2 and MDMX, an event critical for p53 activation.

## Targeting tumor microenvironment for multiple myeloma therapy

**M. Pandey**

*Department of Biomedical Sciences, Cooper Medical School of Rowan University (CMSRU), Camden, NJ, USA*

*Email:* pandey@rowan.edu

Multiple myeloma (MM) represents 1% of all malignancies, and approximately 10% of hematological malignancies. MM remains uniformly fatal owing to intrinsic or acquired drug resistance and the median survival time is 3–5 years. One of the major factors that lay the foundation for MM relapses is the intimate relationship of myeloma cells to bone marrow microenvironment. This interaction of myeloma to bone marrow not only required for growth, it activates several key signaling pathways playing important role in survival, migration, and chemo‐resistance. Therefore, targeting myeloma cells trafficking to bone marrow and its adhesion would be a novel therapeutic strategy. It has been established that stem‐like MM cells (MMSCs) play critical role in refraction and relapse. Recent studies have illustrated that Bruton's tyrosine kinase is highly expressed in MMSCs and regulates many vital signaling pathways playing critical role in cell proliferation, survival, migration, and resistance. Our laboratory focuses on targeting the trafficking of myeloma cells to bone marrow as well several signaling pathways contributing in the survival of MM. Recently we showed that targeting of BTK and CXCR4 inhibits the survival of myeloma cells and bone loss. This contribution in this regard is significant because it is expected to have broad translational importance in the treatment of MM.

## Cell death disruption, disease prognosis and resistance to experimental and clinical cancer therapy

**P. T. Daniel**

*Clinical and Molecular Oncology, University Medical Center Charité and Max Delbrück Center for Molecular Medicine, Berlin, Germany*

*Email:* pdaniel@mdc-berlin.de

Inactivation of cell death programmes is a prerequisite for malignant transformation by oncogenes. Thus, oncogene activation ultimately triggers cell death in the absence of concomittant inhibition of apoptosis programmes. To achieve cancerogenesis, cell death signaling must be disrupted through additional genetic hits. Such cell death inhibition may be achieved through disruption of key apoptosis promoting genes through mutation, deletion or epigenetic inactivation or the up‐regulation of anti‐apoptotic players. To gain insight into these mechanisms and how they determine resistance to cancer therapies, we have studied the impact of oncogenic programmes on cell death signaling through the intrinsic and the extrinsic cell death pathways. Evidence will be provided that oncogenic pathways interfere with cell death signaling, e.g. through mTOR, EGF‐R and k‐ras mediated events through targeting of the Bcl‐2/Bax and the Bak/Mcl‐1 rheostats thereby mediating ‘oncogene addiction’. This, in turn impacts on the resistance to conventional and targeted anticancer therapies. Knowledge about the genetic programming of cancer cells may therefore help to select the right drug to circumvent resistance mechanisms in clinical cancer therapy.

## ATF‐4 orchestrates light‐induced toxicity: clinical implications for photodynamic and photobiomodulation therapy

**P. R. Arany**

*Oral Biology and Biomedical Engineering, University at Buffalo, NY, USA*

*Email:* prarany@buffalo.edu

The use of biophotonics devices for biomedical applications includes selective destruction termed Photodynamic therapy (PDT) and promotion of therapeutic responses termed Photobiomodulation (PBM) Therapy. The distinction between these two applications includes the use of exogenous versus endogenous chromophores along with differences in evoked biological responses. However, both treatments rely on the role of integrated cell stress in light‐evoked cellular responses. We have outlined multiple intra‐ and extracellular molecular pathways involved in PBM therapy that mediate the beneficial biological outcomes including NF*κ*B and latent TGF‐*β*1 activation. These light‐mediated molecular pathways have been used clinically to alleviate pain and inflammation as well as direct differentiation of stem cells to promote wound healing and tissue regeneration. More recently, we have focused on the precise cell phototoxicity responses that determine the transition from biologically therapeutic (PBM) dose regimen to detrimental (PDT) responses. We observed a key role of ATF‐4 in the endoplasmic reticulum that appears to determine the phototoxic cellular responses where low doses induces low amounts of autophagy while excessive, detrimental phototoxic doses results in excessive autophagy and cell death via apoptosis. These critical roles were confirmed using loss (siRNA) and gain (overexpression) approaches. Moreover, these responses appear to be capable of being primed by pre‐treatments to other forms of cell stressors enabling changes to effective phototoxic light doses termed Preconditioning. Hence, this work is enabling the use of these cell stress pathways as precise biomarkers to develop rigorous, safe and effective clinical biophotonics treatment regimens. This presentation will focus on our prior research investigations outlining these pathways and ongoing current efforts to translate these findings into effective human clinical therapies.

## A novel steroid hormone receptor‐based drug target for endocrine cancers

**R. Kumar**

*Department of Basic Sciences, Geisinger Commonwealth School of Medicine, Scranton, PA, USA*

*E‐mail:* rkumar@som.geisinger.edu

Steroid hormone receptors (SHRs) are well‐validated drug targets and are prognostic indicators in several endocrine‐related cancers, yet treatment of these cancers with small molecule SHR modulators (SRMs) is complicated by the current inability to restrict SHR actions to specific organ/gene targets. Due to available LBD/AF2 crystal structures, rational structure‐based design of SRMs by necessity has been limited to how compounds modulate co‐regulatory protein interactions with AF2 surfaces. Clinically this phenomenon has extensively been exploited; however, most of these SRMs bind in the ligand‐binding pocket and exhibit partial agonist or mixed agonist activities that may not be desirable. Though, it has been suggested that the tissue‐specific residual activity of SHRs in the presence of SRMs may mainly be mediated via AF1, yet due mainly to the intrinsically disordered (ID) conformation of N‐terminal domain (NTD), therapeutic targeting of the AF1 has been limited. This is despite the fact that the relative functional importance of SHRs may be decided by conformational dynamics of the highly flexible NTD/AF1 coupled with allosteric regulations. Thus, binding proteins that induce coupled folding of active NTD/AF1 conformations may either function directly as a co‐regulator(CoR) or reorganize structure of AF1 for binding of other CoRs. Molecules that can interfere with AF1‐CoR interaction surfaces provide an attractive opportunity for novel therapeutic targeting of SHRs for endocrine cancers. Because TATA box binding protein (TBP) is a common binding partner for AF1/NTD of all SHRs and does not bind to the AF2/LBD, yet leads to conformational changes, we hypothesize that TBP‐induced disorder‐order transition opens AF1 surfaces for its interactions with specific coactivators including steroid receptor coactivators‐1 (SRC‐1), a classical SHR coactivator in several endocrine cancers. Consistent with this concept, our data support that structural reorganization of SHRs, AF1/NTD induced by TBP, enhances binding of SRC‐1 to AF1. Further, TBP and SRC‐1 acts synergistically to stimulate AF1‐dependent activity. Targeting ID proteins by small molecules to block protein‐protein interactions is a rapidly evolving field, and our findings suggest that compounds that could block AF1‐TBP interaction sites may provide potential avenues to modify AF1 activity needed for additional selectivity to target cell‐tissue specific gene regulations of SHRs, which could complement or replace existing SRMs actions in current SHR‐based endocrine cancer therapies.

## Controlling apoptotic cell death by regulating mitochondrial pore formation

**C. Hockings^1,2^, A. Alsop^1^, S. Iyer^1^, R. Uren^1^, M. Shi^1^, E. Lee^3^, W. D. Fairlie^3^, G. Dewson^4^ & R. Kluck^1^**

*^1^Walter and Eliza Hall Institute, Molecular Genetics of Cancer Division, Parkville, Australia; ^2^Cambridge University, Department of Chemical Engineering and Biotechnology, Cambridge, UK; ^3^Latrobe University, Olivia Newton‐John Cancer Research Institute, Heidelberg, Australia; ^4^Walter and Eliza Hall Institute, Cell Signalling and Cell Death Division, Parkville, Australia*

*Email:* kluck@wehi.edu.au

Bak and Bax are pore‐forming members of the Bcl‐2 protein family. Both proteins undergo a series of conformation changes before forming dimers that then assemble into the clusters that promote pore formation. An effective means of either promoting or inhibiting apoptosis may be to target helix 1, as tethering this helix to other regions of the protein prevents activation and pore formation. Moreover, dissociation of helix 1 by antibody binding to the helix 1–2 loop triggers conformational change and pore formation. Once dimers form, their assembly to form pores appears not to involve stable protein‐protein interactions between dimers, so this step may be difficult to target therapeutically. Before homodimers form, activated Bak can be sequestered by prosurvival Bcl‐2 proteins such as Mcl‐1 and Bcl‐xL, and this sequestration blocks pore formation. This mechanism of apoptosis resistance is referred to as “Mode 2”, but there are no structures of full‐length activated Bak or Bax bound to their prosurvival guardians, and limited means of quantitating these heterodimers. To better understand the structure of Mode 2 complexes and the role of Mode 2 in resistance to cancer therapies. The formation and disruption of Mode 2 complexes of Bak in mitochondria and in cells was examined using a range of biochemical assays based on our knowledge of Bak conformation change during apoptosis. We have identified new means of detecting Bak in complex with Mcl‐1 or Bcl‐xL. Using these assays, Mode 2 was generated when apoptotic triggers (e.g. Bid) were efficient at activating Bak but had low affinity for Mcl‐1 or Bcl‐xL. Mode 2 was not generated if the BH3‐only protein was truncated and thus inefficient at targeting to mitochondria to activate Bak. Improved measures of Mode 2 may help ongoing efforts to target this step to promote or inhibit apoptosis.

## ARTS protein regulates apoptosis and tumor suppression by causing degradation of both XIAP and Bcl‐2

**N. Edison, D. Mamriev, N. Paland, J. Kagan & S. Larisch**

*Biology Department University of Haifa, Haifa, Israel*

*Email:* saritlarisch@gmail.com

ARTS (Sept4_i2) is a mitochondrial pro‐apoptotic protein which promotes apoptosis by directly binding and degrading the two major proteins inhibiting apoptosis‐ X‐linked Inhibitor of Apoptosis Protein (XIAP), and B‐cell lymphoma 2 (Bcl‐2). ARTS is localized to the outer mitochondrial membrane and initiates caspase activation upstream of mitochondrial outer membrane permeabilization (MOMP). ARTS antagonizes XIAP via a mechanism that is distinct from all other known IAP‐antagonists. ARTS acts as a tumor suppressor protein in mice and humans. In particular, its expression is significantly reduced in leukemia, lymphoma and hepatocellular carcinoma (HCC) patients. Sept4/ARTS‐Null mice show accelerated spontaneous tumor development, elevated XIAP (but not cIAP1) levels, and increased numbers of stem cells that are resistant to cell death. Furthermore, the tumor and apoptosis phenotypes of *Sept4*/ARTS‐deficient mice are suppressed by inactivation of XIAP, indicating that this protein is a major physiological target for the pro‐apoptotic and tumor suppressor activity of ARTS. ARTS functions as a dual antagonist of both XIAP and Bcl‐2, two major negative regulators of apoptosis. We have generated ARTS‐based small molecule mimetics which were found to reduce the endogenous levels of XIAP and Bcl‐2 in cancer cells and promote apoptosis. As levels of both these anti‐apoptotic proteins are high in many types of cancer, these ARTS mimetics may be useful for cancer therapy.

## Metabolic regulation of gemcitabine resistance in pancreatic cancer

**S. K. Shukla**

*Eppley Institute for Research in Cancer and Allied Diseases, University of Nebraska Medical Center, Omaha, NE, USA*

*Email:* surendra.shukla@unmc.edu

Pancreatic ductal adenocarcinoma (PDAC), which represents about 85% of all pancreatic cancer, is the fourth leading cause of cancer‐related deaths in the United States. Pancreatic adenocarcinoma is moderately responsive to gemcitabine‐based chemotherapy, the most widely used single‐agent therapy for pancreatic cancer. Although the prognosis in pancreatic cancer remains grim in part due to poor response to therapy, the regulation of therapeutic resistance in pancreatic cancer cells is not well understood. We observed that increased glycolytic flux leads to glucose addiction in cancer cells and a corresponding increase in pyrimidine biosynthesis. Increased synthesis of nucleotides diminishes the effective level of gemcitabine by molecular competition. Furthermore, we have demonstrated that MUC1‐mediated HIF‐1*α* stabilization plays the key role in metabolic adaptation mediated gemcitabine resistance. We observed that targeting HIF1‐*α* or de novo pyrimidine biosynthesis in combination with gemcitabine strongly reduced the tumor burden in the different mouse models of pancreatic cancer. Finally, we have observed that reduced expression of pyrimidine biosynthesis genes strongly correlates with better prognosis in pancreatic cancer patients treated with fluoropyrimidine analogs. Overall, our studies demonstrate the existence of a novel metabolic mechanism that mediates chemotherapy resistance in pancreatic cancer and provides novel targets to improve the efficacy of gemcitabine in patients.

## Targeting the RING domain of Mdm2‐Mdm4 E3 ligase for cancer cell apoptosis

**X. Wang**

*Department of Pharmacology and Therapeutics, Roswell Park Cancer Institute, Buffalo, NY, USA*

*Email:* xinjiang.wang@roswellpark.org

The RING domains of Mdm2 and Mdm4 interact with each other to form heterodimer E3 complex essential for p53 polyubiquitination and degradation. Beyond p53 regulation, Mdm2‐Mdm4 complex also regulates non‐p53 substrates including pRB. In mouse models, Mdm2 RING domain promotes lymphomagenesis independently of p53. Clinically, Mdm2 overexpression together with mutant p53 predicts the worst prognosis for lymphoma patients after first line treatment. Therefore, Mdm2‐Mdm4 E3 complex is an excellent drug target. Current strategies are focused on targeting Mdm2‐p53 interaction for p53‐activation‐based therapies. However, these inhibitors will not inhibit the activity of Mdm2‐Mdm4 toward non‐p53 targets and will not be useful for p53 mutant patients. To fill this significant gap, we sought to identify small molecule inhibitors that inhibit the E3 ligase activity of Mdm2‐Mdm4 by high throughput screening. We successfully identified several lead compounds including MMRi64 that disrupts the RING‐RING Interaction of Mdm2‐Mdm4 E3 complex. MMRi64 kills leukemia cells by apoptosis. Importantly, p53 is not required for MMRi64 to induce apoptosis. Data from other MMRi64 analogs suggest that MMRi is a potent apoptosis inducer in chemotherapy‐resistant leukemia cells. Therefore, this new type of E3 ligase inhibitors may have potential for further clinical translation.

## Tumor suppressor Fbw7*α* negatively regulates CDX2 protein turnover through ubiquitin‐mediated proteasome degradation

**A. K. Trivedi**

*Department of Biochemistry, CSIR‐Central Drug Research Institute, Lucknow, Uttar Pradesh, India*

*Email:* arun3vedi@cdri.res.in

Ubiquitin‐proteasome system (UPS) plays key role in normal cell biology and disease pathogenesis where he has particularly focused on substrate specific E3 ubiquitin Ligase Fbw7, a known tumor suppressor in cancer. His research has identified new substrates of Fbw7 not reported earlier that include GCSFR, CDX2 and RUNX2. His studies demonstrated that elevated expression of mutant GCSFR in myeloid leukemia cells are due to the fact that it escapes Fbw7‐mediated degradation. In yet another study they Identified CDX2, a master regulator of colon cell differentiation as a substrate of Fbw7 in colon cancer. Fbw7 promotes ubiquitin‐mediated proteasome degradation of CDX2 through two phosphodegron motifs present at N‐ and C‐terminus of CDX2 in a GSK3*α*‐dependent manner. Their finding demonstrated that higher expression of oncogenic CDX2 in some of the colon cancer types is due to loss of FBW7‐mediated CDX2 protein turn over.

## Isoform specific role of akt in oral squamous cell carcinoma

**N. K. Roy & A. B. Kunnumakkara**

*Cancer Biology Laboratory & DBT‐AIST International Laboratory for Advanced Biomedicine (DAILAB), Department of Biosciences & Bioengineering, Indian Institute of Technology (IIT) Guwahati, Assam, India*

*Email:* kunnumakkara@iitg.ernet.in

Oral cancer is the most common cancer in India which kills over 100,000 people in our country annually. Late diagnosis and lack of efficacious biomarkers and therapies are the main issues associated with the management of this disease. Therefore, there is an urgent need for developing novel biomarkers and targets for the effective management of this disease. It is now well established that Akt/ mTOR pathway is highly upregulated in oral cancer and the main risk factors of this disease, tobacco and its components activate this pathway in different cancers. The main component of this pathway exists in three different isoforms such as Akt1, Akt 2 and Akt3. However, the isoform specific role of Akt in oral cancer is poorly understood. In the present study we examined the expression and role of different isoforms of Akt in oral cancer. Our results suggest that Akt 1 and 2 are upregulated in oral cancer but not Akt 3. We also found that silencing of Akt 1 and 2 induced cell proliferation, survival, invasion and metastases of oral cancer cell lines. Collectively, our data indicate that Akt1 and 2 are the major players in oral cancer and can be used as a target to discover novel therapies for this disease.

## Circulatory miRNAs as potential biomarkers for lung cancer

**A. Khandelwal & A. Jain**

*Department of Animal Sciences, Central University of Punjab, Bathinda, India*

*Email:* aklank.jain@cup.edu.in

Worldwide lung cancer is the most commonly diagnosed cancer, and in India, it is the most common cause of cancer related death in males. The disease is frequently diagnosed at advanced stages, resulting in an overall 5‐year survival rate of less than 15%. Conversely, if diagnosed in the early stages of the disease and thereafter, receiving effective treatments, the 5‐year survival rate could possibly be increased to 85%. Therefore, it is reasonable to assume that early detection of lung cancer could reduce mortality. Moreover, the current diagnostic measures taken such as the sensitivity of chest X‐ray and sputum cytology for early detection of lung cancer are low. Therefore, taking advantage of recent developments in molecular genetics for the diagnosis and prognosis of lung cancer is clinically important. And as such, blood plasma is obviously a preferred choice for development of such diagnostic markers. Currently, numerous tumor‐specific molecular alterations have been identified in plasma and shown their potential as biomarkers in patients with lung cancers. However, none of the tested markers thus far had sufficiently achieved the required characteristics for the diagnosis of lung cancer. In this regard, we are investigating the role and expression of circulatory miRNAs in lung cancer patients. Circulating microRNAs (miRNAs) represent stable and reproducible markers for numerous solid tumors, including lung cancer, and have been hypothesized as non‐invasive diagnostic markers for the several cancers. We have observed significant change in expression level of some miRNAs in lung cancer patients compared to healthy controls. We have also observed the altered expression of miRNAs in patients undergoing chemo and radiotherapy treatments.

## Novel ways of perturbing oncogenic interactions for cancer therapy

**A. Nag**

*Department of Biochemistry, University of Delhi, New Delhi, India*

*Email:* alonag22@gmail.com

Cancer continues to be one of the major threats to human beings and accounts for one of the leading causes of death worldwide. This fatal disease is essentially caused by uncontrolled proliferation of abnormal cells. Since most of the anti‐cancer drugs affect the rapidly dividing cells, these drugs also suffer from several undesired side effects. Hence, there is a pressing need to innovate target specific interventions that can help us eliminate the problems associated with non‐specific cellular interactions. To this end, our recent research goal has been to reveal the key molecular mechanisms of Human Papillomavirus induced cellular transformation that can be exploited to discover promising anti‐cancer molecules. Emerging evidences suggest that SUMOylation pathways are hugely exploited to support viral replication, viral assembly and to evade host immune system. We are investigating the mechanisms involved in manipulation of host SUMO system by high risk HPV. We show for the first time that the human coactivator protein hADA3 is posttranslationally modified by SUMOylation and HPV16E6 stimulates hADA3 degradation by enhancing its SUMOylation. In another study, we also demonstrate the involvement of HPV16 E7 in modulating SUMOylation of FoxM1b by impairing its interaction with Ubc9. These findings provide important insight on SUMOylation as a novel mechanism related to HPV mediated transformation of cervical cancer cells. Altogether, these studies are likely to open novel perspectives for future cancer therapy.

## Control of stemness and metastasis by human papillomavirus oncoprotein E6 through hedgehog – GLI signaling in cervical cancer

**A. Bhat^1^, K. Vishnoi^1^, A. Sharma^2^, M. Jadli^1^, T. Singh^1^, D. Pande^1^ & A. C. Bharti^1^**

*^1^Molecular Oncology Laboratory, Department of Zoology, University of Delhi, New Delhi, India; ^2^ExoCan Healthcare Technologies Pvt. Ltd., Pune, India*

*Email:* alokchandrab@yahoo.com

Hedgehog (Hh) GLI‐signaling has been implicated in metastasis and tumor recurrence of human papillomavirus‐mediated cervical cancer. Viral oncoproteins E6/E7 play key oncogenic role in conjunction with the aberrant activation of these cellular signaling events. Our recent observations demonstrate a cross talk between HPV oncoproteins and Hh signaling which synergistically promote stemness in cervical cancer cells. By examining established HPV‐ positive and HPV‐negative cervical cancer cell lines in the presence or absence of specific GLI inhibitor, cyclopamine and HPVE6/E7 siRNAs these cells showed variable but an elevated expression of upstream regulators of GLI‐signaling. Particularly, HPV16‐positive SiHa cells, overexpressed GLI1, Smo and Patch. siRNA‐mediated silencing of HPV16E6 demonstrated reduced GLI1 transcripts in SiHa cells. Cervical cancer stem‐like cells isolated by side population analysis, displayed retention of E6 and GLI1 expression. Further exploration of inductive signaling mediated by exosomes revealed selective aggregation of upstream signaling components of Hh signaling pathway in exosomal fraction irrespective of the cell type. These Hh signaling components‐enriched exosomes when co‐cultured with human endothelial cells (HUVEC) were taken up by the endothelial cells. These observations suggest potential contribution of Hh components in not only altering cancer cell behavior but also may result in modification of tumor microenvironment via exosome trafficking. However, further investigation is needed to establish the later hypothesis.

## Translating cancer genomics to medicine

**A. Dutt**

*Integrated Cancer Genomics Laboratory, ACTREC, Tata Memorial Centre, Navi Mumbai, India*

*Email:* adutt@actrec.gov.in

Massively parallel Next Generation DNA sequencing technologies has made technically feasible to interrogate the complete set of genomic alterations in a tumor in a systematic, comprehensive manner in a single run. These methodologies are beginning to transform diagnostics by allowing cancers to be classified based on molecular mechanism and allowing clinical trials to be undertaken on more homogeneous groups of patients; and, therapeutics by sparking a new generation of drugs targeted at the molecular alterations that cause cancer. Advances made by Dutt Laboratory leading to the establishment of TMC‐SNPdb, first Indian SNP db, to facilitate cancer research using genomics data from Indian origin samples, along with discovery of novel molecular subclasses, new therapeutic targets and biomarkers for clinical development—with detailed mechanistic insights– in head and neck; lung; and, gallbladder carcinoma will be presented.

## ATR mediated control of nuclear mechanics during interstitial migration

**A. Kumar**

*Systems Toxicology & Health Risk Assessment Group, CSIR‐Indian Institute of Toxicology Research, Lucknow, Uttar Pradesh, India*

*Email:* amitkumar@iitr.res.in

Genome instability is a hallmark of cancer, as DNA breaks are major drivers of genome alterations. To govern chromosome integrity, the cells have the devised DNA damage response (DDR), which acts as checkpoint to maintain the genome structure intact. This damage response is primarily mediated by ATR (Ataxia telangiectasia and Rad3 related), DNA‐PK (DNA‐dependent protein kinase) and ATM (Ataxia telangiectasia mutated) and, which react to distinct types of DNA damage. Specific factors assist ATM and ATR recruitment to the DNA lesions. ATR is essential prevents fragile site expression and protects the integrity of replicating chromosomes, aberrant condensation events. Although ATR involvement was long proposed in a wide range of cellular processes independent of DNA damage, such as thermal shock, osmotic stress and mechanical stress, experimental data supporting this hypothesis has been reported only recently. Consistently, ATR localizes in organelles such as centrosomes, nucleoli, and mitochondria. Since compromise in nuclear envelope (NE) integrity could lead to a number of human pathologies including cancer, ATR function at the NE becomes essential for the maintenance of the NE and of NE‐attached chromatin integrity and cell physiology. We recently reported that ATR coordinates chromatin condensation with nuclear envelope breakdown, thereby regulating prophase to metaphase transition. ATR function at the NE becomes vital because the NE of a cell is continuously exposed to forces exerted by external stimuli on the cytoskeleton or internally by changes in chromatin architecture (inducing topological constraints) during many intracellular processes including DNA replication. I will present the advances in ATR research in this direction.

## Mitochondrial uncoupling promotes an aggressive and radio‐resistant tumor phenotype by switching metabolism in glioma cells

**A. N. Bhatt, Y. Rai, S. Singh, S. Pandey, D. K. Sah, B. G. Roy & B. S. Dwarakanth**

*Institute of Nuclear Medicine and Allied Sciences, Timarpur, Delhi, India*

*Email:* anbhatt@yahoo.com, anant@inmas.drdo.in

One of the most common signatures of highly malignant gliomas is their capacity to metabolize glucose to lactic acid than normal brain tissue, even under normoxic condition (Warburg effect), indicating that aerobic glycolysis is constitutively upregulated through genetic or epigenetic changes. However, oxidative phosphorylation (OXPHOS) is also required to maintain the mitochondrial membrane potential for tumor cell survival. In the process of tumorigenesis, tumor cells during fastest growth rate exhibit both high glycolytic and high OXPHOS. In recent past, it is shown that mitochondria in such cells are mildly uncoupled by over expression of mitochondrial uncoupler protein (UCP2), which reduces oxidative stress burden. Therefore, metabolically reprogrammed cancer cells with combination of both aerobic glycolysis and altered OXPHOS develop robust metabolic phenotype which confers selective growth advantage. Therefore, in our study we grew the high glycolytic BMG‐1 (glioma) cells with continuous exposure of mitochondrial uncoupler 2,4 dinitrophenol (DNP) to obtain phenotype of high glycolysis with enhanced altered OXPHOS. We found that OXPHOS modified BMG (OPM‐BMG) cells has similar growth rate and cell cycle distribution but high mitochondrial mass and functional enzymatic activity than parental cells. In *in‐vitro* study OPM‐BMG cells showed enhanced invasion, proliferation and migration properties. Moreover it also showed enhanced angiogenesis in Matrigel plug assay. Xenografted tumor from OPM‐BMG cells showed reduced latent period, faster growth rate and nearly 5 fold reduction in tumor take in nude mice compare to BMG‐1 cells, suggesting that robust metabolic phenotype facilitates tumor formation and growth. OPMBMG cells which was found radioresistant, showed enhanced radiosensitization by 2‐DG as compared to the parental BMG‐1 cells. This study suggests that metabolic reprogramming in cancer cells enhance the potential of migration, invasion and proliferation. It also strengthens the cancer cells to escape the death processes, conferring resistance to therapeutic modalities. Our data also suggests that combining metabolic inhibitors like 2DG with conventional therapeutic modalities can sensitize such metabolically aggressive cancer cells more than the therapy alone.

## Cell death mechanisms of experimental anti cancer therapeutics

**A. Srivastava**

*King George's Medical University, Lucknow, Uttar Pradesh, India*

*Email:* ashutoshshrivastava@kgmcindia.edu

Programmed Cell Death (PCD) is a cell suicide program critical to evolution, development and tissue homeostasis. PCD is classified according to the morphology of a dying cell. Apoptosis is a type I PCD involving Nuclear DNA fragmentation, activation of caspase cascade and phosphotidylserine inversion. More recently, autophagy, a process traditionally considered a cell survival mechanism is also implicated as a mode of PCD and classified as type II PCD. This type of cell death is characterized by excess de novo–synthesized, double membrane enclosed vesicles that engulf and degrade cellular components. Relationship between apoptotic and autophagic death is controversial. Autophagy and apoptosis can be triggered by overlapping signaling mechanisms. These pathways may cooperate, coexist, or antagonize each other to balance death versus survival signaling. It has been shown that increased autophagic activity exists in the cells undergoing death in response to chemotherapeutic drugs. Existence and complex interplay of both apoptosis and autophagy increases the challenges of cell death induction and may lead to decreased efficacy of anticancer drugs coupled with increased survival and drug resistance. There is an increased demand to characterizing more precisely the manner by which a drug kills cancer cells. Such studies will help define the optimal applications of candidate and established drug as a cancer therapeutic.

## Development of anticancer chemotherapeutics through modulation of microtubule dynamics

**A. S. Negi**

*CSIR‐Central Institute of Medicinal and Aromatic Plants, Lucknow, Uttar Pradesh, India*

*Email:* arvindcimap@rediffmail.com

Over the years cancer has become a threat to public health sector due to its high morbidity and mortality. It has imposed a heavy toll on society. The present anticancer chemotherapeutics are associated with severe side effects and drug resistance. In addition to the devastating effects on patients and their families, the economic costs of cancer are enormous. The treatment is beyond the affordability of a common man. The World Health Organization (WHO) has projected that the global number of deaths from cancer will increase by nearly 80% by 2030. Among the various approaches being used for cancer therapy nowadays, chemotherapy is known to be the most effective. It is still a challenge for researchers to develop an effective, safer and affordable anticancer chemotherapeutics. Antitubulin is one of the most successful approaches to treat various types of cancers. There are several clinically important microtubule stabilizers (i.e. paclitaxel, docetaxel) and microtubule destabilizers (i.e. vincristine, vinblastine etc.). Both types of antitubulins disturb microtubule dynamics and misdirect the formation of a functional mitotic spindle. Antitubulin agents induce G2/M phase cell arrest which leads to apoptosis of the tumour cells. We have designed several antitubulin agents taking structural learning from natural antitubulins. Taking ‘*Fragment Based Drug Discovery (FBDD) approach*’ we found a fragment i.e. 3,4,5‐trimethoxyphenyl motif inducing antitubulin effect when introduced at an appropriate position of a pharmacophore. Several anticancer pharmacophores were designed and developed as possible microtubule destabilizers. The basis of designing, synthesis and pharmacology will be discussed in detail. Antitubulin fragment and some of the designed anticancer pharmacophores

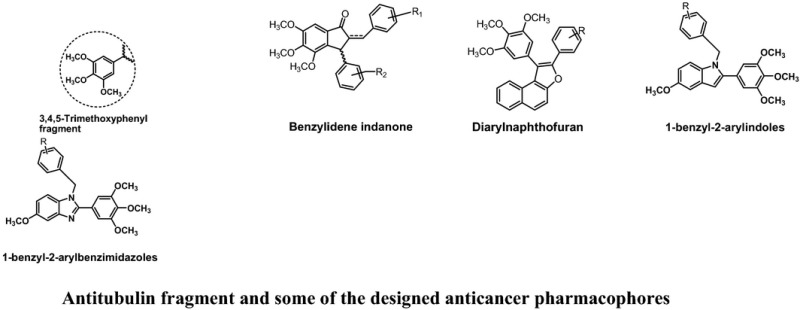

## Induced protein degradation: an emerging therapeutic approach for cancer chemotherapy

**A. Gupta**

*Department of Medicinal Chemistry, CSIR‐Central Institute of Medicinal and Aromatic Plants, Lucknow, Uttar Pradesh, India*

*E mail:* atisky2001@yahoo.co.in

The Induced protein degradation of target protein responsible for cancer has emerged as potential drug discovery paradigm. This approach exploits cellular quality control machinery to selectively degrade target proteins thereby limiting high systemic drug exposure. This concept lead to the development of Fulvestrant (ICI 182,780), a steroidal selective estrogen receptor down regulator (SERD). As estrogen receptor (ER) signalling is crucial for breast cancer progression, ER‐positive breast cancer carcinomas are responsive to anti‐hormones such as Tamoxifen, for treatment of ER+ breast cancer. However, these agents are known to develop resistance in due course of time. Fulvestrant (ICI 182,780) is able to destabilize ER and eventually degrade ER. The peculiarity of Fulvestrant over other antihormonal agents is that the Fulvestrant induced degradation affects the ligand‐independent functions of ER which are not addressed by Tamoxifen thus making it effective under tamoxifen resistance. The importance and recent highlights this approach will be discussed in the presentation.

## Micro‐(RNA)managing SPINK1‐positive subtype of prostate cancer

**B. Ateeq**

*Molecular Oncology Lab, Department of Biological Sciences & Bioengineering, Indian Institute of Technology Kanpur, 208016, Uttar Pradesh, India*

*Email:* bushra@iitk.ac.in

Prostate cancer (PCa) is a clinically heterogeneous disease with marked variability in patient outcomes and prognosis. Overexpression of Serine Peptidase Inhibitor, Kazal type‐1 (*SPINK1*) represents the second most common (˜10–15%) subtype, after highly recurrent (˜50%) androgen‐driven *TMPRSS2‐ERG* genetic rearrangement. Nonetheless, the molecular mechanism underlying SPINK1 upregulation in cancer is poorly understood. We have shown that miR‐338‐5p and miR‐421 post‐transcriptionally regulate *SPINK1* expression. Moreover, ectopic expression of miR‐338‐5p and miR‐421 in SPINK1‐positive PCa cells attenuate oncogenic properties, epithelial‐to‐mesenchymal transition and cancer stemness. Also, stable overexpression of these microRNAs in SPINK1‐positive PCa cells exhibit reduced tumor growth and distant metastases in murine xenograft model. Mechanistically, we show that Polycomb group member EZH2 confers H3K27me3 repressive marks on the regulatory regions of miR‐338‐5p and miR‐421, leading to epigenetic silencing of these miRNAs in *SPINK1‐*positive subtype. Further, we also establish the functional interplay between *SPINK1*, long non‐coding RNA *MALAT1* and miR‐338‐5p/miR‐421, wherein *MALAT1* interacts with EZH2, which in turn are targeted by miR‐338‐5p/miR‐421, thus reinforcing a repressive molecular circuitry. Thus, restoring the expression of miR‐338‐5p and miR‐421 by using either epigenetic drugs or synthetic mimics could abrogate SPINK1‐mediated oncogenesis. Taken together, the present study for the first time unravels the molecular mechanism underlying SPINK1 overexpression in cancer and thereby opens new avenues for the treatment of SPINK1‐positive malignancies.

## Targeting cancer stem cells for relapse free survival of cancer patients

**B. C. Das**

*Amity Institute of Molecular Medicine & Stem Cell Research, Amity University Uttar Pradesh, Noida, Uttar Pradesh, India*

*Email:* bcdas@amity.edu

High risk human papillomavirus (HR‐HPV) oncogenes E6 & E7 are responsible for oncogenic transformation of epithelial cells in the basal layers of uterine cervix leading to development of invasive cervical cancer. Interference of keratinocyte differentiation by these viral proteins may confer stem like properties to these cells but the crosstalk between viral oncoproteins and stem cell signaling is not clearly understood. We, therefore, have isolated and enriched cervical cancer stem like cells by sequential gating from HPV+ve and HPV‐ve human cervical cancer cell lines (SiHa, HeLa and C33a) using a set of functional and phenotypic markers (ABCG2, CD49f, CD71, CD133). The small subpopulation of cervical cancer stem‐like cells (CaCxSLCs) showed spheroid forming ability and expressed pluripotency, quiescence and self‐renewal markers with high expression of HPVE6 and Hes1. The enriched CaCxSLCs were highly tumorigenic and did recapitulate the primary tumor histology in nude mice. Knocking down of HPVE6 by RNA interference resulted in abolition of spheroid formation through downregulation of Hes1 and induction of re‐differentiation while interference of Hes‐1 expression resulted in reduced cervicosphere formation suggesting loss of self‐renewing ability. It indicates a potential regulatory role of HPVE6 in maintenance of cervical cancer stem cells through Hes1 expression. Most interestingly, a high binding activity of AP‐1 and differentially higher expression of majority of AP‐1 family proteins in cervical cancer have been observed and it is established that AP‐1 is essential for viral oncogenes E6/E7 expression. AP‐1 thus appears to be one of the important targets playing critical role not only in tumorogenesis but also maintaining chemo‐radioresistance of cancer stem cells. We have used a herbal compound curcumin which down regulates expression of not only HPV oncogenes and AP‐1 but also majority of signaling pathways and oncogenes associated with the development of cervical cancer except Fra‐1 which is highly upregulated. Following curcumin treatment that upregulated Fra‐1, both cancer and cancer stem cells become highly sensitive to treatment of radiation and common cancer drug such as Doxorubicin. The results indicate that Fra‐1 appears to be a potential therapeutic target that promotes chemo‐radiosensitivity of cervical cancer stem cells which are responsible for drug resistance, tumor relapse and metastasis. In order to target the cancer stem cells, a novel small molecule bioconjugate involving curcumin, folic acid and anticancer drug has been developed for making cancer treatment most effective and recurrence free survival.

## A multifaceted anticancer agent: a ray of hope to overcome relapse and resistance

**C. Mandal, S. Maiti, S. Mondal & E. M. Satyavarapu**

*Cancer Biology and Inflammatory Disorder Division, CSIR–Indian Institute of Chemical Biology, Kolkata, India*

*Email:* chitra_mandal@yahoo.com cmandal@iicb.res.in

Chemotherapy is a major therapeutic approach for the cancer treatment. However, it suffers from selectivity and multidrug resistance. There are urgent needs of better chemotherapeutics to fight against cancer. Deregulation of different signaling pathways is mainly responsible for various types of cancer formation. Usually, drugs that block one pathway failed to block the other hence the cancer cells escape by upregulating other survival pathways. Our aim was to develop non‐toxic, new herbal chemotherapeutics that might counteract different cancers by targeting multiple pathways to inhibit the major cell survival pathways. We identified a carbazole alkaloid, CM‐5 from an edible Indian medicinal plant that showed cytotoxicity against ten different cancer types including more than 30 cell lines having different mutations with minimal toxicity in normal cells. Next, we searched for the major targeted pathways and signaling events altered by CM‐5. CM‐5 showed crosstalk between Apo‐1/Fas signaling via mitochondrial pathways in leukemia. It is a potent inhibitor of mitochondrial complex III in ETC by which enhanced ROS was produced to induce various cellular events for apoptosis. CM‐5 destabilizes Hsp90 chaperone activity through ROS in pancreatic cancer. In GBM, induced ROS triggered DNA damage response, Chk1/Chk2 activation leading to G0/G1 cell cycle arrest even in hypoxic condition. It targets both mTORC1/2 and also inhibits hedgehog pathway mediated survival of cancer stem‐like cells. Our *in vitro*/*in vivo* data established the double‐edged role of CM‐5 which inhibited autophagy and induced apoptosis/anoikis in ovarian cancer to avoid metastasis. It synergistically enhances the cytotoxicity of 5‐fluorouracil/cisplatin/paclitaxel through activation of PTEN, p53/p73 in colon carcinoma and STAT3 inhibition in cervical cancer. Taken together, we have established that CM‐5 as a potent anticancer agent which can overcome drug resistance/relapse by targeting major cell survival pathways and cancer stem‐like cells ultimately giving a hope that it can be potentially used for treatment.

## Epigenetic modulator EZH2 regulates CSC properties and dictates metastatic landscape of cancer

**D. Datta**

*Department of Biochemistry, CSIR‐Central Drug Research Institute, Lucknow, Uttar Pradesh, India*

*E‐mail:* dipak.datta@cdri.res.in

Drug resistance, tumor relapse, metastasis are the major reasons of cancer related death worldwide and it has seemingly become unequivocal that for all these three consequences, the major culprit is a very small portion of specialized cells present in the whole solid tumor mass, known as Cancer stem cells (CSCs). Understanding the biology of these cells in terms of therapeutic interventions are the hotspots of current cancer biology research. Harnessing the capability of Salinomycin to target CSCs, we observed that its stem cell specific cytotoxic action relies on the expression of polycomb group protein, histone methyltransferase—enhancer of zeste homolog 2 (EZH2) which functions as a transcriptional repressor of many pro‐apoptotic genes, responsible for conferring drug resistance in CSCs. Overall EZH2 gain in and loss of function studies in *in‐vivo* xenograft tumor models clearly indicate that EZH2 regulates tumor initiating capabilities and alters metastatic landscape of solid tumors.

## Overexpression of DNA ligase I compensates for Topoisomerase I inhibition leading to resistance in human colorectal cancer cells

**P. Maurya, S. Gupta, S. Krishna, M. I. Siddiqi, K. V. Sashidhara & D. Banerjee**

*Molecular and Structural Biology, CSIR‐CDRI, Lucknow, Uttar Pradesh, India*

*Email:* d.banerjee@cdri.res.in

Our lab is interested in targeting DNA repair proteins to exploit cancer cell‐specific alterations in genome maintenance pathways and to study the increased/decreased dependence upon a specific DNA repair pathway. Since the currently available treatment options are insufficient for the proper treatment of cancers, we need to look closely at the DNA repair pathways that allow cancer cells to live and propagate with multiple mutations, leading to chemotherapy resistance. DNA ligase proteins play crucial roles in almost all DNA replication and repair processes. They catalyze the joining of DNA nicks by the formation of phosphodiester bonds between two adjacent DNA strands. Interestingly, in addition to the nick joining activity, we have now verified that human DNA ligase I also possess DNA relaxation activity, just like topoisomerase I. We, therefore, hypothesize that hLigI may be involved in the development of resistance towards topoisomerase I inhibitors and propose that appropriate hLigI inhibitors when used in combination with Top1 inhibitors, may be useful for the successful treatment of topoisomerase resistant colorectal cancers. We observed an overexpression of hLigI upon treatment of DLD‐1 cells with topoisomerase I inhibitors (camptothecin and topotecan). We then identified a potent new hLigI inhibitor (compound 27) that can inhibit both the nick joining and DNA relaxation activities of hLigI. Thereafter, we have tested the combination of topotecan with compound 27 and found very encouraging additive effects in DLD‐1 cells. Similarly, combination studies done with topotecan and siRNA against hLig1 also showed additive effects in DLD‐1 cells. Therefore, this study predicts a possible cause for development of topoisomerase‐resistance in colorectal cancer cells and how to tackle the resistance.

## Identification of key transcriptional regulatory network driving glioblastoma

**D. K. Singh**

*Systems Toxicology & Health Risk Assessment Group, CSIR‐Indian Institute of Toxicology Research, Lucknow, Uttar Pradesh, India*

*Email:* dk.singh@iitr.res.in

Recent whole‐genome sequencing in cancer patients has identified several (tens to hundreds) activating mutations in oncogenes and suppressive mutations in key tumor suppressor genes. It has been observed that inter‐patient heterogeneity mutational frequency for a given cancer is no different than the inter‐cellular heterogeneous mutational frequency and brain cancer is no exception. In spite of the identification of several druggable targets, clinical benefits, however, have been elusive. Recently we identified a transcription factor regulatory network that is independent of upstream activating mutations and cell types. Our result suggests that core regulatory transcription factors are necessary and sufficient to drive brain cancer irrespective of the initiating mutations (oncogenes, or tumor suppressors) and cell types and this might be a recurrent theme in various cancers.

## Polymorphism in drug metabolizing genes as biomarkers for predicting susceptibility to tobacco induced cancers

**D. Parmar^1^, V. Yadav^1^, F. Hasan^1^, S. Singh^2^, M. L. B. Bhatt^2^, R. Hadi^3^ & T. Katiyar^1^**

*^1^Developmental Toxicology Division, CSIR‐Indian Institute of Toxicology Research, Lucknow, India; ^2^Department of Radiotherapy, King George's Medical University, Lucknow, India; ^3^Department of Radiation Oncology, Dr. R.M.L Institute of Medical Sciences, Lucknow, India*

*Email:* dparmar@iitr.res.in

Head and neck squamous cell carcinoma (HNSCC) and lung cancer are among the most common tobacco induced malignancies that involves a combination of exposure to the carcinogens as well as inheritance of genetic differences in the enzymes catalyzing their metabolism and detoxification. Our studies have shown that variant genotypes of drug metabolizing enzymes such as cytochrome P450s (CYPs) and glutathione S‐transferases (GSTs) modify the risk to tobacco induced squamous cell carcinoma of head and neck (HNSCC) and lung. Prevalence of certain CYP haplotypes (haplotypes of CYP1A1, 1B1 and 2E1) and combinations of genotypes of CYPs and GSTs in cases demonstrated the importance of gene‐gene interactions in determining the risk to these malignancies. Likewise, several fold increase in the risk in tobacco users (both in the form of chewing or smoking) have indicated the importance of interaction of environmental risk factors in determining the risk to the malignancy of head & neck or lungs. To determine if the susceptible genotype changes are associated with phenotypes of increased resistance or susceptibility, studies were initiated to identify the expression and regulation of PAH‐metabolizing CYP1A1 and 1B1 isoenzymes in freshly prepared rat peripheral blood lymphocytes (PBL) isolated from the control and cases suffering from lung cancer. RT‐PCR studies demonstrated significantly higher mRNA expression of polycyclic aromatic hydrocarbon (PAH)‐responsive CYP1A1 isoenzyme in the freshly prepared PBL isolated from lung cancer cases. This increase in the mRNA expression was found to be associated with an increase in the catalytic activity of CYP1A1 in freshly prepared PBL. Interestingly, the variant genotypes of CYP1A1 in the controls were found to be associated with much higher increase in the expression of blood lymphocyte CYP1A1 when compared to those with wild type genotypes. Further, the cases with variant genotypes of CYP1A1 showed much higher increase in the mRNA expression of CYP1A1 and its associated activity in the freshly prepared PBL when compared to the controls. These studies have led us to conclude that CYPs could not only be used as a tool to predict susceptibility to environment induced malignancies but, importantly, integrating expression and functional data with genetic deficiencies may allow more precise identification of biomarkers that may help to identify true risks of exposure and diseases.

## Metabolic reprogramming as a target for anti‐angiogenic therapy

**P. Gupta, A. Shukla, R. Singh & D. P. Mishra**

*Department of Endocrinology, CSIR‐Central Drug Research Institute, Lucknow, Uttar Pradesh, India*

*Email:* dpm@cdri.res.in

Metabolic reprogramming in cancer cells in response to targeted therapies regulate drug resistance, disease progression and relapse. The glycolytic metabolism is considered a promising therapeutic target to curb tumor associated angiogenesis. 2‐DG, a synthetic glycolytic inhibitor is currently under clinical evaluation as a promising anti‐cancer agent. However, 2‐DG treatment in cancer cells activates pro‐survival Akt signaling, that might limit its clinical efficacy. The NADPH oxidase 4 (Nox‐4)/ROS/Akt signaling is known to regulate survival, proliferation infiltration and invasion in glioblastomas (GBMs). The enhanced motility, invasiveness and therapy resistance in GBMs is attributed to metabolic adaptation through increased aerobic glycolysis. Therefore, we hypothesized that inhibition of the Nox‐4 might enhance 2‐DG induced suppression of glycolysis, migration and invasion in GBM cells. We identified the natural naphthoquinone compound Shikonin as a potent inhibitor of the Nox‐4/Akt signaling pathway. The combined treatment of shikonin+2‐DG suppressed the glycolytic phenotype, migration and invasion through modulation of the Akt/HIF1*α*/HK‐2 signaling axis in GBM cells. The combination also exhibited enhanced anti‐proliferative and anti‐angiogenic effects *in vivo*. Our data for the first time demonstrates that inhibition of the Nox‐4 associated pro‐survival signaling pathway by shikonin enhances the anti‐proliferative and anti‐angiogenic potential of 2‐DG in GBM cells. In summary, the combined inhibition of Nox‐4 and glycolysis associated with metabolic reprogramming may have therapeutic implications for the management of GBMs.

## Trastuzumab (Herceptin): benefits and treatment challenges in subtypes of breast cancers

**F. Malik**

*CSIR‐Indian Institute of Integrative Medicine, Jammu, Jammu and Kashmir, India*

*E mail:* fmalik@iiim.res.in

Breast cancer is a heterogeneous disease and has been categories in different subtypes based on the surface expression of hormone receptors estrogen receptor (ER^+^), progesterone receptor (PR^+^); and human epidermal growth factor 2–neu(HER‐2), which are routinely used for diagnostic and treatment purpose in patients. Molecular profiling of breast cancer has further sub categorized it in Luminal cancers (ER+ and PR+), HER2+ and Triple negative (ER, PR, HER2 negative) cancers. About 20% of women with breast cancer have tumors labeled HER2‐positive based on the amplification of HER2 gene and are given treatment of HER2 blocking antibody Herceptin. Although Herceptin has been one of the most useful therapy in the treatment of these patients, our study have demonstrated that it can be useful to other patients of luminal origin with non‐amplified HER2 gene expression, which are clinically not approved for Herceptin treatment. Utilizing breast cancer cell lines, mouse xenograft models, we demonstrate that HER2 is selectively expressed in the cancer stem cell population (CSCs) of ER^+^, HER2^‐^ luminal breast cancers and were responsive to Trastuzumab treatment. In yet another observation in HER2^+^ patients, it has been observed that long term Herceptin treatment causes acquired resistance in these patients. In cell line and mouse models, we found that inflammatory signals with aberrated *PTEN* expression develop resistance to Herceptin treatment. In conclusion, our studies demonstrated that while Herceptin treatment can be useful against Luminal cancers, it was explored that one of the reasons of developing resistance against HER2+ patients can be due to feed back inflammatory signals.

## Phosphorylation of NUMB promotes self‐renewal and chemoresistance of cancer stem cells

**H. R. Siddique & G. G. H. A. Shadab**

*Molecular Cancer Genetics & Translational Research Lab, Section of Genetics, Department of Zoology, Aligarh Muslim University, Aligarh, Uttar Pradesh, India*

*Email:* hrsiddique@gmail.com

Normal Stem cell is propagated through self‐renewing division in which one daughter cell commits to the differentiated cell while the other retains the multipotent characteristics of its parent. The cell fate molecule NUMB plays a crucial role in maintaining the normal homeostasis of both stem cells as well as differentiated cells. However, a deviation is observed in Cancer Stem Cells (CSCs) where the cell fate molecule NUMB symmetrical divided into two daughter cells. Thus, CSCs are defective in their control of the asymmetric division. Symmetric division of CSCs is thought to sustain self‐renewal and recurrence of cancer after therapy. However, the molecular mechanism is not yet clear. As the molecular mechanisms governing self‐renewal and chemoresistance of CSCs are poorly understood, the present study aimed to identify the molecule/s governs this process. Human and mouse CSCs, cell lines, mouse model and clinical samples were used in this study. Self‐renewal assay, protein‐protein interaction, colocalization, promoter activity and protein expressions, kinase activity, were determined by using Spheroid assay, immunoprecipitation, confocal microscopy, reporter assay, immunoblot, immunohistochemistry, and radioactivity assay. For tumorigenicity studies, castrated‐athymic mice bearing CSC tumors were used. We demonstrate that the Phosphorylation of NUMB leading to p53 proteolysis and deregulated self‐renewal and tumor‐initiation property of CSCs. Further, inhibition of NUMB phosphorylation successfully inhibited Tumor development in a mouse model. We suggest that Post‐translational modification of NUMB plays a crucial role in Cancer Stem Cells self‐renewal and Chemoresistance and targeting NUMB post‐translational modification could be an ideal target for therapeutics to treat chemoresistant cancer in humans.

## Telomerase targeted anticancer vaccines

**J. Kailashiya**

*Institute of Medical Sciences, Banaras Hindu University, Varanasi, Uttar Pradesh, India*

*Email:* jyotsna.kailashiya@gmail.com

Telomerase maintains telomere lengths and plays a crucial role in development and survival of cancers cells. It is absent in most somatic cells but is found in stem cells, germ cells and around 90% of cancers, thus making it a selective target for anticancer therapy. Telomerase, a HLA class‐I antigen, is able to stimulate cell mediated immune response by inducing cytotoxic T‐cells. Using this property, many vaccine approaches (like GV1001, HR2822, Vx‐001, GX‐301) have been developed to target cancer cells having telomerase by host's own immune system. Many approaches and studies including clinical trials have shown effective anticancer responses of these vaccines, without toxicity to non cancer cells.

## N6‐methyladenosine landscape of mRNAs in glioma: Essential role of METTL3 and m^6^A modification in glioma stem cell growth

**K. Somasundaram**

*Department of Microbiology and Cell Biology, Indian Institute of Science, Bangalore, Karnataka, India*

*Email:* ksomasundaram1@gmail.com

Despite advances in biology and therapeutic modalities, existence of highly tumorigenic glioma stem cells makes glioblastomas (GBMs) invincible. N6‐methyl adenosine (m^6^A), one of the abundant mRNA modifications catalyzed by *met*hyl*t*ransferase‐*l*ike *3* and *14* (METTL3/14), influences various events in RNA metabolism. Similar to DNA and histone modifications, mRNA modifications like m^6^A are also reversible and play key role in regulation of molecular events. We found that METTL3 and the associated m^6^A modification are essential for glioma stem cell growth. Towards identifying the target of METTL3 in promoting glioma stem cell growth, we carried out an integrated RIP‐sequencing and RNA‐sequencing of METTL3‐silenced GSCs. This analysis revealed several interesting facts. We found that majority of m^6^A modified transcripts are high abundant mRNAs, most of which were found downregulated in METTL3 silenced cells suggesting a role for m^6^A modification in mRNA stabilization. We also found an important role m^6^A modification in RNA splicing, RNA editing, expression of noncoding RNAs and efficient expression actively transcribed genes in GSCs. We also found that SOX2, a glioma reprogramming factor, as the METTL3 target in promoting glioma stem cell growth. The structural requirement and functional consequence of m^6^A modification of SOX2 mRNA and its relevance to METTL3‐mediated radiation resistance was further investigated in detail. Thus our study reports the importance of m^6^A modification in glioma stem cell growth and uncovers METTL3 as a potential molecular target in GBM therapy.

## MicroRNA: a new potential marker for prostate cancer

**M. K. Ahmad^1^, M. Waseem^1,2^, M. Serajuddin^2^, A. A. Mahdi^1^, S. N. Sankhwar^3^ & D. P. Mishra^4^**

*^1^Departments of Biochemistry, King George's Medical University, Lucknow, India; ^2^Department of Zoology, Lucknow University, Lucknow, India; ^3^Departments of Urology, King George's Medical University, Lucknow, India; ^4^Department of Endocrinology, CSIR‐CDRI, Lucknow, Uttar Pradesh, India*

*Email:* kaleembaksh@gmail.com

Prostate cancer is maximally diagnosed cancer still it is the second leading cause of death in western world. So, there is urgent need of a new biomarker for diagnosis as well as therapy purpose. MicroRNAs (miRs) are noncoding RNA which regulate about 30% of human genes. Due to their stability, sensitivity and specificity in sample these miRs are current hot topic of research in the field of biomarker hunt. In the present study, we evaluated differentially expressed microRNA in benign prostatic hyperplasia (BPH), prostate cancer (PCa) on and control subjects by Microarray analysis. Most significantly up regulated microRNAs were validated through RT‐PCR analysis. Our result showed that miR‐1827, miR‐4510 and miR‐130b‐3p were over expressed in prostate cancer as compared to control as well as BPH. MiR‐1827, miR‐4510 was also found to be up regulated in high Gleason score and metastasis tissue. These miRs showed good specificity and sensitivity in receiver operating characteristic (ROC) analysis which may prove helpful in differentiating between prostate cancer and Control. High expression of miR‐1827 correlated with the poor prognosis and low progression free survival in PCa subjects in comparison to low expression of miR‐1827. The putative microRNAs, miR‐1827, miR‐4510 and miR‐130b‐3p will be used as diagnostic marker and also for therapeutic purpose to overcome prostate cancer burden in coming scenario.

## DNA‐dependent protein kinase plays a central role in transformation of breast epithelial cells following alkylation damage

**M. Lahiri**

*Indian Institute of Science Education and Research, Pune, Maharashtra, India*

*E mail:* mayurika.lahiri@iiserpune.ac.in

DNA alkylating agents form the first line of cancer chemotherapy. They not only kill cells but also behave as potential carcinogens. *N*‐methyl *N*‐nitrosourea (MNU), a DNA methylating agent, is well known to induce mammary tumours in rodents. However, the mechanism of tumorigenesis is not well understood. Our study reports a novel role played by DNA dependent protein kinase (DNA‐PK) in methylation damage‐induced transformation using three‐dimensional breast acinar cultures. We report that exposure of breast epithelial cells to MNU inhibited polarisation at the basolateral domain, increased dispersal of the Golgi at the apical domain and induced an epithelial‐to‐mesenchymal transition (EMT)‐like phenotype as well as invasion in the acinar cultures. The altered Golgi phenotype correlated with impaired intracellular trafficking. Inhibition of DNA‐PK resulted in almost complete reversal of Golgi dispersal and partial rescue of the polarity defect as well as EMT‐like phenotype. Our results confirm that methylation damage‐induced activation of DNA‐PK is a major mechanism in mediating cellular transformation.

## Epithelial‐to‐mesenchymal transition and its clinicopathological implications in patients with urothelial carcinoma of the bladder

**M. Garg**

*Department of Biochemistry, University of Lucknow, Lucknow, Uttar Pradesh, India*

*E mail:* minal14@yahoo.com

Epithelial‐to‐mesenchymal transition (EMT) is a dynamic physiological process involved in the pathogenesis of urothelial carcinoma. It involves the phenotypic transition of non‐motile epithelial cells to migratory and invasive mesenchymal cells. In the present study, the expression of EMT markers was investigated to evaluate its prognostic and diagnostic significance in patients with non muscle‐invasive bladder cancer (NMIBC) and muscle‐invasive bladder cancer (MIBC). Real‐time quantitative polymerase chain reaction (RT‐qPCR) and immunohistochemical (IHC) staining was performed in a cohort of 80 human bladder tumor tissues obtained from archives of Department of Pathology, SGPGIMS, India. Epithelial markers (E‐cadherin), mesenchymal markers (N‐cadherin and Vimentin), EMT‐activating transcription factors (Snail, Slug, Twist and Zeb) were examined for their expression at transcriptome and protein level followed by their correlation with histopathological features and their statistical relevance using SPSS 20.0 software. At transcriptome level, statistical relevance was reported between reduced expression of E‐cadherin and high grade (*P* = 0.001; One sample T‐test); enhanced expression of N‐cadherin with high grade (*P* = 0.035; Independent sample T‐test) and with low stage (*P* = 0.003; Mann‐Whitney non‐parametric test). Enhanced expression of transcriptional factors Slug and Twist exhibited statistically relevant association with recurrence (*P* = 0.041 and *P* = 0.048 respectively; Independent sample T‐test). According to IHC studies statistical relevance was observed between focal loss of membranous expression of E‐cadherin with presence of hematuria (*P* = 0.007; Mann‐Whitney non‐parametric test). Significant correlation was reported between novel membranous expression of N‐cadherin and high grade tumors (*P* = 0.001; Moses non‐parametric test); novel membranous expression of Vimentin and primary tumors (*P* = 0.001; Moses non‐parametric test). Nuclear immunopositivity of Snail, Slug and Twist had relevant association with low grade tumors (*P* = 0.0042, *P* = 0.0317, 0.0135; Fischer's exact test). Molecular validation of the EMT marker profile can prove to be a sensitive and an effective prognostic tool for objective and systematic investigation of EMT function in the pathogenesis of urinary bladder cancer.

## Potential mechanism of phytochemicals induced apoptosis in cancer cells

**M. Arshad, A. Jafri, Sahabjada, J. Rais, N. Shivnath & V. Rawat**

*Molecular Endocrinology Lab, Department of Zoology, University of Lucknow, Lucknow, Uttar Pradesh, India*

*Email:* arshadm123@rediffmail.com

The phytochemicals are the bioactive compounds present in dietary products such as vegetables, cereal grains, fruits and plant based beverages as well as in medicinal plants. Phytochemicals provide an excellent pool of molecules, associated with prevention and decrease the risk of several types of chronic diseases including cancer. The naturally occurring dietary phytochemicals such as Curcumin, Naringenin, Quercitin, Ellagic acid, Rutin, Piperine, Fiestin, Cucurbitacins and Thymoquinone are abundant in our daily diet. Extensive research on *in vitro* cell culture systems and *in vivo* animal models exhibit that these phytochemicals induce apoptosis in different type of cancer. We have evaluated apoptotic inducing potential of Naringenin, Piperine and Eupalitin on skin carcinoma (A‐431), Oral squamous carcinoma (KB) and prostate carcinoma (PC‐3) respectively *via* various markers of apoptosis and proliferation. Findings of studies suggest that these phytochemicals induce apoptosis through ROS generation, modulation in mitochondrial membrane potential (MMP), nuclear condensation, DNA fragmentation, cell cycle arrest and regulation of caspases. Some molecular markers such as p53, Bcl‐2, and BAX etc. were also modulated by these compounds *via* extrinsic and intrinsic pathway of programmed cell death. Further, studies are needed to validate cancer therapeutic efficacy of these promising phytochemicals at *in vivo* and clinical level.

## Adhesion dependent membrane trafficking. Role and regulation of Ral in cancers

**N. B. Subramanium**

*Indian Institute of Science Education & Research, Pune, Maharashtra, India*

*Email:* adhesion.lab@gmail.com

Most cells in the human body depend on their ability to attach to the extracellular matrix (ECM) to grow, survive and migrate. Integrin mediated adhesion to the ECM regulates downstream signaling to confer anchorage dependence. Cancer cells overcome this regulatory control to become anchorage independent and acquire their unique growth and survival advantage. Our earlier studies have helped reveal how integrin mediated adhesion regulates membrane trafficking to control anchorage dependent Erk, Akt and Rac signaling. On loss of adhesion rapid endocytosis of membrane microdomains through caveolae turns off signaling and re‐adhesion returns them to the plasma membrane using the Ral‐Arf6‐exocyst complex to restore signaling. Oncogenic Ras in cancers is seen to activate Ral to drive this trafficking and promote anchorage independent signaling. Our studies have now helped understand the role and regulation of RalA and Arf6 downstream of adhesion and oncogenic Ras. In doing so we have identified the role and regulation of a novel RalGEF in mediating the RalA activation in normal and cancer cells. We have also evaluated the role Aurora Kinase A has in activating RalA in Ras independent cancers. To this effect, we have developed a novel nanovesicle drug delivery system to better deliver the poorly water‐soluble inhibitor MLN8237 at concentrations that specifically target AKA. This allows us to not only evaluate the AKA‐RalA crosstalk, but inhibit RalA and anchorage independent growth in cancers

## Endothelin receptor antagonism advances proliferation of mouse liver sinusoidal endothelial cells by protection from inflammation‐induced mitochondrial impairment and DNA damage

**N. Yadav^1,2^, F. L. Jaber^1^, Y. Sharma^1^, P. Gupta^1^, P. Viswanathan^3^ & S. Gupta^1,4,5^**

*^1^Department of Medicine, Albert Einstein College of Medicine, Bronx, NY, USA; ^2^Department of Biochemistry, Dr. RML Avadh University, Faizabad, India; ^3^Department of Pediatrics, Albert Einstein College of Medicine and Children's Hospital at Montefiore, Bronx, NY, USA; ^4^Department of Pathology, Albert Einstein College of Medicine, Bronx, NY, USA; ^5^Marion Bessin Liver Research Center, Diabetes Center, Irwin S. and Sylvia Chanin Institute for Cancer Research, Ruth L. and David S. Gottesman Institute for Stem Cell and Regenerative Medicine Research, Albert Einstein College of Medicine, Bronx, NY, USA*

*Email:* neelam2k4@gmail.com

The potent vasoconstrictor, endothelin (ET‐1), activates tissue inflammation‐related clearance of transplanted cells from microcirculations, which hampers cell therapy. Transplantation of endothelial cells is relevant for coagulation factor replacement, vessel damage, tissue engineering and also organ regeneration. If ETA/B receptor inactivation protected endothelial cells, this would be beneficial. We tested ETA/B receptor antagonist, bosentan, in mouse liver sinusoidal endothelial cells (LSEC) isolated from healthy donors. Cytoprotection was examined with assays *in vitro*. The ability of transplanted LSEC to engraft and proliferate was studied in DPPIV‐ mice. Transplanted LSEC engrafted in the liver of DPPIV‐ mice after hepatic preconditioning. Engraftment of LSEC with inactivation of ETA/B receptors through incubation with bosentan was superior. In LSEC treated with bosentan, viability, adhesion to extracellular matrix components, and mitochondrial membrane potential improved, including the presence of hypoxia and inflammatory cytokines/chemokines/receptors. This allowed accelerated proliferation in transplanted bosentan treated LSEC during liver repopulation. We discovered bosentan‐treated LSEC were protected from oxidative DNA damage due to lower levels of reactive oxygen species and from double‐strand DNA breaks with preservation of ATM expression. Antagonism of ETA/B receptors protected endothelial cells by multiple intracellular mechanisms. Overcoming adverse ET‐1 effects by this means will be significant for liver regeneration and other applications of endothelial cells. Extensive experience with ETA/B antagonists will help translate this mechanism in people.

## Pro‐apoptotic hsa‐miR‐23a˜27a˜24‐2 as therapeutics for breast cancer

**N. Saini**

*CSIR‐Institute of Genomics and Integrative Biology Mall Road, New Delhi, India*

*Email:* nsaini@igib.res.in

With ever increasing incidences of drug resistance and tumor recurrence, current research is focusing on exploring out safer treatment options. One such option are microRNAs that are endogenous non protein coding small RNAs (22–24 nt), known to be involved in regulation of various cellular processes such as differentiation, proliferation, metastasis & apoptosis. In our laboratory we have identified and characterized hsa‐miR‐23a˜27a˜24‐2 cluster which induces apoptosis in human embryonic kidney cells via JNK. Gene expression profiling was used to characterize the transcriptional response to miR‐23a˜27a˜24‐2 cluster overexpression in HEK293T cells and we observed that this cluster induced apoptosis is mediated by induction of ER stress. Simultaneously we also made an attempt to explore the anti‐cancer potential of individualistic members of this miR‐cluster. Using target prediction algorithm (TargetScan) we identified 12 target genes common among the individualistic members of the hsa‐miR‐23a˜27a˜24‐2 cluster. Subsequently, *in silico* protein‐protein interaction analysis using cytoscape software identified 3 significant clusters, of which NCOA1 (SRC1), NLK, and RAP1B formed important hub nodes. Functional annotation of these genes revealed their role in endocrine signaling, MAPK signaling and cytoskeletal reorganization pathways, all of which jointly converge into regulation of metastasis. For validation of these predictions revealed that individualistic members of pro‐apoptotic hsa‐miR‐23a˜27a˜24‐2 cluster family functions as tumor suppressor by attenuating metastasis and can be further evaluated as therapeutic modalities against breast cancer *in vivo*.

## Differential expression of transcript isoforms related to diagnosis and prognosis of cancers

**N. Singh & M. L. B. Bhatt**

*Department of Molecular Biology, Centre for Advanced Research, King George's Medical University, Lucknow, India*

*Email:* neetuaashi@yahoo.com,neetusingh@kgmcindia.edu

Research has shown that the tens of thousands of human genes contain hundreds of thousands of exons, which produce hundreds of thousands of different transcript isoforms. These transcript isoforms are produced through various combinations of the exons of a gene that translate to mRNA. The splicing mechanism involved includes exon skipping, mutually exclusive exons, alternative 5’ donor sites, alternative 3’ acceptor sites and intron retention. This splicing mechanism contributes to myriad of transcripts which translates to proteome diversity. Aberrant splicing mechanism may lead to the development of cancer and dysregulated splicing signatures of cancers may act as prognostic predictors. Till date however, the dysregulated splicing patterns identified through genome‐wide screening mechanisms have until recently been less well‐studied. However, recently many cancers have identified subtype specific differentially spliced genes and splice isoforms like most common types of breast tumors TNBC, non‐TNBC and HER2‐positive breast cancer (Horvath et al 2013). Recently Li et al in 2017 have made prognostic predictors based on alternative splicing events with high performances for risk stratification in NSCLC patients and uncovered interesting splicing networks in LUAD and LUSC which could be underlying mechanisms. Currently, we are investigating splicing signature for Non small cell lung carcinoma (NSCLC)‐squamous and adenocarcinoma; four sub‐groups of Medulloblastoma; and Tyrosine kinase sensitive and resistant‐Chronic Myeloid Leukemia (CML) cases. Through this we will be able to efficiently differentiate and identify markers for therapeutic response between NSCLC‐ squamous and ‐adenocarcinoma; among Wnt‐, SHH‐, Group‐3‐ and Group‐4‐Medulloblastoma; and between TKI sensitive and TKI‐resistant CML cases.

## *β*‐hCG: A key player for BRCA1 defective tumorigenesis

**P. Srinivas**

*Cancer Research Program, Rajiv Gandhi Centre for Biotechnology, Thiruvananthapuram, Kerala, India*

*Email:* priyasrinivas@rgcb.res.in

Human Chorionic Gonadotropin (hCG), a heterodimeric molecule with *α* and *β* subunits, is mainly secreted during pregnancy by the trophoblastic cells to promote implantation of the embryo. But studies have revealed that the *β*‐subunit of hCG (*β*‐hCG) has an independent function and is frequently associated with non‐ trophoblastic malignant tumors, mainly in ovarian, prostate and breast cancers, although its exact role during tumorigenesis is still not understood. Reports suggest that *β*‐hCG can inhibit apoptosis or stimulate the growth of cancer cells and that the elevated serum level of *β*‐hCG correlates with aggressiveness of cancer. Further, it has been shown that *β*‐hCG promotes the invasion of prostate cancer cells by activating the expression of ERK1/2 and MMP‐2 and decreasing the expression of E‐cadherin in prostate cancer cells and showed poor prognosis of the disease. Interestingly, anti *β*‐hCG vaccines have been developed and found to be active against cancer cells expressing *β*‐hCG *in vitro*. Controversially it has also been found that *β*‐hCG can induce apoptosis in breast cancer. Thus *β*‐hCG expression in breast cancer is highly controversial. Here we analyzed the role of *β*‐hCG in BRCA1 defective triple negative breast cancer. Our study has identified for the first time that *β*‐hCG expression is linked to BRCA1 status and its over expression is seen in BRCA1 mutated (5382insC) but not in wild type triple negative breast cancer cells. BRCA1 conditional knockout mouse breast cancer tissue exhibited over expression of *β*‐hCG while BRCA1 expressing human breast cancer tissues did not express *β*‐hCG. Further, wild type but not the mutant BRCA1 directly represses the expression of *β*‐hCG. Presence of *β*‐hCG either due to endogenous expression or by exogenous supplementation promotes tumorigenesis by inducing migration, invasion and stemness predominantly in BRCA1 mutant breast cancer cells. Taken together, our results provided evidence that there exists a reciprocal regulation between BRCA1 and *β*‐hCG in inducing tumorigenesis in BRCA1 defective triple negative breast cancer. We urge inhibiting *β*‐hCG could prove an effective treatment strategy for BRCA1 mutated breast cancers.

## Fisetin causes DNA damage and apoptosis through ROS in human gastric cancer cells

**A. Sabarwal & R. P. Singh**

*Cancer Biology Laboratory, School of Life Sciences, Jawaharlal Nehru University, New Delhi, India*

*Email:* rana_singh@mail.jnu.ac.in

The current therapy fails to generate significant antitumor effect due to a defective apoptotic machinery or drug resistance in gastric cancer cells. Thus, new and alternative approaches are required to be explored which can help overcome the abnormality. Phytochemicals have shown promising activities against various cancers. Fisetin is a plant polyphenol from the flavonoid group. Its action against gastric cancer has not been investigated in detail. We identified that fisetin, possesses selective apoptosis inducing activity against gastric cancer cells. It also caused G1 phase cell cycle arrest and decreased the levels of G1 phase cyclins and CDKs. Increase in apoptotic cells was accompanied with mitochondrial membrane depolarization. Fisetin caused DNA damage along with gamma‐H2A.X(S139) phosphorylation and cleavage of PARP. ROS as well as cellular nitrite and superoxide generation were increased. Pre‐treatment with N‐acetyl cysteine (NAC) blocked ROS generation and also caused protection from fisetin‐induced DNA damage. The formation of comets were observed in only fisetin treated cells which was blocked by NAC pretreatment. NAC and ascorbic acid pretreatment has also blocked fisetin mediated cell death. Further exploration of the source of ROS, using mitochondrial respiratory chain (MRC) complex inhibitors, it was found that fisetin caused ROS generation specifically through complex I. Collectively, these results for the first time demonstrated that fisetin possesses anticancer potential through ROS production most likely *via* MRC complex I leading to apoptosis in human gastric carcinoma cells.

## Role of Gfi‐1 in long term–hematopoietic stem cells (LT‐HSC) and leukemia stem cells self ‐renewal and differentiation

**S. K. Singh**

*Stem cells/Cell Culture Lab, Centre for Advance Research, King George Medical University, Lucknow, Uttar Pradesh, India*

*Email:* satsaiims@gmail.com

Growth factor independent factor‐1 (Gfi‐1) gene is a zinc‐finger DNA binding protein that functions primarily as transcriptional repressor. Gfi‐1 is required for the proper development of hematopoietic cells. Mice lacking Gfi‐1 (Gfi‐1^−/−^) are viable, but have reduced lifespans (>100 days). Gfi‐1 KO mice show additional hematopoietic phenotypes including reduced numbers of HSC, impaired B and T cell development, and erythroid and myeloid hyperplasia. Gfi‐1^−/−^ HSPC show increased cycling, and loss of HSC activity was observed when Gfi‐1^−/−^ bone marrow cells (BMC) were serially transplanted or competitively transplanted with normal BMC, suggesting that increased cycling of HSC leads to HSC differentiation and exhaustion. Thus, Gfi‐1 has been proposed to function to restrict HSC proliferation and maintain HSC quiescence. Mutation in the zinc finger domains of Gfi‐1 results in hyperplasia and severe congenital neutropenia in humans. Some of the GFI‐1 target genes have been identified, and have been shown to mediate the hematopoietic defects observed in Gfi‐1^−/−^ mice, including the maturational defect in granulocyte development (CSF‐1, RasGRP1, and PU.1), B cell development (PU.1 or Id2), and myeloid hyperplasia (Id2 or HoxA9). Gfi‐1^−/−^ HSPC show increased cell cycling, which suggests that GFI‐1 functions to restrict HSC proliferation and prevent exhaustion. Overexpression of BCL‐2 in Gfi‐1^−/−^ mice partially restored the ability of Gfi‐1^−/−^ BMC to reconstitute TBI recipients, indicating that GFI‐1 maintains HSC, in part, by protecting cells from apoptosis. Overexpression of BCL‐2 alone does not rescue the hyperproliferative defect in Gfi‐1^−/−^ BMC, and these mice do not survive; therefore, the precise mechanism by which GFI‐1 regulates HSC self‐renewal, quiescence and maintenance remains an area of active research. Gfi‐1 directly represses Hoxa9 gene expression. Hoxa9 overexpression triggers myeloid malignancies in mice. Gfi‐1 null mice show features that are reminiscent of AML. They show uncontrolled proliferation of myeloid progenitors that are blocked in their differentiation. Low expression of Gfi‐1 has been found in subtypes of patients with myelodysplastic syndrome. These studies suggest a role of Gfi‐1 in myeloid leukemia. Gfi‐1 expression is very low in early leukemic cells (Blast cells), like FAB subtype M0 and M1 associated with poor prognosis. The precise role of GFI‐1 in human HSPC is not known; therefore, I am evaluating the role of GFI‐1 in human HSPC and leukemia stem cells growth and differentiation. The mechanism by which GFI‐1 regulates HSC self‐renewal, quiescence and maintenance remains an area of active research. AML is very heterogeneous in nature, I am finding association of Gfi‐1 expression in different AML subtype with self‐renewal and differentiation.

## Targeting tumor biochemistry: hope for cancer treatment

**S. K. Trigun, R. K. Koiri, K. B. Singh, B. K. Maurya & L. Mishra**

*Biochemistry Section, Department of Zoology, Institute of Science, Banaras Hindu University, Varanasi, Uttar Pradesh, India*

*Email:* sktrigun@gmail.com, sktrigun@bhu.ac.in

The dominance of molecular biology during the last 2–3 decades overshadowed the importance of basic biochemistry in evaluating cellular targets for viable anticancer agents. Now it is being strongly realized that searching a pharmacological target at cellular/biochemical level is much more relevant than targeting DNA. Growing tumors face hypoxia and therefore, they activate hypoxia induced factor (HIF1*α*) for switching overt to anaerobic mode of survival mechanisms. This mechanism involves a survival signaling cascade initiated by Akt phosphorylation followed by a series of metabolic adaptations acquired by the tumor cells. Thus, in recent years, there is an evolving concept to target the key enzymes and regulatory factors of these pathways. We have worked out extensively on this aspect using Dalton's lymphoma (DL) and hepatocellular carcinoma (HCC) in rodent models. Some ruthenium complexes have been shown to induce apoptosis and to regress DL by declining the level of LDH and iPFK2 *in vivo*. DMSO was also observed to repress the expression of LDH & iPFK2, resulting into induction of TNF‐*α* – p53 led apoptosis of the DL cells *in vivo*. In addition, RT‐PCR, western blotting and IHC data generated from normal, HCC rats suggest that levels of phosphorylated Akt, HIF1*α*, angiogenic and glucose mobilizing factors get up regulated in the HCC cells. However, most of these factors could be normalized due to the treatment with the two natural products; emodin and fisetin. This was consistent with the activation of proapototic factors in the treated HCC cells accompanied with a significant increment in the life span of the HCC rats. These findings strongly advocate that formulation of novel anticancer compounds targeted to the critical enzymes and regulatory factors of tumor supportive metabolic pathways could be the relevant approach in cancer therapy.

## Strategies of Targeting tumor metabolism as a novel anticancer therapeutic approach

**S. M. Singh**

*School of Biotechnology, Institute of Science, Banaras Hindu University, Varanasi, Uttar Pradesh, India*

*Email:* sukhmahendrasingh@yahoo.com

Tumor cells exhibit unique metabolism with respect to the bioenergetics and biosynthetic machinery, which manifests sustained survival with a mammoth proliferative potential, escape from cell death, chemoresistance, and evolution of an ecological niche tumor microenvironment, which promotes tumor progression and metastasis. Thus, targeting of crucial molecular checkpoints of tumor metabolism and tumor growth promoting components of tumor microenvironment lay the foundation for the designing of some novel anticancer therapeutic strategies. Considering the tremendous therapeutic potential of such strategy in combating tumor progression, a number of inhibitors of various aspects of tumor metabolism are being tested for their antitumor properties. Many of such agents include those capable of inhibiting carbohydrate, amino acid metabolism, enzymes of inflammatory pathways, lactate & pH homeostasis, fatty acid synthesis and other important aspects of energy generation in malignant cells. Tumor cells were exposed to different agents capable of targeting pH regulators, monocarboxylate transporters, enzymes catalyzing glycolysis and Krebs’ cycle, cyclooxygenases, and fatty acid synthase both *in vitro* and *in vivo*, followed by analysis of cell survival parameters, and underlying molecular mechanisms of tumor growth retardation and chemosensitization. Exposure of tumor cells to metabolic inhibitors resulted in inhibition of survival, tumor progression accompanied by augmented induction of cell death caused by alteration of pH regulation, inflammation, membrane biosynthesis, angiogenesis, glucose & lactate transport, and carbohydrate metabolism. Such approaches also rendered tumor cells sensitive to cytotoxicity of other conventional anticancer agents by reversal of multidrug resistance. Disruption of tumor metabolism resulted in altered constitution of tumor microenvironment leading to reversal of tumor‐induced immunosuppression. Therefore, we conclude that approaches of reprogramming tumor metabolism and altered constitution of tumor microenvironment will be of immense significance in contributing to the development and designing of novel and more effective tumor‐specific chemotherapeutic regimens and regulation of multidrug resistance.

## Novel regulators of proteostasis

**S. M. Srinivasula**

*Indian Institute of Science Education and Research, Thiruvananthapuram, Kerala, India*

*Email:* sms@iisertvm.ac.in

Protein homeostasis or Proteostasis in cells is attained by precise co‐ordination between diverse and dynamic stress response networks of various intracellular compartments. Improper co‐ ordination, which increases with ageing, is implicated in the development of various pathologies including degenerative diseases. Important components involved in proteostasis are the ubiquitin proteasome and the lysosome‐autophagy systems. Ubiquitin protein ligases (E3) that regulate mitochondrial quality control and clearance of damaged mitochondria also play critical roles in protein homeostasis and development of age‐related disorders, making investigations in to these complex physiological processes a high priority. My talk will cover recent observations that our group has made on role played by hitherto unknown E3s in the removal of damaged mitochondria.

## Histone H2A isoforms in cancer genome: a subtle difference brings major changes in expression profile and phenotype

**S. Gupta**

*Epigenetics and Chromatin Biology Group, Gupta Lab., Cancer Research Institute, Advanced Centre for Treatment Research and Education in Cancer, Tata Memorial Centre, Kharghar, Navi Mumbai, India*

*Email:* sgupta@actrec.gov.in

Histones play an important role in regulating chromatin structure and function through dynamic posttranslational modifications and specialized histones called histone variants and isoforms. Histone H2A variants have been studied in depth and have been found to have distinct functions. Although there are 16 genes for histone H2A isoforms which code for 11 different homomorphous proteins (with sequence divergence of up to three amino acids), but the distinct roles of these isoforms within human cells remains poorly investigated. Moreover, differential expression of H2A histone isoforms in human cancer remains elusive. To investigate the differential expression profile of H2A isoforms in normal and cancer human tissues and their correlation with clinico‐pathological parameters. Recently, we have profiled different H2A isoforms transcripts in eleven different normal tissues. The analysis of H2A isoforms expression revealed predominant expression of specific isoforms in different normal tissues. However, tissue‐specific overexpression of HIST1H2AA was observed only in testis. In testicular cancer, HIST1H2AA was downregulated by more than 10,000‐fold suggesting HIST1H2AA could be a putative biomarker. Further, novel and unexpected H2A isoform gene expression profile was translating into developmental lineage information, suggesting importance of H2A isoforms in early embryonic development. Analysis of H2A isoforms in breast invasive carcinoma patient samples as well as in breast cancer cell lines compared to their normal counterparts showed predominant expression of two isoforms, HIST1H2AC and HIST2H2AC. *In silico* analysis of TCGA data for HIST1H2AC and HIST2H2AC in breast cancer showed comparable findings. The observed alteration in HIST2H2AC in breast cancer patients correlates with tumor grade, and therefore may be helpful in stratification of the disease. Additionally, cancer‐specific expression of H2A isoforms, HIST2H2AC was also observed to be dysregulated in brain and liver cancer tissues compared to normal. In this study, we suggest that histone isoforms provide chromatin with distinctive properties and these alterations promote or even drive cancer development through mechanisms that involve changes in epigenetic plasticity and genomic stability.

## Oncogenic splicing switch and glucose metabolism in breast cancer

**S. Shukla**

*Department of Biological Sciences, Indian Institute of Science Education and Research Bhopal, Madhya Pradesh, India*

*Email:* sanjeevs@iiserb.ac.in

The cancer cells thrive on glucose by converting it to lactate at the end of glycolysis. The phenomenon is known as aerobic glycolysis or Warburg effect and promotes the growth of the cancer cells. The alternative spliced isoform Pyruvate kinase M2 (PKM2) contributes to the Warburg effect by promoting aerobic glycolysis whereas PKM1 isoform promotes oxidative phosphorylation. The *PKM* gene contains two mutually exclusive exons, exon 9 and 10 which are alternatively included in the final transcript to give rise to *PKM1* and *PKM2* isoform respectively. In this study, we report that the intragenic DNA methylation‐mediated binding of BORIS (Brother of regulator of imprinted sites) at the alternative exon of *Pyruvate Kinase* (*PKM*) is associated with cancer‐specific splicing that promotes Warburg effect and breast cancer progression. Interestingly, inhibition of DNA methylation or BORIS depletion or CRISPR/Cas9‐mediated deletion of BORIS binding site leads to splicing switch from cancer‐specific *PKM2* to normal *PKM1* isoform. This results in the reversal of Warburg effect and inhibition of breast cancer cell growth, which may serve as a useful approach to inhibit the growth of breast cancer cells. Importantly, our results show that in addition to *PKM* splicing, BORIS also regulates alternative splicing of several genes in a DNA methylation‐dependent manner. Our findings highlight the role of intragenic DNA methylation and DNA binding protein, BORIS in cancer‐specific splicing and its role in tumorigenesis.

## Identification and characterization of a novel BCL2 inhibitor as a potent chemotherapeutic agent

**S. C. Raghavan**

*Department of Biochemistry, Indian Institute of Science, Bangalore, Karnataka, India*

*Email:* sathees@biochem.iisc.ernet.in

The anti‐apoptotic protein BCL2 is overexpressed in several cancers, and contributes to prolonged cell survival and chemoresistance, lending itself an excellent target for chemotherapeutics. Recently, we described the design and synthesize of Disarib, a novel BCL2 inhibitor. Disarib showed selective cytotoxicity in BCL2 high cancer cell lines, and CLL patient primary cells, over BCL2 low cells. Disarib showed strong affinity to BCL2, but not to other antiapoptotic BCL2 family members. Interestingly, Disarib binding to BCL2 was predominantly BH1 domain dependent, unlike previously reported inhibitors which target BH3 domain. Further, studies using BAK^−/−^ BAX^−/−^ double knockout MEFs demonstrated that Disarib specifically disrupted BCL2‐BAK interaction, but not BCL2‐BAX or other members of the proapoptotic family. Disarib caused cytotoxicity by activating the intrinsic pathway of apoptosis. Importantly, we show that Disarib administration caused tumor regression in mouse allograft and xenograft models, exhibited platelet sparing property and did not show significant side effects. Comparison between Disarib and ABT199, revealed higher efficacy for Disarib in mouse tumor model and cancer cell lines. Interestingly, Disarib showed synergism with paclitaxel, highlighting potential for combination chemotherapy. Thus, we have identified a novel BCL2 inhibitor, which has the potential to be developed as a chemotherapeutic agent.

## Pharmacotherapeutic, genoprotective and antiproliferative efficacy of *Pteris vittata* L

**S. Kaur & P. Kaur**

*Department of Botanical and Environmental Sciences, Guru Nanak Dev University, Amritsar, Punjab, India*

*Email:* sjkaur@rediffmail.com, satwinderjeet.botenv@gndu.ac.in

Cancer occurs due to unique multiple disorders that may arise from unhealthy dietary habits, exposure to environmental and occupational carcinogens, infectious agents and other related risk factors. The search for improved chemopreventive agent continues to be an important line in the discovery of modern anticancer drugs. *Pteris vittata* L. (Pteridaceae) is an ethnomedicinal plant, known as Chinese ladder brake fern which is used as demulcent, tonic and in the healing of cough, cold and fever. In the present study, we aimed to explore the hepatoprotective, genoprotective and antiproliferative properties of six extract/fractions of *P. vittata* fronds. Various fractions were prepared from the 80% methanol extract (PME) mother extract as hexane (PHE), diethyl ether (PDE), ethyl acetate (PEAE), n‐butanol (PNE) and water (PWE) fractions. Among all the fractions tested, PEAE fraction showed potent scavenging ability in *in vitro* antioxidant assays. In the SOS chromotest, PEAE fraction was found effective in modulating the genotoxicity of mutagens AFB1 and B(a)P with an EC50 of 68.49 and 52.82 *μ*g/mL respectively. PNE fraction was found to strongly inhibit the DNA damage induced by 4NQO in Comet assay using human blood lymphocytes. In DNA nicking assay, PHE, PDE and PWE fractions exhibited effectiveness in maintaining the supercoiled form of pBR322 DNA. PEAE fraction was also found to exhibit strong cytotoxicity and apoptosis inducing potential in MCF‐7 cancer cell line. PEAE was also found to exhibit hepatoprotective potential against 2‐Acetylaminofluorene (2‐AAF) induced hepatic damage in male Wistar rats by restoring the normal serum and biochemical parameters. The pretreated PEAE groups of animals showed reduction in the level of p53 immunostaining as compared to the 2AAF treated group. PEAE showed the presence of umbelliferone and epicatechin in appreciable amount as analyzed using HPLC. These findings revealed the potential of *P. vittata* PEAE fraction for its use as chemotherapeutic agents.

## Whole body, tissue, cellular and molecular physiology in a non alcoholic fatty liver disease, a health complication induced by lipoapoptosis. insights from *in vivo* and *in vitro* models

**S. T. Abdullah**

*Indian Institute of Integrative Medicine (I.I.I.M.), Jammu‐Tawi, Jammu & Kashmir, India*

*Email:* stabdullah@iiim.ac.in

The events as sequential pattern that causes nonalcoholic fatty liver Disease (NAFLD) with ensuing inflammation and fibrosing steatohepatitis (NASH) are not completely understood. The present talk will throw some light on the chronology of whole body, tissue, cellular and molecular events that occur during the evolution of NASH. Utilizing various *in vitro* (hepatocyte models) and *in vivo* (small animal models) we have investigated these sequences. The various parameters that will be discussed will be liver histology, body composition, mitochondrial respiration, metabolic rate, gene expression and hepatic lipid composition. Further, lipoapoptosis and autophagy in lipid‐stimulated hepatoma cells will be discussed and therapeutic role of various experimental treatment modalities based on specific signalling pathways that can have a beneficial role in prevention of hepatic fat accumulation and lipotoxicity will further be discussed.

## Cancer chemopreventive efficacy of *Nigella sativa* L. constituents: A molecular and cell target based approach

**S. Luqman & N. Masood**

*Molecular Bioprospection Department and Academy of Scientific and Innovative Research (AcSIR), CSIR‐Central Institute of Medicinal and Aromatic Plants, Lucknow, Uttar Pradesh, India*

*Email:* s.luqman@cimap.res.in

*Nigella sativa* L. (Ranunculaceae), a spiritual plant that God gave to humans, possess divine supremacy to battle out numerous diseases and disorders. The seed of the plant commonly known as ‘Kalonji’, has been described as ‘Panacea’ (cure for all) by the ancient Egyptians; ‘Greek Coriander’ by the Romans and ‘Habbat‐ul‐Barakah’ or ‘Habbah Sawda’ (Blessed seed)’ in Arabic, ‘Hak Jung Chau’ in Chinese and Shoneez in Persian culture. In oldest religious and medical texts, the seeds have been referred to as ‘Melanthion’, widely used in Ayurveda, Siddha Tibbe‐Nabvi and Unani system of medicine throughout the world. The holy Prophet Muhammad quoted black seeds are the remedy for every diseases and cure for everything except death. The Canon of Medicine, an encyclopedia by Avicenna quotes that black seed stimulates body's energy and help recovery from fatigue. Investigations have revealed the extensive therapeutic potential of kalonji with numerous salutary properties including antibacterial, anticancer, antidiabetic, antifungal, antihypertensive, antiinflammatory, analgesic, antioxidant, bronchodilator, diuretic, gastroprotective, immunomodulatory and renal protective etc. Reports suggest that Thymoquinone, a signature phytochemical of this plant, is responsible for most of the healing properties of kalonji seed. Testimonies by the cancer patients have reported that they got rid of cancer by consuming seeds of *N. sativa* for few months. Multiple diseases cured by this sacred plant now hints about the constituents acting in a synergistic or antagonistic manner or individually to have the desired medicinal effect. In this study, our aspiration is to unveil and validate the curing assets of constituents present in *N. sativa* L. emphasizing its cancer chemopreventive properties. The constituents of *N. sativa* (acyclic, monocyclic and bicyclic monoterpenes) were examined for their antiproliferative properties on WRL‐68, Hela, MCF‐7, MDAMB‐231, K‐562 and HEK‐293 cell lines. A dose‐dependent effect (1–50 *μ*g/mL) was observed in MTT, SRB and NRU assays. Molecular and cell target based studies were performed at a concentration of 10 *μ*g/mL with best result were noticed in leukemic cell lines for thymoquinone. The assessment of activity was validated through real time expression analysis as well as molecular docking studies. In cell cycle checkpoints, thymoquinone and limonene were found to be effective against mad2 delete cells of *S. pombe*. Thymoquinone also showed 2–7% apoptotic cells in K‐562 and Hela cell lines and prevent the migration of WRL‐68, HeLa and MDAMB‐231 cell lines during 24 h. In *ex vivo* toxicity studies, monoterpenes (10 *μ*g/mL) were found to be protective against osmotic haemolysis of red blood cells and non‐significant changes in body weight and biochemical parameters were observed in balb/c mice. Cyclic monoterpenes were noticed as a better candidate compared to acyclic and bicyclic which may perhaps be due to resonance stabilized benzenoid ring structure. Research work in our laboratory on this sanctified plant is a journey to reveal and substantiate the curative properties of *Nigella sativa* emphasizing its pharmacological properties, therapeutic benefits and future possibilities of its use in contemporary medicine.

## mTOR signaling complex 2 (mTORC2): role in cancer progression and response to therapy

**S. Bhadoria**

*Toxicology and Experimental Medicine, CSIR‐Central Drug Research Institute, Lucknow, Uttar Pradesh, India*

*Email:* smriti_bhadauria@cdri.res.in

The mammalian target of rapamycin (mTOR) an evolutionary conserved Ser/Thr protein kinase belonging to PIKK family, regulates a myriad of cellular processes while being nucleated into at least two functionally and structurally distinct complexes i.e. mTORC1 and mTORC2. While rapamycin inhibits mTORC1, the mTORC2 is insensitive to rapamycin treatment. Though clinical trials using rapalogs demonstrated important clinical benefits in several cancer types, the objective response rates achieved with single agent therapy have been only modest. Furthermore the tumors where PTEN deficient and/or PI3K/AKT activating mutations are prevalent, such as glioblastoma, prostate and breast cancers, significant improvement in response to rapamycin/rapalogs is rarely observed. In fact a large body of data indicates that rapamycin exerts only cytostatic effects and often its use culminates into refractory/resistant tumors. The ineffectiveness of rapamycin as single agent therapy is partly attributable to concomitant mTORC2 activation which eventually accounts for AKT activation and reinstatement of proliferative/survival signaling cascades. In addition mTORC2 directly contributes in cancer progression by promoting specific pro‐tumoral phenomenon such as epithelial mesenchymal transition, cancer cell invasion and M2‐polarization of tumor associated macrophages (TAMs). Collectively, this accounts for emergence of more aggressive relapsed/recurrent cancers. Therefore in order for anti‐mTOR therapy to be efficacious, simultaneous and/or prior inhibition of mTORC2 is well warranted. Our recent studies aim to identify modality for mTORC2 inhibition without perturbing mTORC1. Furthermore we also aim to evaluate if selective mTORC2 inhibition could impede pro‐tumoral cascades and thus attenuate cancer progression and/or improve the response to therapy. We report that Ras‐mSIN1 interaction eventually accounts for mTORC2 activation and disruption of mSIN1‐Ras interaction inhibits Ras mediated mTORC2 signalling. Our findings highlights the importance mSIN1‐Ras interaction in assembly of functionally intact mTORC2 which in turn could serve as basis for designing mTORC2 specific inhibitors to be potentially employed in combinatorial anti‐cancer regimen.

## Role of p53 aggregation in dysregulation of autophagy in cancer

**S. S. Mir**

*Department of Bioengineering, Integral University, Dasauli, Lucknow, Uttar Pradesh, India*

*Email:* smir@iul.ac.in

Cancer is a leading cause of death around the world due to the progressive accumulation of abnormalities in cellular DNA which, in turn, provide a selective growth advantage to cancer cells and facilitate metastatic dissemination. In cancer cells, dysregulation of certain signalling pathways, together with chromosomal abnormalities, have been identified. p53 is one of the most important tumor suppressor protein which functions as a transcription‐factor and suppresses tumorigenesis by regulating cell proliferation and migration. Mutations in the *TP53* gene, usually missense mutations, are very frequent and common feature of cancer and normally associate to a more aggressive disease. These mutations may cause three different effects in the protein‐function: loss of function, dominant negative effect and gain of function. With the advent of mutations, malfunctioning of modification reactions, such as MDM2 ubiquitination, protein transportation, and changes in cellular milieu, the protein stability is compromised. In addition, different mutation types may change p53 structure differently probably resulting in a diverse tendency to aggregate that may lead to sequestration of important regulatory cofactor proteins. Protein misfolding and aggregation have been related to several human disorders, generally termed protein aggregation diseases. In cancer, so far, p53 has been described to form oligomeric‐aggregates *in vitro* but the exact mechanisms are largely unknown. Some recent evidence suggests that in cancerous cells, mutated p53 protein starts to aggregate due to the exposure of aggregation‐nucleating sequence (251–257 amino acids) within the hydrophobic core of the DNA‐binding domain and also co‐aggregated with wild‐type p53 and its paralogs p63 and p73. Thus, the hypothesis that p53 aggregation may be involved in some cancers similarly to the situation in Alzheimer's and Parkinson's disease has attracted increasing attention. In our study, we explored the status of p53 mutation in different cancer cell lines and tried to evaluate its association to formation of p53 protein aggregates in cancer cells. We also used *in‐silico* approach to gain insight into the molecular basic of p53 protein aggregation mechanism by targeting the exposure of aggregation‐prone regions of p53 protein due to mutation. Since p53 protein plays a significant role in the regulation of autophagy and till date, there has been no study to show the impact of protein aggregates on the autophagic pathway in the cancer cells. We evaluated the role of protein aggregation in cancer and its implications on the deregulation of the autophagy pathway. Our study demonstrated that after dissociation of these p53 aggregates by using, Emodin as an aggregate inhibitor, autophagy level gets increased which may be due to the release of autophagy related proteins and p53 protein from the aggregates and restoration of their function. This study helps us to understand the role of *TP53* mutation in protein aggregation in cancer and how these aggregates affect the autophagy pathway in cancer cells. Thus, targeting these p53 protein aggregates in cancer cells open a new avenue for cancer research and can be used in therapeutic aspects in future.

## Targeting VDAC2/BAK axis by a BH3‐only pro‐apoptotic protein BID to induce mitochondrial apoptosis in tumors

**S. S. Roy**

*CSIR‐Institute of Genomics & Integrative Biology, Sukhdev Vihar, New Delhi, India*

*Email:* soumya.roy@igib.res.in

BID is a BH3‐only proapoptotic BCL2 family protein that induces mitochondrial apoptosis by BAK/BAX‐dependent release of cytochrome c and other inter‐membrane space proteins to the cytosol and consequent Caspase 3 activation. The voltage‐dependent anion channels (VDACs) are conserved outer mitochondrial membrane (OMM) proteins that act as primary gates for solutes and ion transport to mitochondria. It is believed that VDACs are the part of mitochondrial Permeability Transition Pore (PTP) however, their role in apoptotic OMM permeabilization remains controversial. In vertebrates VDAC has three isoforms and using isoform specific knockout mouse embryonic fibroblast (MEF) we showed previously that VDAC2 acts as a crucial component in tBID‐induced OMM permeabilization and cell death by allowing the mitochondrial recruitment of BAK. It was previously reported that in normal liver and lung VDAC2 is less expressed. We found that in both these tissues the level of mitochondrial BAK is also very low. Interestingly, the tumors of those tissues show high level of VDAC2 and consequently high level of mitochondrial BAK. Being an OMM resident protein, BAK is instrumental to efficient and robust apoptosis after BID induction. Our data showed that indeed targeting of VDAC2/BAK axis by recombinant tBID or by activating cellular Bid pathway caused higher level of mitochondrial cell death in tumor cells than their normal counterpart.

## Multi‐targeted approach to cancer therapy

**V. Rai, N. Awasthee, S. S. Verma, S. Mishra & S. C. Gupta**

*Laboratory for Translational Cancer Research, Department of Biochemistry, Institute of Science, Banaras Hindu University, Varanasi, Uttar Pradesh, India*

*Email:* sgupta@bhu.ac.in, subhashg167@gmail.com

Cancer is a chronic disease that is caused by the dysregulation of multiple genes and cell signaling pathways. Yet, most of the drugs designed by man (pharmaceutical companies) are based on modulation of more specifically a single target. Thus, these drugs are less likely to be effective and exhibit side effects when consumed over long periods. In contrast, most drugs designed by “Mother Nature” are multi‐targeted, highly effective over long‐term and exhibit minimal side effects. Here, we provide evidence that bharangin, a diterpenoid isolated from *Premnaherbacea* exhibit activities against multiple cancer types including breast cancer. Bharangin sensitizes cancer cells to doxorubicin and gemcitabine. The pro‐inflammatory cytokine induced NF‐*κ*B activation in cancer cells that was suppressed by bharangin. The diterpenoid also suppressed phosphorylation and degradation of I*κ*B*α*, I*κ*B*α* kinase activation, p65 nuclear translocation and p65 phosphorylation all of which are required for NF‐*κ*B activation. Immunoprecipitation and molecular docking studies indicated that bharangin can directly inhibit binding of p65 to DNA. The diterpenoid also suppressed the expression of proteins involved in tumor cell survival, proliferation, invasion and angiogenesis. Interestingly, the genetic deletion of p65 abolished the effects of bharangin. The diterpenoid also induced expression of tumor suppressor lncRNAs (MEG‐3, MHRT, NEAT, GAS‐5), while down‐regulating oncogenic H19 expression. Furthermore, an abundance in the p65 expression was observed in breast tumor tissues. The pro‐inflammatory transcription factor, NF‐*κ*B is known to be regulated by H19 lncRNA. Overall, the suppression of H19 expression and NF‐*κ*B activation by bharangin may contribute to its anti‐cancer activities.

## Strategies of antagonizing anti‐apoptotic proteins to target detrimental cells for regression of liver injuries and carcinogenesis

**S. Sharma^1^, S. M. Ghufran^1^, S. Ghose^2^ & S. Biswas^1^**

*^1^Amity Institute of Molecular Medicine and Stem Cell Research (AIMMSCR), Amity University, Noida, UP, India; ^2^Department of Medical Oncology, All India Institute of Medical Sciences, New Delhi, India*

*Email:* sbiswas2@amity.edu

The violation of cellular checkpoint within cancer cells and initiation of cellular death in hepatocytes trigger by context dependent actions of pro‐ and anti‐apoptotic proteins. Our studies revealed that the apoptosis resistance within harmful cells can be caused by expression of anti‐apoptotic ‘Inhibitor of apoptosis’ (IAP) proteins, which expressed at high level in human hepatoma cells and activated hepatic stellate cells (HSCs).We have investigated the miRNA based strategies to target cell death pathways within tumour cells using xenograft mouse model of hepatocellular carcinoma (HCC). Here we are targeting the detrimental HSCs and cancer cells therapeutically by sensitizing them towards apoptosis without affecting primary hepatocytes for regression of liver injury and HCC. To develop the disease progression model *in vivo* from liver fibrosis to cirrhosis and/or HCC, the C57BL6 mice were treated with carbon tetrachloride (CCl4). Liver fibrosis/ cirrhosis and HCC were measured by H&E and Masson's trichrome staining. Liver damages were confirmed with serum ALT level using Sigma MAK052‐1KT. We found that there were significantly increased level of the pro‐survival ‘Inhibitor of apoptosis’, IAP1, IAP2, X‐IAP, and survivin within HSCs in fibrotic conditions with activation of NF‐*κ*b. But the levels of IAPs were decreased in liver tissue constituting 70–80% hepatocyte cells from CCl4 induced fibrotic livers. IAPs level with fibrosis marker *α*SMA were also tested and compared with human cirrhotic liver biopsy. Human HSCs were more resistant than hepatoma cells with multikinase inhibitor sorafenib due to increase expression of XAF1 with having inhibitory effect on IAP proteins. On the contrary the higher concentration of sorafenib induced cytoplasmic vacuolation and ER stress mediated pathways within HSCs but independent of autophagy. Interestingly the use of IAPs antagonist not only restricts the activation of HSCs inducing cell death pathway but also modulate the macrophages transition *in vivo*, emphasizing the therapeutic implication of IAPs antagonist for immune modulation and to target both HSCs and HCC cells. Targeting IAP proteins based on mimicking IAP‐binding motifs of second mitochondria‐derived activator of caspase (SMAC) is a new therapeutic intervention strategy to cure liver injury and HCC.

## miRNA controlled death and survival of neuronal cells

**S. N. Bhattacharyya**

*RNA Biology Research Laboratory, Molecular Genetics Division, CSIR‐Indian Institute of Chemical Biology, Kolkata, India*

*Email:* suvendra@iicb.res.in

RNA processing bodies or P‐bodies are cytoplasmic RNA granules in eukaryotic cells that regulate gene expression by executing the translation suppression and degradation of mRNAs targeted to these bodies. P‐bodies can also serve as storage sites for translationally repressed mRNAs both in mammalian and yeast cells. Recently, a unique role of mammalian P‐bodies has been documented where depletion of P‐body components dedifferentiate nerve growth factor‐treated PC12 cells whereas ectopic expression of P‐body components induce neuronal differentiation of precursor cells. Trophic factor withdrawal from differentiated cells induces decrease in P‐body size and numbers that are coupled with dedifferentiation and cell death. Here we report how the expression of P‐body proteins, by ensuring phosphorylation of Ago2 and subsequent inactivation let‐7a miRNPs, prevent apoptotic death of growth factor depleted neuronal cells.

## Cancer epigenetics: dietary interventions and metabolic signatures

**P. Mondal, B. S. Somashekar & S. M. Meeran**

*Laboratory of Cancer Epigenetics, Department of Biochemistry, CSIR‐Central Food Technological Research Institute (CSIR‐CFTRI), Mysuru, Karnataka, India*

*Email:* s.musthapa@cftri.res.in

The growing interest in cancer epigenetics is primarily due to the reversible and hereditable nature of epigenetic changes which tend to alter during the course of carcinogenesis. The major types of epigenetic modifications studied in‐depth are DNA methylation, histone modifications and miRNA‐mediated gene silencing in cancer progression. Epigenetic regulation plays a critical role in normal growth and embryonic development by controlling transcriptional activities of several genes. From the last two decades, these modifications have been well recognized to be involved in tumor initiation and progression, which have motivated many investigators to do research on nutri‐epigenomics. Although some synthetic epigenetic inhibitors have been developed for cancer therapy, but their long‐term use is hindered because of their toxicities and the development of chemo‐resistance in patients. Bioactive phytochemicals (tea polyphenols, cucurbitacin B, resveratrol, indole‐3‐carbinol, and sulforaphane, etc.), which are largely non‐toxic, have been tested for their role in epigenetic modulatory activities in cancer prevention and therapy. These phytochemicals have been shown to inhibit the growth of tumors and induce cancer cell death through a various mechanisms including epigenetic modifications. Intriguingly, epigenetic modifications such as DNA methylation and histone acetylation reactions require co‐factors that are derived from various metabolic pathways, including glycolysis, and TCA‐cycle. Some of the bioactive diets have been shown to alter the level of energy metabolites, thereby, amend the epigenetic modification involved in carcinogenesis. Since cancer is a multi‐stage process, it requires multi‐targeted approaches for the development of an effective treatment regimen. Many of the bioactive phytochemicals possess more than one epigenetic target and are thus capable of concomitantly upregulating tumor‐suppressor genes and downregulating tumor‐promoters/oncogenes. Further, exploration of combinations of bioactive phytochemicals having two different epigenetic targets might advance in the development of effective preventive and therapeutic approaches against cancer.

## Phytopharmaceuticals: bridging prevention to therapy

**Y. Shukla**

*Food, Drug and Chemical Toxicology Group, CSIR‐Indian Institute of Toxicology Research, Lucknow, Uttar Pradesh, India*

*Email:* yshukla@iitr.res.in

In the last few decades, phytochemicals have potentially gained as an important place in cancer research as phytopharmaceuticals. The interests for these compounds have grown by researchers, as these compounds have natural in origin and hence, no stipulated side effects are known. Currently, thousands of natural plant based compounds have been screened for their novel efficiency to control cancer cell proliferation. Among these, a large number of natural compounds gained high preventive and therapeutic values against cancer and most potential compounds among of them are Curcumin, Lupeol, Resveratrol, Indole‐3‐carbinol, Genistein, Gingerol, Allyl sulfides, Berberine, Lycopene, Bromelain and polyphenols. In our laboratory, we continuously worked for last few decades to elucidate the potential effects of these compounds in cancer chemoprevention and therapy against many cancer types. Studies included identification of the targets of these potential compounds through analyzing mechanisms of action in the *in vitro* and *in vivo* systems. Our studies evidently showed that several phytochemicals have immense chemopreventive potentials in the *in vivo* models. Gene expression analysis by administrations of suitable doses in the *in vitro* system explored the mechanisms by signaling cascades governing anticancer effects. Continuing these studies, an array of experiments conducted to recognize the efficiency of phytochemicals with common chemotherapeutic drugs. Interestingly, we found the synergistic effects of combinations of phytochemicals with routinely used drugs. The recent research paves more efficiency of natural compounds using nanotechnology applications. The nanotized form of chemotherapy drugs and phytochemicals interestingly delivered with more pronounced effects even at very low doses and also minimized toxicity on non target cells. Our research also showed that the nanotized form of Curcumin, Bromelain, Polyphenol efficiently target the cancer cells. The recent research on phytochemicals towards evaluating the anticancer efficacy has been accelerated by development of biochemical and biophysical technologies. However, several challenges still persist in cancer research like heterogeneity, development of novel phenotypes in cancer cells by therapeutic drugs and tumor recurrence. These challenges open a new dimension for cancer research, and also give weightage to explore the uses of specific phytochemicals in therapy.

## PI3K/AKT Pathway is inhibited in NSCLC cell line A549 by downregulation of EGFR via an HSP‐90 inhibitor Gedunin

**A. Hasan^1^, E. Haque^2^, M. Kamil^2^, J. Fatima^1^, S. Irfan^2^, A. Khatoon^2^, M. Aslam Yusuf^1^ & S. S. Mir^1^**

*^1^Department of Bioengineering, Integral University, Lucknow, India; ^2^Department of Biosciences, Integral University, Lucknow, India*

*Email:* smir@iul.ac.in

Hyperactivation of PI3K/Akt pathway is associated with tumor development, progression, poor prognosis, and resistance to cancer therapies. PI3K and AKT proteins are also known to be stabilized by heat shock protein 90 (HSP‐90). Several studies have shown the antitumor role of HSP‐90 inhibitors as it can target a variety of pathways, and may provide a new strategy for resolving the problem of drug resistance in cancer. This study aims to analyze the role of Gedunin, an HSP‐90 inhibitor, in the mediation of crosstalk between apoptosis and autophagy by targeting EGFR led PI3K/AKT pathway. A549 cells were treated with different concentrations of gedunin and the inhibitory rate of cell proliferation was measured by MTT assay after 24 h of treatment. We further investigated the effect of gedunin to study the hallmarks of apoptosis by different staining methods like DCFH‐DA, DAPI, and MitoTracker. The expression of *EGFR*,* PIK3CA*,* AKT* and the marker genes for apoptosis and autophagy were studied using semi‐quantitative RT‐PCR after treatment with gedunin for 24 h. The results revealed that gedunin exerts cytotoxic effects on A549 cells in a dose‐dependent manner after 24 h of treatment. In our mechanistic study we found that gedunin causes increase in ROS generation, downregulates mitochondrial membrane potential (MMP) and leads to loss in DNA integrity. Semi‐quantitative RT‐PCR analysis revealed that gedunin sensitized A549 cells towards apoptotic cell death. Our results indicate that the cytotoxic potential of gedunin is in concurrence with hallmarks of apoptosis as we observed increase in ROS generation by 8.2 fold, loss in mitochondrial membrane potential (MMP) by 3.6 fold and increase in chromatin condensation by 5.3 fold when compared to untreated control. Further, the RT‐PCR analysis shows that gedunin treatment downregulated the expression of autophagic marker genes and upregulated the expression of apoptotic marker genes. The compound also downregulated *EGFR* expression leading to downregulation of the expression of *PIK3CA* and *AKT* and hence sensitizing A549 cells towards apoptosis. Our study concludes that HSP‐90 inhibition regulates PI3K/AKT signaling pathway, inhibits autophagy and induces apoptosis in lung cancer cells. Thus, our study provides a new strategy for treatment of NSCLC by targeting the EGFR led PI3K/AKT signaling pathway via inhibition of HSP‐90.

## Identification of anticancer lead from the potential of fresh water cyanobacteria *Geitlerinema* sp. CCC728, and *Arthrospira* sp. CCC729

**A. Srivastava**

*CSIR‐National Botanical Research Institute, Rana Pratap Marg, Lucknow, Uttar Pradesh, India*

*Email:* akankshamukund@gmail.com

Cancer is the second leading cause of death globally, and was responsible for 8.8 million deaths in 2015. The number of new cases is expected to rise by about 70% over the next 2 decades. Approx 14 million new cases per year of the world's total cancer cases occur in Africa, Asia, and Central and South America. A number of chemotherapeutic agents (60% of the approved drugs) for cancer are sourced from natural compounds. Due to increase in the number of cancer patients and limitations of the available drugs, problems of drug safety, narrow spectrum of activity and effectiveness, the development of newer broad‐spectrum anticancer molecules from the less explored natural sources, is desirable. This led concern to explore natural drug resources, such as the less explored fresh water filamentous cyanobacteria. Five freshwater, non‐heterocystous, filamentous cyanobacterial strains (*Phormidium* sp. CCC727, *Geitlerinema* sp. CCC728, *Arthrospira* sp. CCC729, *Phormidium* sp. CCC731 and *Leptolyngbya* sp. CCC732 were screened for their anticancer potential by using human colon adenocarcinoma (HT29) and human kidney adenocarcinoma (A498) cancer cell lines, along with normal rat kidney cells (NRK 52E) as a control. The Bio‐Plex Pro human cancer biomarker panel, cell cycle analysis, and a calcein‐based cell viability assay were also used. Cancer cell lines adopted (HT29 and A498) showed deformities, cell disaggregation in morphological features after exposure to fractions of *Geitlerinema *sp. CCC728 and *Arthrospira* sp.CCC729. On comparison with normal NRK52E and normal MCF‐10A cells, the fractions of these cyanobacteria indicated a possible source of anticancer molecules. Flow cytometric analysis also showed cell cycle arrest in S and G2/M phase, suggesting of antiproliferative nature. of the extracts. Apoptotic analysis of A498 and MCF‐10A cells clearly demonstrated that these fractions induced apoptosis in A498 in contrast to MCF‐10A cells. Bio‐plex Pro human cancer biomarker assay indicated presence of anticancer biomolecules by observing the down/ up regulation of marker proteins. It is demonstrated that freshwater cyanobacteria are also potential source of anticancer biomolecule. A combined strategy of genetic screening may prove worth and identification of cyanobacteria as an anticancer drug resource.

## miRNA‐128 coordinates cholesterol metabolism by regulating CYP7A1 mediated bile acid biosynthesis

**A. Chandra & N. Saini**

*Functional Genomics Unit, CSIR‐Institute of Genomics and Integrative Biology, New Delhi, India*

*Email:* amit.chandra@igib.res.in

MicroRNAs are a class of small non coding RNAs that play important role in various developmental and physiological processes. A state of intervened miRNA expression can be potentially linked with significantly altered proteome between the normal and diseased tissues and may be successfully utilized for diagnosis and prognosis of cancer. Proliferation, migration, and invasion of cancer cells are correlated with serum cholesterol profile and cholesterol lowering drugs, such as statins affecting HMGCR, LDLR and ABCA1 were proved to retard the progression of many cancer types such as breast, prostate, and ovarian cancers. Several micro RNAs including miR‐26a, miR‐33 and miR‐122 are reported to be critically involved in regulation of cholesterol metabolism along with cell proliferation and cell cycle progression by targeting crucial intermediates. The present study aims for elucidation of miR‐128 mediated network behind cholesterol metabolism and symptomatic reversal of hypercholesterolemia. Hypercholesterolemic mice model was developed and intra‐peritoneal injection of microRNA‐128 anti‐sense oligonucleotide (ASO) were given. Microarray analysis of hypercholesterolemic mice liver tissue has been done. We have validated our results with qRT‐PCR and western blotting. Histopathological conditions have been demonstrated by Oil red O and H&E staining. In our previous studies, we have shown that dietary cholesterol uptake significantly alters the expression of hepatic microRNA‐128 levels suggesting that miRNA‐128 might well be involved in regulating cholesterol homeostasis. *In vivo* silencing of miR‐128 in mice significantly lowers the total cholesterol levels in serum and improves the lipid profile of both serum and liver in the hypercholesterolemic subjects as compared to controls. Analysis of microarray data using PANTHER pathway analysis tool, revealed cholesterol metabolic pathway to be among top significantly enriched pathways. We have validated regulation of cholesterol biosynthesis and catabolism by miR‐128 through targeting of HMGCR and CYP7A1 respectively. Significant changes in histopathological analysis by Oil red O and H&E staining of liver tissue is also evident after anti‐miR‐128 treatment in mice. Alteration of miR‐128 levels in mice by anti‐miR treatment could bring down the serum total cholesterol levels in hypercholesterolemic conditions. We report that the altered networks are that of cholesterol biosynthesis, cholesterol degradation and fatty acid metabolism. The results elucidate a novel interaction between miR‐128 and bile acid metabolism that can be further intervened for symptomatic reversal of metabolic disorders linked with the state of increased cholesterol in the body.

## Development of benzimidazole based microtubule destabilizers as cancer chemotherapeutics through fragment based drug discovery approach

**A. K. Verma, S. Singh, K. Fatima, P. Yadav, S. Singh, K. Kumar, S. Luqman, D. Chanda & A. S. Negi**

*CSIR‐Central Institute of Medicinal and Aromatics Plants, Near Kukrail Picnic Spot, Lucknow, Uttar Pradesh, India*

*Email:* vermaamit501@gmail.com

Cancer is a public health menace involving abnormal cell growth and a great concern due to its high morbidity and mortality. Cancer is the second largest cause of death globally, and was responsible for 8.8 million deaths in 2015 which is 14.6% of total human deaths. Benzimidazoles are important bioactive molecules possessing various biological activities including cancer. Tubulin is a globular protein produced in all eukaryotic cells. *α*/*β* tubulin dimer polymerizes into microtubule bundles and help in cell multiplication. Antimicrotubule drugs interfere with the normal dynamic equilibrium of microtubules and cause cell death. To synthesize various benzimidazole analogues possessing a 3,4,5‐trimethoxyphenyl antitubulin fragment based on fragment based drug discovery approach and their anticancer profile. 3,4,5‐trimethoxybenzaldehyde condensed with substituted *o*‐phenylenediamine in presence of lewis acids to give a 2‐aryl benzimidazoles. Further 2‐aryl benzimidazoles converted into desired prototype for antiestrogenic effect via different reactions. A number of molecules were prepared and evaluated for biological activity. Among them compound AB20 and ABN19 showed potent cytotoxicity, IC50 = 6.40 *μ*mol/L and 10.19 *μ*mol/L in MDAMB‐231 cell line respectively, while, ABVA2 and AB14 IC50 = 5.53 *μ*mol/L and 10.90 *μ*mol/L in MCF‐7 cell line respectively. Detailed biological evaluation was carried out on AB20 and ABVA2 compound. Both the benzimidazole analogues were found to be microtubule destabilizers and induced G2/M phase arrest in cell cycle. AB 20 was well tolerable in Swiss albino mice up to 1,000 mg/kg dose in acute oral toxicity. Microtubules are suitable targets in cancer chemotherapeutics. The 3,4,5‐trimethoxyphenyl fragment present in AB20 plays a crucial role in interacting with tubulin and induce a antitubulin effect. These diarylbenzimidazole analogues with antitubulin fragment induce significant antitubulin effect. The potential analogues AB20 and ABVA2 are potential antiproliferative New Chemical Entities (NCEs). These can further be optimized for better efficacy.

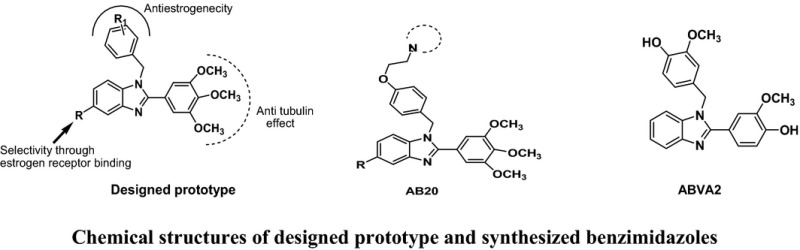

## A case of cutaneous hemangiosaroma in dog

**A. N. Tiwari, G. Maneesh & S. Vikash**

*Vetlab (Veterinary Diagnostic lab) Gomtinagar, Lucknow, Uttar Pradesh, India*

*Email:* anjaniamrendertiwari@gmail.com

Hemangiosarcoma most commonly presents as a multicentric disease involving the spleen, liver, lungs, and right auricle of dogs, especially the German shepherd and golden retriever breeds. Cutaneous involvement can be solitary or, rarely, part of the multicentric syndrome. A 11 year female dog of german shepherd breed had a deep subcutaneous round firm mass which were infiltrating deep in muscle. The tumorous mass was surgically removed. Grossly, it was brownish, nonulcerated and had a soft consistency. Histopathological examination of H & E stained slides revealed presence of areas showing numerous blood vessels lined by neoplastic cells. These neoplastic cells were also forming solid areas. The neoplastic cells were characterized by presence of moderate to marked nuclear and cellular pleomorphism, marked nuclear hyperchromasia and high nucleo‐cytoplasmic ratio. Thus, the dog was diagnosed with hemangiosarcoma.

## Applying synthetic lethality to nanomedicine: LCS‐1 loaded magnetite and polymeric nanoparticles for the treatment of BLM and CHEK2‐deficient colorectal cancer cells

**A. Ahmad, A. Gupta, G. Jayamurugan & R. Khan**

*Institute of Nano Science and Technology (INST), Habitat Centre, Mohali, Punjab, India*

*Email:* anasahmad78689@gmail.com

Synthetic lethality is a pragmatic approach for selectively targeting cancer cells while sparing the healthy cells. It is employed for targeting synthetic lethal interaction of SOD1 inhibition with CHEK2 and BLM defects in somatically mutated colorectal cancer (CRC) cells. LCS‐1 is used for SOD1 inhibition for selective killing of CHEK2 and BLM deficient colorectal cancer cells. To overcome the limitations associated with low aqueous solubility of LCS‐1, magnetic and polymeric nanocarriers (NC) containing LCS‐1 were formulated and characterized. These nanocarriers were aimed to enhance the selectivity and efficacy of LCS‐1 towards gene deficient cancer cells while leaving the non‐cancerous cells unharmed. The nanoparticles were synthesized by nanoprecipitation and solvent evaporation technique and characterized for their size, zeta potential, and poly dispersity index by Dynamic Light Scattering (DLS; Malvern zeta sizer); for shape and surface morphology by Transmission Electron Microscopy (TEM), Field‐Emission Scanning Electron Microscopy (FE‐SEM) and Atomic Force Microscopy (AFM); for drug‐polymer and polymer‐polymer interaction by Fourier Transform Infra‐red Spectroscopy (FT‐IR) and X‐ray Diffraction (XRD) techniques. The biocompatibility and efficacy studies of these nanoparticles were assessed in HEK‐293 and HCT116 colorectal cancer cell lines respectively by MTT assay. In terms of size and zeta potential, the magnetic nanocarriers and polymeric nanoparticles were of 138.3 ± 48.21 nm and 238.7 ± 64.60 nm respectively and showed a zeta potential of +18.3 mV and +21.0 mV respectively. These nanoparticles were coated with biocompatible polymers, hence did not show any toxicity towards normal non‐cancerous cells. LCS‐1 loaded nanoparticles demonstrated high selectivity as compared to free LCS‐1 towards CHEK2 and BLM‐deficient HCT116 cells. LCS‐1 loaded nanocarriers induced persistent DNA damage and apoptosis which demonstrated that LCS‐1‐NC effectively and preferentially killed CHEK2 and BLM‐deficient CRC cells. The nanoparticles exhibited effective uptake in HCT116 colorectal cancer cell as shown by fluorescence and confocal microscopy. Magnetic and polymeric nanoparticles for the effective delivery of LCS‐1 were synthesized and characterized for selective targeting of colorectal cancer cells. These nanoparticles were safe and effective as demonstrated by in‐vitro studies. Further studies in animal model are warranted for establishing their pre‐clinical efficacy.

## Role of TNF‐*α* in cancerous cell (MDA‐MB‐231) and effect of *Azadirachta indica* and alkaline pH

**A. Misra & A. Trivedi**

*Department of Biochemistry, Era's Lucknow Medical College & Hospital, Sarfarazganj, Hardoi Road, Lucknow, UP, India*

*E mail:* indiananchaltrivedi@gmail.com

Cancer is a major public health problem across the world and is the second leading cause of death in the United States. As per Indian Council of Medical Research 2016 report, the total number of cancer cases is expected to reach nearly 17.3 lakh in 2020. Chemotherapy, radiotherapy, surgery are methods to treat cancer but some side effects on normal cells have been found. In our experiments, cells grown in different pH *viz* 7.4, 7.7, 8.0, 8.3, 8.6. containing growth medium were treated with Neem (*Azadirachta indica*) extract. Different concentrations 400, 600, 800 and 1,600 *μ*g/mL of extract were chosen for the treatment. After 48 h supernatant was collected and stored in −80°C deep freezer for determination of TNF‐*α*. Cell viability analysis was done with cytotoxicity assays, trypan blue dye exclusion assay and cytometric analysis. Commercially available Diaclone human TNF‐*α* ELISA kit was used for TNF‐*α* cytokine measurement. It was found that at pH 8.6 and 1,600 *μ*g/mL of *A. indica* extract caused significant mortality (84%) in the MDA‐MB‐231 cell line. These observations suggest that at alkaline pH neem extract is cytotoxic to cancer cell line as well as caused decreased secretion of TNF‐*α*. Today phytochemical based drugs are used and showed promising significance for the treatment of various cancer.

## Reciprocal repression between microRNA‐338‐5p/421 and oncogenic long non‐coding RNA *MALAT1* in SPINK1 positive Prostate cancer

**A. Yadav^1^, V. Bhatia^1^, R. Tiwari^1^, S. Nigam^1^, A. Goel^2^ & B. Ateeq^1^**

*^1^Molecular Oncology Lab, Department of Biological Sciences and Bioengineering, Indian Institute of Technology, Kanpur, Uttar Pradesh, India; ^2^Department of Urology, King George's Medical University, Lucknow, Uttar Pradesh, India*

*Email:* anjaliy@iitk.ac.in

Prostate cancer (PCa) is one of the leading cause of cancer‐associated death among men worldwide. Understanding the mechanism underpinning the disease pathogenesis is critical for developing novel therapeutic strategies for this disease. Multiple lines of evidence suggest that non‐coding RNAs (ncRNAs), including microRNAs (miRNAs) and long non‐coding RNAs (lncRNAs) are implicated in PCa development and progression. Till date, great efforts have been put forth to characterize the role of miRNAs in regulating the expression of coding transcripts. However, little is known about the influence of miRNAs on the expression of non‐coding transcripts such as lncRNAs. Thus, elucidating the role of miRNAs in modulating the expression of lncRNAs in PCa is crucial in order to understand their functional significance and potential utility for therapeutic interventions. Microarray was performed to explore the mRNA/lncRNA expression in miR‐338‐5p/miR‐421 overexpressing 22RV1 cells. Bioinformatics approach was used to verify the target lncRNA and the interaction was validated by luciferase assay. RNA‐immunoprecipitation (RNA‐IP) assay was employed to confirm the interaction between *MALAT1* and EZH2. Previously, we demonstrated that miR‐338‐5p and miR‐421 exhibit tumor suppressive properties in SPINK1 positive prostate cancer (unpublished data). Further, to gain insights into the biological processes altered by miR‐338‐5p and miR‐421, we performed global gene expression profiling of 22RV1 cells overexpressing these miRNAs. Our analysis revealed several lncRNAs deregulated by these miRNAs. One of the lncRNA found to be down‐regulated upon over‐expression of miR‐338‐5p/miR‐421 was an oncogenic lncRNA, *Metastasis Associated Lung Adenocarcinoma Transcript 1* (*MALAT1*). Moreover, *in‐silico* miRNA prediction tools indicated putative binding sites of miR‐338‐5p and miR‐421 on the 3’ end of *MALAT1,* which was further confirmed by luciferase‐reporter assay, indicating miRNA‐mediated post‐transcriptional regulation. Further, we demonstrated that Polycomb group protein EZH2 mediates epigenetic silencing by establishing histone H3K27me3 repressive marks and 5’ methyl cytosine (5mC) marks on the promoters of miR‐338‐5p and miR‐421. Since, *MALAT1* is known to interact with EZH2 and facilitate its recruitment on its target genes, RNA‐IP assay was performed in 22RV1 cells and we observed ˜22‐folds enrichment of *MALAT1* with EZH2 antibody as compared to IgG control. We elucidated a novel reciprocal regulatory network between miR‐338‐5p/miR‐421 and *MALAT1* in SPINK1 positive prostate cancer and a potential therapeutic modality.

## Role of novel microRNAs in non‑small cell lung cancer progression using lung tissues

**A. Singh^1^, M. Ali^2^, V. Prakash^1^, R. Kant^3^ & S. K. Singh^2^**

*^1^Department of Pulmonary and Critical Care, King George's Medical University, Lucknow, Uttar Pradesh, India; ^2^Stem Cell/Cell Culture lab, Centre for Advance Research, King George's Medical University, Lucknow, Uttar Pradesh, India; ^3^Director & CEO, All India Institute of Medical Sciences, Rishikesh, India*

*Email:* singhanjana61@gmail.com

Lung cancer is the leading cause of cancer‐associated mortality worldwide and the majority of bronchogenic carcinomas are cases of non‐small cell lung cancer (NSCLC). NSCLC is often diagnosed at the advanced stages of disease progression, resulting in a poor prognosis and an overall five years survival rate of ˜14%. However, the five‐year survival rate in patients with stage I NSCLC that has been resected can be as high as 83%. miRNAs are a class of short RNAs that are 19–25 nucleotides in length and regulate gene expression by binding to sequences in the untranslated region (UTR) of an expressed mRNA. miRNAs have been associated with cell signalling pathways involved in cell differentiation, proliferation and survival. The aberrant expression of miRNAs has been demonstrated in a number of different types of tumour including lung cancer. Tissue biopsies from lungs have been snap freeze in liquid nitrogen and 3 mL of peripheral blood sample were collected in EDTA vials. All the samples were stored in liquid nitrogen from both patients and controls. All the subjects have been analyzed for expression of miRNA by RT‐PCR in lung tissue. The expression pattern was analyzed in serum to establish it as a noninvasive biomarker for lung cancer diagnosis. miRNA was extracted from both serum as well as lung tissue. Expression profile of hsa‐mir‐449c, hsa‐mir‐2114, hsa‐mir‐2115 suggested significant association. The role significantly deregulated miRNA in cellular processes (like proliferation, differentiation, apoptosis etc) and its mechanism of action is to be determined.

## Gallic acid based indanone derivatives as anticancer agents through modulation of microtubule dynamics

**A. Srivastava^1^, K. Fatima^1^, T. Kaushal^1^, M. Hasnain^2^, M. Maheshwari^2^, H. Iqbal^1^, J. Sarkar^2^, S. Luqman^1^, D. Chanda^1^, K. Shanker^1^ & A. S. Negi^1^**

*^1^CSIR‐ Central Institute of Medicinal and Aromatic Plants, Lucknow, Uttar Pradesh, India; ^2^CSIR‐ Central Drug Research Institute, Lucknow, Uttar Pradesh, India*

*Email:* ankitasri2020@gmail.com

Cancer is a group of diseases involving abnormal cell growth. Cancer is the second leading cause of death globally, and was responsible for 8.8 million deaths in 2015 which is 14.6% of total human deaths. Antitubulin is one of the most successful approaches in cancer drug development. Applying fragment based drug discovery to synthesize various indanone related analogues possessing trimethoxyphenyl fragment as antitubulin cancer chemotherapeutics. Modification of gallic acid to suitable substrate, Claisen–Schmidt reaction to get hexamethoxychalcones, cyclization through Nazarov's reaction, SeO2 oxidation, detailed cancer pharmacology. Among various indanone based analogues, ANK‐2HINDP and NNDI exhibited potent anti‐proliferative activity, IC50 = 2.22 *μ*mol/L and 1.23 *μ*mol/L, respectively, against colorectal adenocarcinoma (DLD1) cell line. Both the compounds showed antitubulin effect in tubulin kinetics and in confocal microscopy. In cell cycle analysis, these analogues exerted G2/M phase arrest. Both ANK‐2HINDP and NNDI induced apoptosis through caspase pathway by cleaving PARP. Both the indanone derivatives were well tolerated up to 1,000 mg/kg dose by Swiss albino mice in acute oral toxicity experiments. Microtubules are considered as an attractive target in cancer chemotherapeutics. 3,4,5‐Trimethoxyphenyl unit in both ANK‐2HINDP and NNDI is very crucial to interact with tubulin and induce antitubulin effect. Apoptosis induction may be due antitubulin effect and caspase activation. Both ANK‐2HINDP and NNDI exhibited potential anticancer activity through antitubulin effect and by induction of caspase pathway. Both the indanone derivatives can further be optimized for better efficacy.

## Correlation of *AGTR1*(A1166C) and *ACE*(I/D) polymorphisms with breast cancer risk in North India

**A. Singh & B. Ateeq**

*Molecular Oncology Lab, Department of Biological Sciences and Bioengineering, Indian Institute of Technology, Kanpur, Uttar Pradesh, India*

*Email:* anukriti@iitk.ac.in

Renin angiotensin system (RAS) comprising Angiotensin II receptor type I (AGTR1), a receptor of Angiotensin II (Ang II) and Angiotensin converting enzyme (ACE), plays a critical role in many diseases including cancer. An insertion/deletion (I/D) polymorphism in intron 16 of *ACE* and a single nucleotide polymorphism (SNP) A1166C found in 3'untranslated region (UTR) of *AGTR1* are reported to be implicated in various diseases; however their association with Breast Cancer (BCa) is still a topic of debate. This is the first study to explore the association of these polymorphisms in a North Indian BCa cohort including 161 patients and 152 healthy women. The polymorphisms were assessed by polymerase chain reaction (PCR) and restriction fragment length polymorphism (RFLP) respectively. The association between these polymorphisms and BCa risk was estimated by calculating Chi‐Square (*χ* ^2^ test) and Odds Ratio (OR). The “AC and CC” genotype/ C allele of *AGTR1* (A1166C) polymorphism and DD genotype/D allele of *ACE* (I/D) polymorphism were correlated with higher risk of BCa when assessed independently. However, on interaction analysis of “AC and CC” and DD genotype and combination of “C and D” alleles of both polymorphisms, a significantly greater risk of BCa was observed. Both *ACE* (I/D) and *AGTR1* (A1166C) polymorphisms were independently associated with BCa risk, however together these polymorphisms confer a far greater risk of BCa in women harbouring them. Women harbouring DD genotype/D allele for *ACE* (I/D) polymorphism and “AC or CC” genotype /C allele for *AGTR1* (A1166C) polymorphism are at a risk of more aggressive form of the disease with advanced staging and larger tumor size. Our study brings forward the importance of developing a genetic screen based on these polymorphisms for women who might be susceptible to higher BCa risk.

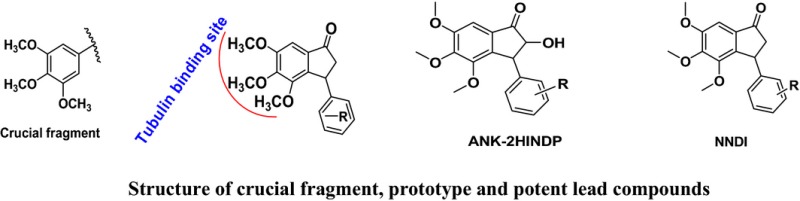

## Cardiovascular complications in patients with sickle cell disease

**A. Chaturvedi**

*Experimental and Public Health Laboratory, Department of Zoology, University of Lucknow, Lucknow, Uttar Pradesh, India*

*Email:* ashishchaturvedi.jr.sc@gmail.com

Sickle cell disease (SCD) is an autosomal recessive disease in which homozygosity for a single point mutation in the gene encoding the *β*‐globin chain produces hemoglobin S molecules that polymerize within the erythrocyte during deoxygenation; the result is sustained hemolytic anemia and vaso‐occlusive events. As patients live to adulthood, the chronic impact of sustained hemolytic anemia and episodic vaso‐occlusive episodes leads to progressive end‐organ complications. This scenario culminates in the development of 1 or more major cardiovascular complications of SCD for which there are no approved or consensus therapies. These complications include elevated pulmonary artery systolic pressure, pulmonary hypertension, left ventricular diastolic heart disease, dysrhythmia, sudden death, and chronic kidney disease with associated proteinuria, microalbuminuria, and hemoglobinuria. In patients with advancing age, cardiopulmonary organ dysfunction and chronic kidney injury have significant effects on morbidity and premature mortality. Over the last 15 years, a number of tests have been validated in multiple replicate cohort studies that identify patients with SCD at the highest risk of experiencing pulmonary and systemic vasculopathy and death, providing for screening strategies tied to targeted, more aggressive diagnostic and therapeutic interventions.

## *α*‐Mangostin inhibits the growth and induces apoptosis of human ovarian carcinoma, PA‐1 cells through cell cycle arrest, ROS generation and Caspase‐3 activation

**A. Jafri, J. Rais, S. Siddiqui, S. Kumar & M. Arshad**

*Molecular Endocrinology Lab, Department of Zoology, University of Lucknow, Lucknow, Uttar Pradesh, India*

*Email:* asifjafri.jafri@rediffmail.com

Ovarian cancer is the seventh most common cancer among women in the world and accounts for 152,000 deaths worldwide annually. Therefore, the search of promising apoptotic agents that decline ovarian carcinoma is a very significant task. Some potent phytochemicals have shown a new light in the development of promising comprehensive cancer chemopreventive agents that could induce apoptosis without affecting the normal cells. *α*‐Mangostin (AM), a natural xanthonoid, isolated from the mangosteen tree (*Garcinia mangostana*) and possesses versatile pharmacological activities. The aim of present study is to observe the anti‐proliferative and apoptotic activity of AM against ovarian carcinoma, PA‐1 cells. We examined the cytotoxicity and growth inhibition of PA‐1 cells after AM treatment by potent biomarkers *viz*. MTT assay, analysis of morphological alteration, nuclear condensation assay, reactive oxygen species (ROS) generation study and analysis of mitochondrial membrane potential (MMP). Furthermore DNA fragmentation, cell cycle progression and caspase‐3 activity analysis were also carried out. The findings revealed that AM significantly suppresses the proliferation of PA‐1 cells with an associated augmentation in nuclear condensation in a dose dependent manner. The significant increment in intracellular ROS production, DNA fragmentation and induction of mitochondrial membrane depolarization supports AM mediated apoptosis of ovarian carcinoma. The cell cycle study exposes that AM arrest cell cycle in G0/G1 phase and caspase‐3 activity confirmed apoptosis of PA‐1 cells. Present study confirms the anti‐proliferative efficacy of AM that lead to cellular apoptosis in human ovarian carcinoma cells *via*. decrease in cell viability, intracellular ROS production, mitochondrial membrane depolarization, nuclear condensation, cell cycle arrest and caspase‐3 activity. In conclusion, the findings of present study indicate that AM encourages cell death and lowers the growth of PA‐1 cells in concentration‐dependant manner. Further more work is needed to determine its potential as a chemopreventive and/or chemotherapeutic agent.

## Epigenetic modulator EZH2 governs CSC properties and alters metastatic cascade

**A. verma, A. singh, A. K. Singh, P. chaturvedi, M. A. Nengroo & D. Datta**

*Biochemistry Division, CSIR‐Central Drug Research Institute, Lucknow, India*

*Email:* ayushicdri@gmail.com

Drug resistance, disease relapse and metastasis are the major culprits for patient mortality that are known to be caused by ‘few smartly evolved cells’ or Cancer Stem Cells (CSCs) within solid tumors. Latest evidence suggests that diverse epigenetic alterations, including Polycomb Group (PcG) of proteins‐mediated dynamic histone methylation are driving force for CSCs to pose its deleterious effects. Enhancer of Zeste homologue 2 (EZH2) is the enzymatic subunit of Polycomb repressive complex 2 (PRC2) which methylates lysine 27 of histone H3 (H3K27) and classically function as a transcriptional repressor. Besides its trimethylation function, EZH2 protein has recently been shown to play pivotal role in tumorigenic process. Here, we aim to dissect the differential role EZH2 (EZH2 protein versus H3K27me3) in modulating CSC properties *in‐vitro* and *in‐vivo*. Stable cancer cells for differential EZH2 expression were achieved by retroviral transduction of particular plasmids (wild type, knockdown and EZH2 mutant constructs). Spheroid formation, aldefluor, colony formation, and SRB assays were used to determine CSC properties. Expression analyses were done by performing Real‐Time PCR, Western Blot, and Flow cytometry. *In‐vivo* tumorigenic potential was determined by different subcutaneous xenograft tumor models in nude mice. By utilizing differentially modified EZH2 Luc and EZH2 GFP (green) and EZH2 Td‐Tomato (red) cell based orthotopic tumor models, we visualized metastatic progression of breast cancer in live animals. EZH2 knockdown in colon (DLD‐1, HT‐29) and breast (HCC‐1806, 4T‐1) cancer cells result in compromised CSC properties *in‐vitro* (spheroid formation and drug resistance) and *in‐vivo* (tumorigenic potential) as compared to control cells. Interestingly, compared to control breast cancer cells, selective induction of H3K27me3 but not EZH2 promotes robust spheroid formation and drug resistance against Paclitaxel. Surprisingly, the functional EZH2 activation (H3k27Me3 overexpression) and EZh2 protein (over expression) cause significant small tumor as compared to control. Though having smaller primary tumors, functional activation of EZH2 results in early metastatic invasion and most importantly; it alters the pattern of breast cancer metastasis as observed by sequential live animal imaging experiments. Targeting functional activation (H3K27me3) of EZH2 could be a wonderful strategy to mitigate drug resistance and cancer metastasis.

## C5 derived esters of brevifoliol as anticancer agents against prostatic adenocarcinoma

**B. Bhukya^1^, K. Fatima^1^, R. Tyagi^1^, V. Gupta^1^, A. Nagar^1^, S. Kumar^2^, S. Luqman^1^, S. Tandon^1^, K. Shanker^1^ & A. S. Negi^1^**

*^1^CSIR‐Central Institute of Medicinal and Aromatics Plants, Lucknow, Uttar Pradesh, India; ^2^Department of Applied Chemistry, Babasaheb Bhimrao Ambedkar University, Lucknow, Uttar Pradesh, India*

*Email:* balakishan333@gmail.com

*Taxus wallichiana* known as Himalayan yew, belongs to the family Taxaceae. It has been used by the native populations for treating common cold, cough, fever and pain. Its uses are described in Ayurveda and Unani medicine. It is prime source of paclitaxel and brevifoliol. Brevifoliol is a rearranged taxoid known as abeotaxane. The study aims at semi‐synthetic modification of brevifoliol at C5 positions for enhanced anticancer activity. Isolation of brevifoliol from ethyl acetate extract of *Taxus* needles and its purification was done through column chromatography. Semi‐synthetic derivatives were prepared using esterification method at C5 position and evaluated for biological activity against various human cancer cell lines. Two of the semi‐synthetic analogues i.e. **BRE‐9R** and **BRE‐16** exhibited potent cytotoxicity against PC‐3, prostate cancer cell lines, IC50 4.9 *μ*mol/L and 3.9 *μ*mol/L respectively. In cell cycle analysis **BRE‐16** induced G2/M phase arrest. **BRE‐16** showed safety index of 29 as compared to healthy cell lines. Brevifoliol is a rearranged taxoid, which is in much higher quantity in Himalayan Yew as compared to paclitaxel. Brevifoliol has previously exhibited moderate cytotoxicity against several human cancer cell lines. Like paclitaxel, **BRE‐16** may also affect microtubule dynamics to induce apoptosis in cancer cells. Semi‐synthetic analogues **BRE‐16** and **BRE‐9R** are potential lead compounds. However, their detailed mechanistic study needs to be determined. The study reveals that C5 positions of brevifoliol are very much prone to favourable modifications.

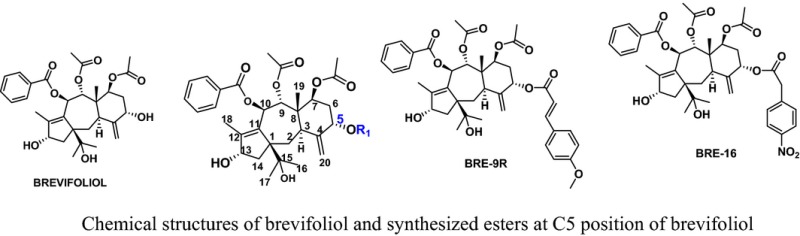

## Berberine induces toxicity in HeLa cells through perturbation of microtubule polymerization by binding to tubulin at a unique site

**D. Raghav, S. M. Ashraf & K. Rathinasamy**

*School of Biotechnology, National Institute of Technology Calicut, Calicut, Kerala, India*

*Email:* darpan1991_raghav@yahoo.com

Berberine, an isoquinoline alkaloid has been used traditionally for its diverse pharmacological actions. It has shown to exert significant anticancer activity. Berberine is reported to induce apoptosis in several cancer cell lines such as T47D, MCF‐7, HTB‐94 and PC3 by inducing G2/M phase cell cycle arrest, however the underlying mechanism remains largely unknown. The present study aims to decipher the cytotoxic mechanism of berberine. The anti‐proliferative activity of berberine on human cervical cancer (HeLa) cells was determined by using sulforhodamine B assay. Flow cytometric analysis was performed to monitor the cell cycle progression of HeLa cells upon berberine treatment. Effect of berberine on interphase and mitotic spindles of HeLa cells was determined by fluorescent microscopy. The interactions between berberine and tubulin were characterized using fluorescence spectroscopy. The effects of berberine on microtubule polymerization were studied using light scattering assay. The binding site of berberine on tubulin was characterized by computational docking, site‐maker competition experiments and by fluorescence resonance energy transfer (FRET) analysis. The conformational changes in tubulin secondary structure upon berberine binding were analysed using Fourier transform infrared spectroscopy (FTIR). Berberine inhibited the proliferation of HeLa cells with an IC50 of 18 *μ*mol/L. At its IC50, berberine exerted a moderate G2/M arrest and induced significant depolymerization of interphase and mitotic microtubules. In‐vitro studies with tubulin isolated from goat brains indicated that berberine bound to tubulin with a *K*d of 11 *μ*mol/L at a novel site 24 Å away from the colchicine binding site and inhibited the polymerization of tubulin into microtubules. Data obtained from the FTIR studies revealed that binding of berberine altered the conformation of the tubulin heterodimer. The antiproliferative activity of berberine correlated well with its ability to depolymerize the interphase and mitotic spindles. Also, altering the conformation of the tubulin heterodimer could be the molecular mechanism behind the depolymerizing effects on berberine on tubulin assembly. Our results indicate that binding to tubulin and perturbation of microtubule polymerization could be one of the possible mechanisms behind the cytotoxic activity of berberine.

## SKI‐II modulates gamma ray induced toxicity by activating Nrf2 and suppressing oxidative stress in hematopoietic cells

**D. K. Sah^1,2^, Y. Rai^1^, N. kumari^1^, M. M. Chaturvedi^2^ & A. N. Bhatt^1^**

*^1^Institute of Nuclear Medicine and Allied Sciences, DRDO, Delhi^2^Department of Zoology, University of Delhi, Delhi, India*

*Email:* sahasan_99@yahoo.com

Acute hematopoietic syndrome is a characteristic of Gamma ray (ionizing radiation, IR) exposure, inducing cell apoptosis by eliciting oxidative stress and inflammation. Hematopoietic cells are highly proliferative and more prone to IR induced apoptosis and cell death. In unstressed cells, Nrf2 is sequestered in the cytoplasm by Keap1 promoting its rapid proteasomal degradation. SKI‐II (2‐(phydroxyanilino)‐4‐(p‐chlorophenyl) thiazole) deactivate Keap‐1 molecule by inducing dimer formation between two keep‐1 molecules, which in turn unable to bind with Nrf2, and make Nrf2 free. Increased free Nrf2 molecule translocates to the nucleus and in turn activate the antioxidant defence. In this study, we demonstrated that treatment with SKI‐II protects radiosensitive hematopoietic cells (THP‐1, Raw 264.7) from radiation induced cell death. We further unrevealed the underlying mechanisms involved in the protective effects of SKI‐II in IR‐induced cytotoxicity using hematopoietic cells. We observed that SKI‐II treatment inhibits IR‐induced apoptosis by attenuating reactive oxygen species production. SKI‐II pre‐treatment results in 60% decrease in radiation induced total ROS level measured by fluorescence of 2,7‐dichlorofluorescein (DCF) which is a hydrolyzed product of H2DCF‐DA at early and late time points after exposure. It also reduces radiation induced mitochondrial ROS by 20%. The decrease in radiation induced ROS in SKI‐II treated cells correlates with more than 50% increase in the level of reduced glutathione at 2, 4 and 14 h respectively as compared to IR exposed group. Enhanced antioxidant defense potential of SKI‐II treated cells leads to decrease in radiation induced cell death. Cell membrane integrity was monitored with PI uptake assay on flow cytometry; we observed nearly 70% and 24% reduced PI uptake in THP‐1 and Raw264.7 cells, respectively. This observation correlates with reduced radiation induced lipid peroxidation in SKI‐II treated cells. SKI‐II treatment also recovers from the radiation induced disturbance in mitochondrial membrane potential which correlates with reduced cell death monitored using EtBr/ AO staining. Therefore, SKI‐II treatment reduces radiation induced toxicity by activating Nrf2 mediated anti‐oxidant defense system in hematopoietic cells.

## Natural product inspired development of cancer chemotherapeutics: oleanolic acid derivatives as anticancer agents

**D. S. Raghuvanshi^1^, N. Verma^1^, S. Singh^2^ & S. Luqman^2^**

*^1^Department of Medicinal Chemistry, CSIR‐Central Institute of Medicinal and Aromatic Plants, Lucknow, Uttar Pradesh, India^2^Molecular Bioprospection Department, CSIR‐Central Institute of Medicinal and Aromatic Plants, Lucknow, Uttar Pradesh, India*

*Email:* dushyant.bhu@gmail.com

In recent years, interest in synthetic transformations of natural products for the purpose of preclinical development is a major objective of antitumor research programs. Pentacyclic triterpenoids are secondary plant metabolites found in fruit peel, leaves, stem bark and roots. Their potential interest in cancer has now been well demonstrated by the successful clinical utilities of oleanolic acid (OA), glycyrrhetic acid, asiaticoside, and carbenoxolone as marketed drugs. 4*H*‐chromene derivative Crolibulin (EPC2407) is currently in phase I/II clinical trials for the treatment of advanced solid tumor. The present study aims to prepare crolibulin based template of pentacyclic triterpene as effective and safer anticancer drugs. Oleanolic acid, a pentacyclic triterpene, isolated from the roots of *Lantana camara*, transformed to bioactive intermediates *α*,* β*‐unsaturated compounds, and chromenes through a green process, biological evaluation against various human cancer cell lines and detailed pharmacology. Antiproliferative effect of *α*,*β*‐unsaturated and chromene series were analyzed using MTT assay in a panel of five cancer cell lines prevalent in the clinics. In the series of *α*,*β*‐unsaturated compounds, two compounds showed selectivity for breast cancer cells with IC50 31.10 *μ*g/mL and 39.04 *μ*g/mL respectively while rest of the molecules of this series showed significant proliferation inhibition. Further, two molecules of chromene derivatives inhibited the growth of lung and breast cancer cells with IC50 12.23–17.46 *μ*g/mL. The identified leads are under evaluation for their mode of action. In conclusion, four oleanolic acid derivatives were identified as potentially antiproliferative active in A549 and MDAMB‐231 cells which may be used as templates for the future drug developments.

## Chemopreventive potential of carvacrol against androgen‐dependent prostate cancer cell line via induction of apoptosis and cell cycle arrest

**F. Khan, I. Khan & I. A. Ansari**

*Department of Biosciences, Integral University, Lucknow, Uttar Pradesh, India*

*Email:* fahadintegralian@gmail.com

Prostate cancer represents the second most prevalent cancer in males worldwide and is a major cause of morbidity and mortality. Although there are several therapeutic options, a safer and more effective modality is urgently needed for treatment of prostate cancer. In this regard, carvacrol, a monoterpene phenol from plants of *Lamiaceae* family has gained considerable attention recently because of its potential chemopreventive properties. The aim of this study was to investigate the antiproliferative and anticancer potential of carvacrol on androgen‐dependent human prostate cancer cell line (LNCaP). The MTT and trypan blue dye exclusion assay were used to evaluate the cytotoxicity of carvacrol on LNCaP cell line. Furthermore, cell cycle analysis, apoptosis assay, and real time PCR were performed to explore the possible mechanisms of action. Results of the present study indicated that carvacrol inhibited the growth of prostate carcinoma LNCaP cell line in a dose and time‐dependent manner. We also demonstrated that carvacrol significantly induced apoptosis in prostate cancer cells via mitochondrial apoptotic pathway as a result of increased reactive oxygen species (ROS) production nuclear fragmentation and decreased level of mitochondrial membrane potential. We observed that carvacrol treatment also caused a significant cell cycle arrest in LNCaP cells at G0/G1 phase in a dose and time‐dependent manner. Moreover, real time PCR analysis also revealed that carvacrol increased the gene expression of proapoptotic proteins such as Bax and Bid and decreased the expression of antiapoptotic proteins such as Bcl‐2. Taken together, these results indicated that carvacrol inhibited the growth of LNCaP prostate cancer cell by inducing apoptosis and cell cycle arrest. These results support further investigation of carvacrol as a potential therapeutic agent in the treatment of prostate cancer.

## Inhibition of p38MAPK pathway and induction of mitochondria‐mediated apoptosis in human prostate cancer cells by a synthetic sulfonylhydrazone derivative

**G. P. Sharma^1^, L. Nigam^2^, A. Inam^3^, S. Naidu^2^, A. Azam^3^ & N. Mondal^1^**

*^1^School of Life Sciences, Jawaharlal Nehru University, New Delhi, India; ^2^School of Computational and Integrative Sciences, Jawaharlal Nehru University, New Delhi, India; ^3^Department of Chemistry, Jamia Millia Islamia, New Delhi, India*

*Email:* gurusbtjnu@gmail.com

Prostate cancer is one of the leading causes of cancer‐related death in men globally. One of the main reasons for the systemic toxicity of the available anticancer drugs is the use of very high dose. Sulphonyl hydrazones and their analogs constitute a class of cancer chemotherapeutic agents. However, their broad biological characterization has not been explored yet. *In‐silico* study to screen a potent drug target for and candidate from different sulfonylhydrazone conformers available at Pub Chem database. Further, subjecting the selected sulfonylhydrazone derivative to the biological characterization on metastatic prostate cancer cells. *In‐silico* work involved analysis of target protein status across the patients using www.cbioportal.org and canSAR database. Molecular docking analysis to show the interaction between protein isoforms and compound. Further, MTT assay, FACS, western blotting, Kinase assay, and immunofluorescence studies were done using DU145, PC‐3 cell lines. *In‐silico* data showed that p38*α* (MAPK14) is overexpressed in many prostate cancer and its “DFG” site is potentially targeted by compound SH‐1(PubChem CID: 6033590). SH‐1 indicated IC50 of 60 *μ*mol/L and 70 *μ*mol/L in DU145 and PC‐3 respectively. Wet‐experiment data showed that SH‐1 inhibited p38MAPK pathway, arrested cells in S‐phase and inhibited cyclin A‐associated CDK2 activity. SH‐1 induced nuclear expression of BRCA1, and p21. SH‐1 induced the depletion of mitochondrial membrane potential (MMP), cleavage of PARP and caspase‐3, and downregulation of Bcl‐2 and survivin. Subcytotoxic concentration of SH‐1 (10 *μ*mol/L) was used in the whole study with an aim to minimize systemic toxicity. The p38MAPK pathway is activated by various cellular stresses and promote cell proliferation and survival in many types of cancer. SH‐1 interacted and inhibited p38MAPK pathway leading to an inhibition of the cell proliferation. The nuclear expression of BRCA1 and p21 promotes apoptosis, and that was further suggested by SH‐1‐induced inhibition in MMP, cleavage of PARP and caspase‐3 and inhibition of anti‐apoptotic markers bcl‐2 and survivin. Further, the biochemical data clearly indicated insignificant effects on stress markers in the mice. The preferential killing of cancer cells by SH‐1 advocate its potential anticancer property and the possibility of its use in prostate cancer therapy.

## Role of major dietary polyphenols in treatment and chemoprevention of prostate cancer

**H. Khan, A. Jafri, N. Shivanth, V. Rawat, J. Rais, N. Bano & M. Arshad**

*Molecular Endocrinology Lab, Department of Zoology, University of Lucknow, Lucknow, India*

*Email:* habibakhan2305@gmail.com

Prostate cancer is the sixth leading cause of cancer deaths among men worldwide. The prostate gland present in the pelvis region is essential for the proper functioning of the male reproductive system. The Androgen Receptor (AR) is a ligand‐dependent transcription factor. AR binds to its native ligands 5*α*‐dihydrotestosterone (DHT) and testosterone that initiates male sexual development and differentiation. Prostate cancer begins when cells in the prostate gland start to grow uncontrollably which involves multiple factors. Conventional cancer therapies evoke severe side effects and to overcome these problems use of polyphenols is emphasized. The previous study suggests that polyphenols chemopreventive agents found in fruits and vegetables reverse multi‐stages of carcinogenesis and also suppress cancer proliferation through induction and stimulation of cell death. Epigallocatechin‐3‐gallate (EGCG) is present in green tea, chocolates and grapes affect mitogenesis as well as induces apoptosis and cell cycle arrest in prostate cancer cells. Curcumin is a major chemical component of turmeric. *In vitro* studies reveal that upon treating prostate cancer cells with curcumin suppression of Bcl2 and Bcl‐xL occurs which in turn triggers DNA damage and cell death. Resveratrol is found in red grapes, red wine, mulberries and peanuts. Resveratrol causes cell‐cycle arrest and increases apoptotic cell death mediated by activation of caspases‐3 and ‐9 and a change in the ratio of Bax/Bcl2. Capsaicin is found in red and chili peppers. It induces apoptosis by increasing the production of ROS in prostate cancer cells. Quercetin is found in onions, broccoli, apples, grapes and in soybeans and inhibits proliferation of prostate cancer cells by inhibiting the PI3K/Akt pathway, cytochrome‐c release, and consequently apoptotic death. Hesperidin is found in citrus fruits lemons, limes, oranges, and tangerines inhibit cell proliferation of prostate cancer cells. Furthermore, scientific evidence and well‐conducted clinical studies are needed to clarify the efficacy of polyphenols on prevention of Prostate cancer cells.

## Oxidative stress induced by co‐exposure to arsenic and fluoride in Wistar rat

**H. Khan, Y. Verma & S. V. S. Rana**

*Toxicology laboratory, Chowdhary Charan Singh University, Meerut, India*

*Email:* humakhan3092@gmail.com

The concurrent chronic human poisoning with fluoride and arsenic is an emergent environmental health problem in India as well as many other countries including China, Mexico, Argentina and Bangladesh. There are several places throughout the globe where arsenic and fluoride both are present in ground water at high concentration. Present findings will help in the management health problems emerging on co‐exposure to arsenic and fluoride in endemic areas. The experimental data on the concomitant effects of arsenic and fluoride on major organ is wanting. There are no conclusive evidences if the combined exposure to them will lead to an antagonistic or synergistic effect especially in mammals. Till date there is no effective treatment for chronic arsenic or fluoride poisoning therefore, an attempt was made to gather the information regarding the mode of action of these toxicants. These investigations were carried out employing either sex of laboratory rat of Wistar strain. The rats offered drinking water containing sublethal concentration of arsenic (sodium arsenate) and fluoride (sodium fluoride) for a period of 30 to 90 days. Liver and kidney were considered the target organs. However, urine and blood analysis were also be made whenever found necessary. The entire study comprises parameters viz, protein estimation, lipid peroxidation, reduced glutathione, serum calcium, histopathological alteration. Overall results indicated that arsenic and fluoride are less toxic to liver and kidney than their individual exposure. In brief, it is concluded that an antagonistic mechanism in their combined toxicity which relies upon the improved antioxidative status of liver and kidney both. Further studies on antioxidative enzymes are however, needed to confirm antagonistic behaviour between arsenic and fluoride. Arsenic manifestation is causing arseniasis whereas, fluoride toxicity results into fluorosis. Combined treatment of arsenic and fluoride at sublethal doses to rat resulted in lower values for malondialdehyde, a product of lipid peroxidation. A decline in GSH values in liver but increase in kidney showed certain degree of improvement. Serum hypocalcemia also recorded after their combined treatment. Histopathological observations showed limited degree of improvement after their combined treatment.

## Synergistic inhibition of human breast cancer cells by combination of Salinomycin and 2‐Methoxyestradiol *in vitro*

**J. Dewangan, S. Srivastava, P. K. Pandey, S. Mishra, A. Divakar & S. K. Rath**

*Genotoxicity Lab, Division of Toxicology and Experimental Medicine, CSIR‐Central Drug Research Institute, Lucknow, Uttar Pradesh, India*

*Email:* jayantdewangan@gmail.com

2‐Methoxyestradiol (2‐ME) is an endogenous metabolite of estradiol currently under clinical trial for the treatment of advanced solid tumors. Salinomycin is a polyether ionophore antibiotic that selectively targets breast cancer stem cell population and kills various types of tumors. However, the high dose of Salinomycin exhibited some toxic effects. Therefore, identification of drugs having marginal toxic response is required for the treatment of breast cancer and amelioration of adverse events associated with chemotherapy. In the present study, we aimed to evaluate the synergistic inhibition of estrogen‐responsive human breast cancer cells with Salinomycin and 2‐ME co‐treatment. MCF‐7 cells were treated with either alone or in different combinations of Salinomycin and 2‐ME for 24 h. Subsequently, sulforhodamine B (SRB) assay was performed to assess the cytotoxic response. Drug interaction was studied via Chou and Talalay method using CompuSyn software. Cell growth inhibition was recorded by flow cytometer after propidium iodide staining. Furthermore, combination induced apoptosis response was studied by Annexin V and propidium iodide staining and recorded flow cytometrically. Salinomycin and 2‐ME combination significantly inhibited MCF‐7 cell growth. Combination of a lower dose of Salinomycin exerted greater antiproliferative response with 2‐ME. CompuSyn was used to generate combination index (CI) value, 1 *μ*mol/L of Salinomycin with 50 *μ*mol/L 2‐ME generated 0.2 CI value. Similarly, other combination 2.5 *μ*mol/L of Salinomycin and 50 *μ*mol/L 2‐ME gave 0.1 CI valve. CI less than 1 represents synergistic drug combination, however, more than 1 and equal to 1 CI value denote antagonistic and additive effect respectively. CI plot and Isobologram represented the synergistic drug combination. Furthermore, Salinomycin and 2‐ME combination arrested cell cycle at the G2/M phase and significantly increased sub G1 population, indicating apoptosis. The cytotoxic response was confirmed by Annexin V/ PI staining. Co‐treatment elevated Annexin V/PI positive cell population as compared to control and either drug‐treated cell. In conclusion, Salinomycin and 2‐ME was effectively inhibited the growth of MCF‐7 cells. The study suggests that Salinomycin and 2‐ME could offer a novel combination approach for the treatment of breast cancer.

## Biochanin a induces cellular apoptosis and inhibits cell proliferation of human ovarian carcinoma cells, PA‐1

**J. Rais, A. Jafri, M. Tripathi & M. Arshad**

*Molecular Endocrinology Lab, Department of Zoology, University of Lucknow, Lucknow, Uttar Pradesh, India*

*Email:* juhirais44@gmail.com

Ovarian carcinoma has a multifactorial origin. It is extremely aggressive carcinoma, causes highest mortality in females because of its insidious nature, nonspecific symptoms, limited screening tools and poor prognosis in early stages of disease. In recent years, the extensive *in vitro* and *in vivo* studies on phytochemicals have shown a new light as they contain a bioactive compound which has the potential to get developed into a stand‐alone therapeutic regime that enhances the effects of conventional therapy by inhibiting factors that are dysregulated in malignant cells. Biochanin A is an O‐methylated isoflavone, a major isoflavone in red clover and a potent chemopreventive agent against many cancers. This study investigated the anti‐proliferative and apoptotic potentials of Biochanin A on human ovarian carcinoma PA‐1 cells through cell viability assay, loss of mitochondrial membrane potential (MMP), nuclear apoptosis, reactive oxygen species (ROS) generation and cell cycle analysis. The MTT cellular viability assay reveals that Biochanin A significantly induces morphological alterations in PA‐1 cells in a dose dependent manner and thus inhibits cell proliferation. The cytotoxic effect of Biochanin A *via* the induction of apoptosis is evident by the disruption of MMP, nuclear fragmentation, ROS accumulation and cell cycle arrest at G0/G1 phase. In conclusion, our results demonstrated that Biochanin A promotes significant apoptosis in human ovarian carcinoma PA‐1 cells through the mechanisms involving mitochondrial perturbation. Therefore, Biochanin A is a potent candidate that aids for adjuvant cancer treatment; further studies are needed to elucidate the comprehensive mechanistic pathways.

## Neo‐menthol, a cyclic monoterpenoid inhibits the growth of skin cancer cell line by targeting hyaluronidase

**K. Fatima, Z. A. Wani & S. Luqman**

*CSIR‐Central Institute of Medicinal and Aromatic Plants, Lucknow, Uttar Pradesh, India*

*Email:* fakrunnisha786@gmail.com

Cancer is one of the most complicated diseases responsible for human death worldwide. Biomarkers play an important role in carcinogenesis process and their expression level indicate status of the disease, hence targeting biomarker by natural product is one of the adequate approaches for cancer prevention and treatment. Herein, we are reporting antiproliferative effect of neo‐menthol (an isomer of menthol) by targeting hyaluronidase utilizing cell and molecular bioassays. The present study aims to identify monoterpenoids that modulates carcinogenesis process by suppressing the cell and molecular targets and/or biomarkers in different cancer cell lines. The antiproliferative activity of neo‐menthol was tested on various human cancer cell lines by performing MTT, SRB and NRU assays. The effect was also determined on different targets by established *in vitro* cell and molecular target based assays. A binding interaction between the receptor and ligand was also examined using *in silico* approach. Furthermore, the efficacy of the monoterpenoids was confirmed by performing real time expression studies and cell cycle analysis. *In vivo* acute toxicity was also done to find the safety limit of the dose. Finally, the potential was confirmed on mice with Ehrlich Ascites carcinoma. Among the screened monoterpenoids, neo‐menthol exhibited potent anti‐proliferative activity (IC50:17.66 *μ*mol/L) against human epidermoid carcinoma (A431) compared to other cell lines. Among the tested molecular targets, it inhibits hyaluronidase activity (IC50:12.93 *μ*mol/L). Interestingly, real time expression data also validates the *in vitro* cell and molecular target based results. *In silico* analysis also revealed a strong binding interaction (BE −5.96 kcal/mol) of neo‐menthol with hyaluronidase. In cell cycle analysis, it exerts S‐phase arrest followed by decrease in number of sub‐diploid cells. In mice with Ehrlich Ascites carcinoma it showed 60.49% reduction of tumor growth at 75 mg/kg while the animal tolerates up to 1000 mg/kg dose in acute oral toxicity. The three methyl groups of neo‐menthol might be responsible for lipophilicity, while hydroxyl group induces hydrophilicity, follows Lipinski rule. The hydroxyl group might be responsible for the better antiproliferative activity of neo‐menthol in A431 cell line. Neo‐menthol also retarded the growth of skin carcinoma by inhibiting the activity of hyaluronidase, an enzyme that degrades hyaluronic acid present in extracellular matrix responsible for tumor growth and metastasis. Neo‐menthol reveals better antiproliferative activity by down‐regulating the expression of hyaluronidase in A431 cell lines and it could be taken up for further optimization to yield better efficacy.

## Oxidative stress induced by cadmium sulphide nanoparticles in kidney of rat

**K. Rana, Y. Verma & S. V. S Rana**

*Toxicology Laboratory, Department of Zoology, Chowdhary Charan Singh University, Meerut, Uttar Prades, India*

*Email:* kavitarana.rana5@gmail.com

In recent years cadmium containing nanoparticles have been developed for their application in biology/medical/engineering. Although number of studies have demonstrated that kidney is critical organ in cadmium poisoning but potential health risk associated with use of cadmium sulphide nanoparticles (CdSNPs) has been poorly understood. This encourage us to conduct a experiment comparing nephrotoxicity induced by CdSNPs and bulk cadmium sulphide (CdS). The aim of the present investigation was to determine and compare the nephrotoxicity of CdSNPs and CdS bulk particles in rat employing suitable parameters. Characterization of CdSNPs was done by TEM/SEM/XRD. Renal function tests were determined by commercial kits. Estimation of cadmium concentration in kidney was made by atomic absorption spectrophotometry. Metallothionein in renal tissue of rats was determined by cadmium saturation method. Presence of H2O2 free radicals and lipid peroxidation assay were carried out. Histopathological observation was done by following standard method. Our result indicate that Bioaccumulation of CdSNPs in kidney is lower than CdS bulk particles, however, Metallothionein induction is greater than bulk CdS. Level of creatinine is higher in CdSNPs treated kidney whereas uric acid is elevated in kidney of CdS bulk particles. Induction of H2O2 free radicals are much higher in CdSNPs than bulk CdS. CdSNPs induce more LPO than bulk CdS. CdSNPs cause greater inhibition of SH group than bulk CdS. Extensive proximal tubular necrosis and ultrastructural changes in kidney of CdSNPs were observed. CdSNPs possess specific physicochemical properties and has high potential to cause renal damage through the induction of ROS, oxidative stress, and severe histopathological alteration than CdS bulk particles. These observations conclude that CdSNPs manifest greater toxicity in kidney than CdS bulk particles. Present study suggests that commercial/industrial uses of CdSNPs may pose serious health problem in man.

## Bacterial arginine deaminase: a potent anti‐cancer agent

**K. Bala & A. Sharma**

*Bacteriology Laboratory, Department of P. G. Studies and Research in Biological Science, Rani Durgavati University, Jabalpur, Madhya Pradesh, India*

*Email:* kpradhan456@gmail.com

Cancer is one of the most detrimental disease affects significant segment of human population. Arginine deaminase (ADI) is an anticancer enzyme used in chemotherapy of certain kinds of arginine auxotrophic cancers. However, high antigenicity, shorter serum half‐life and low proteolytic tolerance are the major therapeutic limitations which dramatically hampers clinical efficacy of ADI. The aim of present study was to evaluate anti‐cancer potential of arginine deaminase isolated and screened by using various acid base indicators from various ecological habitats for upgradation of therapeutic index of arginine deaminase therapy. ADI producing indigenous bacterial strains isolated from various ecological habitats by using various acid base indicators (phenol red, bromo cresol purple, bromothymol blue) and investigated *in vitro* serum half‐life, proteolytic tolerance and antitumor activity of crude ADI. Furthermore, potent ADI producing bacterial strains were identified on the basis of morphological, cultural, biochemical characteristics and 16 S rRNA gene sequencing. In the present study, 151 bacterial strains were screened and among them 40 bacterial strains were recorded as ADI positive strains by screening on M‐9 medium containing phenol red. To confirm the ADI activity, positive strains were further screened on M‐9 medium incorporated with bromocresol purple and bromothymol blue as pH indicators. By quantification of ADI activity, among 40 ADI positive strains, eight strains were considered as potential ADI producers. They exhibited ADI activity in the range of 2.91 to 5.56 IU/mL. The crude ADI obtained from these strains were evaluated for in vitro serum half‐life, proteolytic tolerance and anticancer activity. Among 8 strains, crude ADI prepared from PS2 and FB1 showed significant in vitro serum half‐life, strong proteolytic tolerance and potent anticancer activity. On the basis of morphological, cultural, biochemical characteristics and 16 S rRNA gene sequencing these bacterial strains were identified as *Pseudomonas aeruginosa* PS2 and *Sphingobacterium* sp. FB1. From this study, we conclude that ADI from identified bacterial strains could serve as potent anticancer agent. However, more in depth studies are required for strengthening the current findings, which are underway.

## Mutation of Ras gene family in human urothelial carcinoma of bladder

**K. Tripathi^1^, A. Goel^2^, A. Singhai^3^ & M. Garg^1^**

*^1^Department of Biochemistry, Lucknow University, Lucknow, Uttar Pradesh, India; ^2^Department of Urology, King George's Medical University, Lucknow, Uttar Pradesh, India; ^3^Department of Pathology, King George's Medical University, Lucknow, Uttar Pradesh, India*

*Email:* tripathi.krn@googlemail.com

Aberrant RAS/MAPK signaling plays critical role in numerous cellular processes, including proliferation, differentiation, survival and motility. Mutations are identified in various Ras isoforms that belong to Ras gene family. These are mainly point mutations in the hot spot regions of H‐Ras (codon 12; with frequency distribution of mutations: 33%); K‐Ras (codon 12 and 13; with the frequency of 83% of mutation occurrence at Glycine 12 and 14% at Glycine 13); and N‐Ras (codon 61; with the mutation occurrence frequency of 63%). These mutations confer Ras with oncogenic features in a wide variety of human cancers. Mutant RAS oncoproteins are locked in the active GTP‐bound form, initiate kinase cascades to the downstream effectors and confer proliferative advantage to cancer cells. The present study aims to assess the frequency distribution of point mutations in the hot spot regions of Ras gene family in patients diagnosed with urothelial carcinoma of bladder. DNA was extracted from 74 patients enrolled in Department of Urology at KGMU, Lucknow and diagnosed with non‐muscle invasive (40%) and muscle invasive bladder cancer (32%). Point mutations in the hot spot regions of H‐Ras, N‐Ras and K‐Ras were examined by Polymerase chain reaction‐restriction fragment length polymorphism (PCR‐RFLP). None of the patients were identified to harbor point mutations in codons examined in H‐Ras, K‐Ras and N‐Ras. Investigations into the oncogenic activation of Ras likely provide significant insight into bladder cancer pathogenesis. The current observations rule out the possible role of mutations examined in the above mentioned hot spot regions in Ras isoforms. Results of the present study may reflect either the absence of oncogenic functions of Ras or an alternative mechanism for oncogenic transformation in a given cohort of bladder cancer patients.

## miRNA‐128 regulates mitochondrial biogenesis and function in muscle cell physiology

**K. Sharma & N. Saini**

*Functional Genomics Unit, CSIR‐Institute of Genomics and Integrative Biology, New Delhi, India*

*Email:* sharma.kritika130788@gmail.com

Mitochondrial turnover is essential for homeostasis during skeletal muscle adaptations to the environment with respect to its mass and functional capacity, mediated by efficient remodeling mechanism. Impairment of mitochondrial biogenesis and dynamics in is reported to be secondarily correlated with diseases such as type‐ll diabetes, cardiovascular diseases and respiratory ailments leading to loss of muscular mass and increased morbidity and mortality. MicroRNAs are a class of small non‐coding RNAs involved in post‐transcriptional regulation of gene expression. The functionality of miRNA in controlling diverse gene sets in mitochondrial dysfunction makes miRNA an ideal candidate for therapeutic intervention. Recent data demonstrate that the selective modulation of miRNA expression through antisense inhibition or replacement could significantly affect the prognosis and diagnosis of muscle dysfunction in such diseases. The present study aims to study the molecular regulation of mitochondrial biogenesis and function by miRNA‐128. *In silico* analysis using various target prediction tools combined with our previous Microarray data, led us to identify various target genes of miRNA‐128 involved in mitochondrial function and biogenesis. Expressions of genes were validated using TaqMAN PCR, qRT‐PCR and western blotting and the mitochondrial content was assessed using confocal microscopy in C2C12 cells. Level of miRNA‐128 was found to be up‐regulated in myotubes as compared to myoblast. Differential expression of targeted gene such as PGC1 alpha was validated by western blotting along with other downstream genes involved in mitochondrial biogenesis such TFAM, NRF1/2. Gene products involved in mitochondrial function such as DRP1 and MFN1/2 were differentially regulated with over‐expression or silencing of cellular miRNA‐128. PGC‐1 alpha regulates oxidative phosphorylation, mitochondrial biogenesis and oxygen consumption rate. PGC1 alpha is a direct target of microRNA‐128 as predicted by several miRNA target prediction databases. It is reported that microRNA‐128 inhibits muscle cell proliferation and promotes differentiation. Our finding provides us with the clues of miRNA‐128 mediated regulation of mitochondrial biogenesis and function involving crucial mitochondrial fission and fusion proteins connecting with the homeostasis of skeletal muscle physiology.

## Image analysis of lesions for skin cancer

**K. Tiwari^1^, S. Kumar^1^ & R. K Tiwari^2^**

*^1^Amity School of Engineering and Technology, Amity University, Lucknow, Uttar Pradesh, India; ^2^Department of Physics and Electronics, Dr. RML Avadh University, Faizabad, Uttar Pradesh, India*

*Email:* kumud.1992@gmail.com

In the world today there are number of skin diseases which are found in humans. The illness caused due to bacteria or infections is known as skin disease like yeast infection, allergy, eczema, brown spot. These skin diseases have dangerous effect on skin and they keep on spreading over time. To control them from spreading it is necessary to identify these diseases at nascent stages. In human, skin cancer is the most prevalent and lead to serious consequences. Melanoma is the most fatal type of skin cancer, physicians face various difficulties in accurate diagnose of lesion through naked eye. Over the two decades, computerized analysis of PSLs that is pigmented skin lesions has been an active area of research. Monitoring every patient costs high, so an automated system is a needed to determine a patient's risk of skin cancer, through images analysis of lesions which are captured by dermatoscope. For that it is necessary to develop reliable automatic methods for recognizing skin cancer from images acquired *in vivo* and to increase the accuracy of the diagnostic. The technologies to identify these disease generally involve image processing, artificial neural network, data mining etc. The paper presents a review of various image processing techniques which are used for diagnosis of skin diseases in recent time and to provide an extensive introduction to and clarify ambiguities in the terminology used in the literature. Analysis of the different methodologies and their performances that are utilized in these techniques of skin diseases diagnosis has also be done.

## 3‐Hydroxy‐11‐keto‐*β*‐boswellic acid: an antiproliferative compound isolated from *Boswellia serrata*

**M. Gupta, S. Singh, S. Luqman, R. S. Bhakuni & P. K. Raut**

*Chemical Sciences Division & Molecular Bioprospection Division, CSIR‐Central Institute of Medicinal and Aromatic Plants, Lucknow, Uttar Pradesh, India*

*Email:* 19madhurigupta@gmail.com

Increasing research on traditional herbal medicines and their phyto‐constituents has recognized their use fullness in complementary as adjuvant to chemotherapy in various types of cancers. The oleo‐gum resin of *Boswellia serrata* tree is one such traditional medicine, which has been regularly used for religious, cosmetic as well as medical purpose since ancient. Recently, the potential chemopreventive and therapeutic effects of boswellic acids on cancers have started to draw attention. The acids have been shown to inhibit growth of so many tumours like brain tumours, Breast cancer, induced apoptosis in leukemic cells and hepatoma cell line. Because of the relevance for the clinical application, we tested the methanolic extract of *B. serrata* gum resin containing a defined amount of KBA for its anticancer activity on eight cell lines WRL‐68, A 549, COLO‐205, K562, MDAMB231, HaCaT, A431 and HEK‐293 using MTT assay. This study also revealed the comparisons of their anticancer activity between methanol extracts of oleo‐gum‐resin obtained from natural and commercial habitat and the yield of KBA by HPLC profiling. HPLC analysis was performed on a WATERS (Miford, MA, USA) PDA (model 1996) and separations were achieved using a Waters reverse phase C18 column (250 × 4.6 mm i.d.; 5 *μ*m) subjected to isocratic elution using ACN (solvent A) and water with 0.1% acetic acid (solvent B). Antiproliferative potential of extracts and isolated compounds was evaluated using MTT assay. All the extract, fraction and isolated compounds inhibit the proliferation of cancer cell lines with the percent inhibition of 13.07 to 93.05%. The KBA induced dose‐dependent antiproliferative effects on all human malignant cells (IC50 values 4.10–37.94 *μ*g/mL). Among the screened compounds, 3‐hydroxy‐11‐keto‐*β*‐boswellic acid has been found to be most promising antiproliferative compound. The triterpenoidal fraction and extracts of *B. serrata* (containing boswellic acids) is responsible for the antiproliferative properties. KBA showed good antiproliferative activity and oleo‐gum‐resin had good content of it. So the molecule can be used for further biological evaluations.

## Doxorubicin loaded Selenopolymeric nanocomposites: a smart tool to achieve synergistic cancer cell death

**M. P. Purohit^1,2^, N. K. Verma^2^, A. K. Kar^1,2^, A. Singh^2^, D. Ghosh^1,3^ & S. Patnaik^1,2^**

*^1^Academy of Scientific and Innovative Research (AcSIR), CSIR‐IITR Campus; ^2^Water Analysis Laboratory, Nanotherapeutics & Nanomaterial Toxicology Group, CSIR‐Indian Institute of Toxicology Research (CSIR‐IITR), Lucknow, India; ^3^Immunotoxicology lab, Food Drug and Chemical Toxicology group, CSIR‐Indian Institute of Toxicology Research (CSIR‐IITR), Lucknow, India*

*Email:* mahaveer23.purohit@gmail.com

Cancer mono‐chemotherapy is often associated with poor pharmacokinetics and systemic toxicity which leads to dose limiting inadequacy of efficacy. Nanomedicine productively overcomes the shortcomings of conventional chemotherapy. Recently, Selenium Nanoparticles (Se NPs) emerged as promising drug delivery carriers as well as cancer therapeutic agents. So, implication of selenium nanocarriers to deliver chemotherapeutic drug Doxorubicin (DOX) could be a great way to achieve anticancer synergism. To enhance the therapeutic performance of DOX by using selenium based nanocarrier. Targeting ligand hyaluronic acid (HA) functionalized selenopolymeric nanocarriers (Se@CMHA NCs) was synthesized and characterized using different analytical techniques. The activity of designed nanosystems (Se@CMHA‐DOX NCs) was evaluated through various *in vitro* assays such as cell viability assay, cellular internalized study, thioredoxin reductase activity assay and detection of apoptosis on human breast cancer cell line, MCF7. The therapeutic potency wielded by the developed nanosystem was also adjudged in actual *in vivo* settings using 3D tumor sphere model. The size of the Se@CMHA‐DOX NCs was recorded to be 244 ± 6.8 nm. Se@CMHA‐DOX NCs exhibited enhanced cytotoxic potential towards human cancer cells compared to free DOX in an equivalent concentration. The various qualitative and quantitative data clearly indicated the successful cellular internalization of nanosystem. Thioredoxin reductase (TrxR) activity was inhibited following Se@CMHA‐DOX NCs exposure. Detailed molecular studies on MCF7 cells also established that upon exposure to Se@CMHA‐DOX NCs, MCF7 cells endure G2/M cell cycle arrest and p53 mediated caspase independent apoptosis. The final nanoformulation has selectivity between malignant and normal cells and higher therapeutic potential compare to the bulk DOX owing to the synergistic interplay between DOX and Se NPs. Mechanistic studies revealed that, Se NPs of nanocarriers progressively inhibit TrxR activity, which further augments the chemotherapeutic action of DOX. To the best of our knowledge, our study is first to report about the TrxR inhibitory effect of Se NPs. Overall Se@CMHA‐DOX NCs modulates the intracellular redox balance, which leads to oxidative stress mediated apoptotic cell death. The aforementioned observations clearly delineate Se@CMHA NCs as a promising nanocarrier for DOX, additionally synergizing its chemotherapeutic potential and offer a viable modality for effective cancer chemotherapy.

## Chronic exposure of low dose Bisphenol A enhances proliferating potential of breast cancer cells via genomic instability

**M. I. Ansari, V. K. Singh & P. K. Sharma**

*^1^Environmental Carcinogenesis Laboratory, Food Drug and Chemical Toxicology Group, CSIR‐Indian Institute of Toxicology Research, Lucknow, India; ^2^Academy of Scientific and Innovative Research, New Delhi, India*

*Email:* imranansarilko@gmail.com

Humans are routinely exposed to environmental relevant dose of Bisphenol A (BPA). BPA is a high volume produced plasticizer used in the production of food and beverage container worldwide. Although a number of studies have investigated the adverse effects of high dose as well as acute exposure of BPA. However, there has been a lack of mechanistic study of chronic effect of low dose BPA. In the present study we have investigated that chronic exposure (200 days) of BPA enhanced cellular proliferation and migration potential in human breast cancer cells. Various parameters such as MTT assay, growth kinetics for proliferation and soft agar assay for anchorage independent growth etc were performed. As compared to acute exposure, the chronic exposure of BPA significantly enhanced the proliferation as well as mitochondrial biogenesis in ER‐dependent MCF‐7 breast cancer cells. Moreover, the chronic exposure of BPA enhanced micronuclei formation (MN) and DNA double stranded breaks (DSBs) in hormone dependent breast cancer cells. Furthermore, these genomic alterations enhanced proliferation rate and anchorage independent growth in MCF‐7 cells. Here, we concluded that chronic exposure of low dose BPA significantly promote cancer proliferation and progression via genomic alterations. However, the mechanism underlying such BPA‐mediated altered growth and proliferation in ER‐dependent breast cancer cells needs more critical evaluation.

## Lead nitrate‐induced ROS mediated DNA damage and cell apoptosis in human renal proximal tubular epithelial cell: amelioration via N‐acetyl cysteine, Curcumin and Tannic acid

**M. Siddarth^1^, D. Chawla^1^, A. Raizada^2^, B. D. Banerjee^3^ & M. Sikka^4^**

*^1^Multidisciplinary Research Unit, University College of Medical Sciences (University Of Delhi) and G.T.B. Hospital, Delhi, India; ^2^Department of Medicine, University College of Medical Sciences (University Of Delhi) and G.T.B. Hospital, Delhi, India; ^3^Biochemistry, University College of Medical Sciences (University Of Delhi) and G.T.B. Hospital, Delhi, India; ^4^Pathology University College of Medical Sciences (University Of Delhi) and G.T.B. Hospital, Delhi, India*

*Email:* manushisiddarth@gmail.com

Lead is ubiquitous, widely distributed industrial metal and is one of the most important current global environmental toxicant. Lead affects numerous organ systems including kidney, but the specific mechanisms of damage are not well known. Oxidative stress is a key factor in lead‐associated kidney damage, but it has been unclear how the stress is generated. Thus far, many scientific reports on lead have been focused on its immunotoxic effect on experimental animal and people with occupational exposure to lead. Presently, there is no study on the influence of lead exposure on renal cell line. This study investigates the exposure of lead‐induced reactive oxygen species (ROS) generation, DNA damage and apoptosis and also evaluates the therapeutic intervention using antioxidants in human renal proximal tubular cells (HK‐2 cells). HK‐2 cells (CRL‐2190, ATCC, USA), were treated with increasing concentration of lead nitrate (0–50 *μ*mol/L) (Sigma, USA). Cell cytotoxicity was measured by MTT assay. ROS level was determined by measuring fluorescence of 2,7‐dichlorofluorescein (DCF) which is a hydrolyzed product of H2DCF‐DA. GSH level and Caspase‐3 activity were evaluated by spectrofluorometric method using standard protocol. DNA damage was measured by comet assay. Following treatment of HK‐2 cells with an increasing concentration of lead nitrate (0–50 *μ*mol/L) for 24 h, intracellular ROS level increased whereas GSH level decreased significantly in a dose‐dependent pattern. Comet assay results revealed that lead nitrate showed the ability to increase the levels of DNA strand breaks in HK‐2 cells. Lead exposure also induced apoptosis through caspase‐3 activation at 30 *μ*g/mL. Pretreatment with N‐acetylcysteine (NAC), Curcumin and Tannic acid showed significant ameliorating effect on lead‐induced ROS, DNA damage and apoptosis. Lead induces ROS, which may exacerbate the DNA damage and apoptosis via caspase‐3 activation. Additionally, supplementation of antioxidants like NAC, Curcumin and Tannic acid may be used as salvage therapy for lead‐induced DNA damage and apoptosis in exposed person.

## Genotoxic effects induced by arsenic, cadmium, chromium and nickel in peripheral erythrocytes of a fish, *Channa punctatus*

**M. Singh, Y. Verma & S. V. S. Rana**

*Toxicology laboratory Zoology Department, Chowdhary Charan Singh University, Meerut, Uttar Pradesh, India*

*Email:* smeenu563@gmail.com

Heavy metal contamination in aquatic environment is a worldwide problem. The important problems are those that they tend to accumulate in aquatic organisms and attain persistence due to their stability or slow biodegradability. Nevertheless, aquatic environment is the ultimate recipient of an increasing amount of contaminants as a result of the discharge of industrial, agricultural and urban wastes. Carcinogenic and mutagenic compounds especially arsenic, cadmium, chromium and nickel are most problematic as their effects may exert damage beyond that of individual effects and may remain active through following generation. Genotoxic effects as main biomarkers in assessment of the pollution related toxicity should be member of the test battery. It has been suggested that a variety of biomarkers and bioassay in the laboratory and field studies be used in determining their genotoxicity. Present study was performed in a native fresh water fish *Channa punctatus* to record the genotoxic effect of arsenic, cadmium, chromium and nickel by estimating the DNA damage, micronuclei frequency, nuclear anomalies and oxidative stress in peripheral erythrocytes. The main objective of the study has been to correlate DNA damage by comet assay, Micronuclei (MN) assay with fresh water pollution caused by these carcinogenic metals. For the present study the healthy and disease free fishes, *Channa punctatus* were collected from local market. Fish after acclimatization to laboratory condition were divided into five groups. Minimum of 10 fish per groups were exposed to respective metal (0.5 ppm/each metal) for 30 days and one group served as control. Peripheral blood samples were obtained from caudal vein of experimental fishes after 30 days of exposure to respective metal. Blood samples were processed for DNA damage by comet assay, MN frequency and nuclear anomalies and oxidative stress. Present result shows that DNA damage and MN frequency varies from metal to metal. However maximum effect was recorded in arsenic and nickel exposed fishes followed by chromium and cadmium. In addition to the formation of micronuclei and DNA damage, changes in nuclear morphology were also recorded. The selected metals viz. arsenic, nickel, chromium and cadmium are potent induces of oxidative stress in the fish. The oxidative stress leads to the formation of MN, DNA damage, nuclear anomalies resulting genotoxicity. Present study reveals that arsenic, nickel, cadmium and chromium are genotoxic/clastogenic chemicals inducing MN, DNA damage and nuclear anomalies in erythrocytes of fish. Thus it can be used as a biomarker for monitoring pollution in aquatic environment.

## Exposure to Alternariol caused genomic instability and tumor initiation in mouse skin

**M. Bansal^1,2^, N. Singh^1,2^, S. Pal^1,2^, I. Dev^1,2^ & K. M. Ansari^1^**

*^1^Food Toxicology Laboratory, Food, Drug, and Chemical Toxicology Group, CSIR‐Indian Institute of Toxicology Research, Lucknow, Uttar Pradesh, India; ^2^Academy of Scientific and Innovative Research (AcSIR), CSIR‐IITR Campus, Lucknow, Uttar Pradesh, India*

*Email:* kmansari@iitr.res.in

Alternariol (AOH), a natural contaminant of food crops pose health risk to manual labour employed in agricultural practices via dermal exposure. This study aimed to assess damage caused by AOH at molecular level in intact mouse skin on acute and chronic exposure. Recently several *in‐vitro* studies have reported AOH metabolism and its role in causing oxidative stress, mitochondrial damage, abnormal nuclear morphology, cell cycle arrest and senescence in different cell lines. Based on these studies, it was hypothesized that AOH can cause damage to DNA integrity which, if not repaired precisely, can lead to tumor development in mouse skin. In this study, we treated *Swiss* mice(n = 5/group)with AOH 100 *μ*g/animal for time dependent assessment (1 h, 3 h, 6 h). In addition, to evaluate its carcinogenic potential, mice were divided into five groups (n = 15/group) consisting of (i) control (Acetone) (ii) positive control (DMBA + TPA twice weekly) (iii) tumor initiator (100 *μ*g/animal AOH followed by TPA) (iv) tumor promotor (DMBA + 10 *μ*g/animal AOH twice weekly) and (v) complete carcinogen testing group (100 *μ*g/animal AOH followed by 10 *μ*g/animal AOH) for 24 weeks. We found elevated ROS production, reduced total antioxidant potential and GSH depletion in skin samples. Moreover DNA damage markers, 8‐hydroxyguanosine and gamma H2AX levels were evaluated in skin sections. The results indicated that acute AOH exposure lead to double‐strand breaks and oxidative DNA damage in mouse skin. In chronic study. we found 60% tumor incidence in tumor initiator group, 13.3% tumor incidence in tumor promoting group while AOH does not caused any tumor incidence on tested dose when it is used as a complete carcinogen. These findings suggest that AOH can act as skin tumor initiator lacking tumor promoting properties in mouse model and hence can be regarded as incomplete carcinogen.

## Upstream regulation of mTORC2: a study to unravel the role of superoxide anion O2^−^

**M.‐U. Lone^1^, M. Asif^1^, S. A. Malik^1^, R. Shrivastava^1,2^, P. Dubey^3^, V. Singh^1^, J. Miyan^1^ & S. Bhadauria^1,2^**

*^1^Division of Toxicology and Experimental Medicine, Central Drug Research Institute (CSIR), Lucknow, Uttar Pradesh, India; ^2^Academy of Scientific and Innovative Research (AcSIR), New Delhi, India; ^3^Department of Surgical Oncology, KGMU, Lucknow, Uttar Pradesh, India*

*Email:* smraticdri@gmail.com

The mammalian target of rapamycin (mTOR) associates with different subunits forming functionally distinct complexes, mTORC1 and mTORC2.Rheband Rag, small GTP‐binding proteins regulate mTORC1, however regulation of mTORC2 remains obscure. Studies conducted in *Dictyostelium* suggest for possible role of Ras, as a potential upstream regulator of mTORC2, definitive studies delineating the molecular mechanisms in mammalian cancer cells are still lacking. To evaluate superoxide anion mediated activation of mTORC2 signaling in mammalian cancer cells. Expression of mTORC2 specific markers was analyzed by western blotting. Intracellular superoxide anion level was measured using dihydroethidium probe. Stress fibre formation and Ras localization was assessed by immunofluorescence analysis. Pyrogallol, a potent superoxide anion generator, was utilized for investigating the effect of superoxide anions with respect to mTORC2 signaling. The optimum concentration of pyrogallol at which cells exhibited elevated O2^−^ levels without any loss of vitality was deduced to be 20 *μ*mol/L. mTORC2 specific markers revealed maximal activation of mTORC2 at 20 *μ*mol/L, with a plateau at higher concentrations. Pyrogallol induced mTORC2 activation was markedly abrogated in MDA‐MB‐231 cells that were pre‐treated with MnTBAP, a superoxide dismutase 2 (SOD2) mimetic and a superoxide scavenger, thereby establishing the role of O2^−^ as an activator of mTORC2. Confocal microscopy revealed greater abundance of stress fibres in pyrogallol treated cells as compared to that in control and cells pre‐treated with MnTBAP. Cells stimulated with pyrogallol exhibited increased redistribution of Ras to plasma membrane which in turn was an indicator of heightened Ras activation. Cells treated with farnesyl transferase/geranylgeranyl transferase inhibitors (FTI/GGTI) prior to pyrogallol stimulation, exhibited attenuated mTORC2 signaling. Based on previous finding that 17‐*β*‐estradiol causes mTORC2 activation in ER^+^ breast cancer cells in a superoxide dependent manner, we set out to decipher as to how might superoxide anion engage mTORC2. We demonstrated that stimulus accounting for elevated superoxide anion eventually culminate into potentiated mTORC2 cascade. This superoxide anion dependent mTORC2 potentiation correlated with activation of Ras. We conclude that superoxide anion stimulated activation of mTORC2 signaling in mammalian cancer cells is a Ras dependent phenomenon.

## Evaluation of antiproliferative activity and metabolic profiling of *tridex procumbens*

**M. Shukla^1,2^, A. A. Mahdi^1^, R. Singh^1^ & M. K. Ahmad^1^**

*^1^Department of Biochemistry, King George's Medical University, Lucknow, Uttar Pradesh, India; ^2^Department of Biotechnology, Dr. A.P.J Abdul Kalam Technical University, Lucknow, Uttar Pradesh, India*

*Email:* msshukla2012@gmail.com

Breast cancer is the most common malignant tumor and second leading cause of death in women worldwide. It is a heterogeneous multifactorial disease caused due to genetic, reproductive, environmental, and dietary and lifestyle related risk factors. Varieties of phytochemicals are found in medicinal plants which are frequently used in healing of various diseases including cancer. Selecting new anticancer drug from herbal world is important in both biological and pharmacological activities. The medicinal values of many plants have been explored but a large number of them are unexplored, *tridax procumbens* is one of them. The aim of this study is to explore the anti‐proliferative activity of aerial parts of *t. procumbens* against human breast cancer (mda‐mb‐231 & mcf‐7) cell lines. Our in vitro results indicated that hexane extract of *t. procumbens* flower part (htpf) exhibited significant antiproliferative activity against both these cell lines due to induction of cancer cell death through apoptosis. It also exhibited increased anti‐metastatic effect against the cancer cells which was calculated by wound healing assay. htpf induced cell death is also detected by colony formation assay. GC‐MS analysis exposes the presence of several metabolites in *t. procumbens*. Henceforth, this would be called htpf could be used for further analyses to understand its mode of action on induced apoptosis. The overall results propose that the selective extract htpf has shown potent anti‐cancer activity in a dose‐dependent manner. Our findings provide a vision into the potential of *t. Procumbens* as a novel drug for killing cancer cells. Breast cell lines (mda‐mb‐231 &mcf‐7) along with the human normal transformed (hacat) cells were selected for mtt analysis. Flower part of t. procumbens dissolve in hexane solvent (i.e. Hexane extract) showing most significant ic50 value (p‐value ≤ 0.05) could be considered as a potent anti‐proliferative agent. We then further evaluated the anti‐ metastatic property using wound healing assay. Our results revealed that hexane extract of *t. procumbens* flower exhibit highly significant antiproliferative and anti‐metastatic activity against both the cell lines (mda‐mb‐231, mcf‐7). Based on our finding and available literature there is need for isolation and characterization of specific bioactive compounds of *t. Procumbens* and determination of their mechanism of action responsible for its anticancer activity in breast cancer cells.

## Nimbolide ameliorates anti‐cancer drug Hydroxyurea induced clastogenicity and oxidative damage

**M. O. Ansari^1^, M. F. Ahmad^1^, H. R. Siddique^2^ & G. G. H. A. Shadab^1^**

*^1^Cytogenetics and Molecular Toxicological Laboratory, Section of Genetics, Department of Zoology, Aligarh Muslim University, Aligarh, Uttar Pradesh, India; ^2^Molecular Cancer Genetics & Translational Research Lab, Section of Genetics, Department of Zoology, Aligarh Muslim University, Aligarh, Uttar Pradesh, India*

*Email:* gghas.amu@gmail.com, hrsiddique@gmail.com

Nimbolide is known to be an anti‐inflammatory and anti‐mutagenic component found in *Azadiracta indica* L. (neem plant). Hydroxyurea (HU) is frequently used in the treatment of several types of cancers, sickle‐cell anemia and HIV infection.HU is considered to be a genotoxic agent and a presumable trans‐species carcinogen. Therefore, the aim of the present study was to evaluate the genotoxic effect of HU and possible amelioration of nimbolide in HU induced clastogenicity and oxidative stress in Wistar rats. To test our hypothesis, we performed chromosomal aberrations assay, micronucleus test, histopathological studies and lipid peroxidation assay. Our results showed that HU is a clastogenic chemical caused various chromosomal structural abnormalities and micronuclei formation. The observed genotoxic effect might be due to the high level of reactive oxygen species (ROS) as HU increased significant high level of malondialdehyde (MDA), a biomarker of oxidative damage, in a dose dependent manner. Treatment of animals with nimbolide showed the significant lower level of HU induced clastogenicity. Based on our study, we conclude that HU caused genetic damage possibly through ROS. Further, nimbolide ameliorate HU induced clastogenic and oxidative damage.

## Remediation of hormone refractory breast cancer via co‐loaded phytoliposomes

**M. Dwivedi, S. Agrawal, V. Teja, R. Shukla, S. Urandur & P. R. Mishra**

*Pharmaceutics and Pharmacokinetics Division, CSIR‐Central Drug Research Institute, Lucknow, Uttar Pradesh, India*

*Email:* monika.nbri@gmail.com

The perseverance to develop effective alternative approaches for management of refractory hormone cancers pave the way for phytocombination therapy for cancer prevention and treatment. Curcumin (CUR) is a major dietary food component of the flavoring agent turmeric (*Curcuma longa L*.) in Asian diets with broad biological outcomes exploited against hormone independent cancers. Ursolic acid (UA) is a pentacyclic triterpene, abundantly distributed in fruits and vegetables. However, various molecular inheritances have shown to control the oral delivery of such phytochemicals due to their hydrophobicity that accounts for its low therapeutic index. The main objective assessed with the present study was development of a co‐loaded curcumin and ursolic acid liposomes (UA‐CUR‐LiP) to synergies the anticancer therapeutic effect on hormone refractory cancers. The liposomes can evade the obstacles of extremely poor solubility and permeability of CUR and can help in achieving ultimate intention of designing a combination therapy with increased therapeutic potential. The co‐encapsulated nanoliposomes were formulated by thin‐film hydration method. The formulation design was optimized for lipid compositions and molar ratio of HSPC/ PC/Chol. The nanosized UA‐CUR‐LiP has been achieved which have consistent spherical morphology with particle size 126.5 ± 3.22 nm and zeta potential of −26.4 ± 2.11 mV. The ratio metric combination for CUR and UA as liposomes has shown significantly enhanced cytotoxicity than the individual CUR and UA in MDAMB 231 cells. Fluorescent microscopy confirms the internalization ability by targeted delivery of the CUR loaded UA‐CUR‐LiP evaluated in MDAMB 231 breast cancer cells. Mechanistic studies in MDAMB 231 cells indicated that the apoptosis was mediated through mitochondrial membrane potential disruption assisted with elevated levels of free radicals in cancer cells. The *in vivo* antitumor effect was studied in 4‐T1 induced Balb/c mice mammary tumor model vindicated the elevated anticancer effect by UA‐CUR‐LiP in comparison to CUR‐LiP. Emphasized by our results, the co‐loaded liposome of CUR and UA could be a hallmark for innovative combination chemotherapy with improved clinical outcomes for hormone refractory breast cancer.

## Gfi‐1 Levels regulate hematopoietic stem cell reconstitution and leukemic cell growth by inducing cell cycle arrest and differentiation

**M. Ali^1^, H. Li^2^, K. D. Klarmann^2^, J. R. Keller^2^ & S. K. Singh^1^**

^1^Stem Cell/Cell Culture lab, Center for Advance Research, King George's Medical University, Lucknow, Uttar Pradesh, India;

*^2^Mouse Cancer Genetic Program, BRP, Leidos Biomedical research, Inc., Frederick National Lab, NCI‐NIH, Frederick, MD, USA*

*Email:* murtazaali90@gmail.com

Hematopoietic Stem and Progenitor Cells (HSPC) are responsible for maintaining the production of all blood cells over the life of the animal through an ordered process of proliferation and differentiation. Transcription factors and transcription factor networks are critical for HSC maintenance and function. Gfi‐1 is a zinc finger transcription factor that is required to maintain normal HSC numbers, and for proper development of B, T and myeloid cells *in vivo*. In humans, hypomorphic mutations of Gfi‐1 cause severe neutropenia and lymphocytopenia. HSC and committed myeloid progenitors from Gfi‐1^−/−^ mice show increased proliferation and are blocked myeloid differentiation, suggesting that Gfi‐1 acts as a tumor suppressor by regulating HSPC proliferation and differentiation. We asked if Gfi‐1 overexpression (OE) inhibit the growth of HSPC, and found that Gfi‐1OE inhibited human and mouse leukemic progenitor cell proliferation in a dose dependent manner *in vitro*. Furthermore, we found that the engraftment of B, T and myeloid cells were reduced by 95% in mice transplanted with Gfi‐1 OE bone marrow cells in dose dependent manner, indicating that Gfi‐1 inhibits HSC function *in vivo*. We confirmed that levels of Gfi‐1 were critical to maintain HSC quiescence since Gfi‐1^+/−^ HSC are hyper proliferating *in vivo* compared Gfi‐1^+/+^. As a consequence of hyperproliferation, in BM transplant Gfi‐1^+/−^ HSC exhaust and showed lower engraftment compare to Gfi‐1^+/+^.To identify novel Gfi‐1 targets that restrict HSC proliferation, we examined gene expression in Gfi‐1 OEHSC and found roughly 1500 genes were decreased more than 1.5‐fold. Gfi‐1 and Hoxa9 can coordinately function as a molecular switch to regulate hematopoietic cell fate. Therefore, we compared our Gfi‐1 OE array data with Hoxa9 OE array and Chip on Chip data, and obtained a set of overlapping genes like Erg, Sox4, CD34. Over expression of these target genes are associated with hyperproliferation of HSC. These results suggest that Gfi‐1 is required to restrict HSC proliferation and levels of Gfi‐1 critically regulate the balance between its exhaustion and differentiation.

## Chemokine receptor CXCR4 but not CXCR7 promotes drug resistance in cancer via death receptor downregulation

**S. Maheshwari, M. A. Nengroo, A. singh, P. Chaturvedi, A. Verma & D. Datta**

*Biochemistry Division, CSIR‐Central Drug Research Institute, Lucknow, Uttar Pradesh, India*

*Email:* mushtaqcdri@gmail.com, mushtaqan.20@gmail.com

Chemokine receptor CXCR4 and its ligand CXCL12 (SDF‐1) have shown to be overexpressed in more than 20 solid tumors and CXCR4 serves as a Cancer Stem Cell (CSC) marker in multiple cancers. However, the novel involvement of CXCR7 as a high affinity‐binding partner of CXCL12 begs for reconsideration of the whole CXCR4 paradigm in the context of cancer biology. Here, we aim to study the contribution of CXCR4 and CXCR7 in modulating CSC properties in cancer. MCF‐7 (null for CXCR4/CXCR7 surface expression) and HT29 (both positive) were selected for overexpression and knockdown studies respectively. Stable overexpression and knockdown cells for CXCR4 and CXCR7 were generated by transfection of respective plasmids with antibiotic selection. Spheroid formation, aldefluor, colony formation, and SRB assays were used to determine CSC properties. Proteome Profiler Array was used to determine the expression of 35 apoptosis related genes. Expression analyses were done by performing Real‐Time PCR, Western Blot, Flow cytometry. ChIP assay was used for the analysis of YY1 and p53 recruitment on *DR5* gene promoter. FDA approved drugs Doxorubicin, Paclitaxel, Cisplatin, and 5‐Fluorouracil were used to assess drug resistance in cancer cells. CXCR4 but not CXCR7 overexpression promoted marked increase in CSC properties as evidenced in generation of higher number of CD44^+^ CD24^−^ breast CSCs, robust spheroid formation, and significant Paclitaxel resistance as compared control parental cells. To find out underlying mechanisms of CXCR4 mediated drug resistance, we discovered that CXCR4 posed very selective downregulatory effect on pro‐apoptotic Death Receptor (DR) 5 expression at both mRNA and protein levels via modulating transcription factors p53, YY1, and Sp1. In depth promoter analysis of DR5 and ChIP assay further indicate that CXCR4 is actually reversely regulating the recruitment of repressor YY1 and inducer p53 to the promoters of DR5. In contrast, knockdown of either CXCR4 or restoration of DR5 in CXCR4 overexpressed cells sensitizes resistant cells to chemotherapeutics. High expression of CXCR4 but not CXCR7 restricts cellular apoptosis via downregulating DR5 protein through differential expression and recruitment of p53 and YY1 transcription factors and thus renders cancer cells resistant to chemotherapeutic drugs.

## Novel combinatorial *Therapeutics* targeting cancer epigenetics and metabolism in human triple‐negative breast cancer

**J. Natesh, D. Penta, P. Mondal, B. S. Somashekar & S. M. Meeran**

*Laboratory of Cancer Epigenetics, Department of Biochemistry, CSIR‐Central Food Technological Research Institute, Mysuru, Karnataka, India*

*Email:* s.musthapa@cftri.res.in

Triple‐negative breast cancer (TNBC) is the most aggressive type of breast cancer which is resistance to available hormonal therapies such as tamoxifen and aromatase inhibitors. Epigenetic modulations like DNA methylation, histone modification and miRNA‐mediated transcriptional regulation play an important role in breast cancer progression. Any aberration in epigenetic regulation will alter the expression profile of tumor suppressor genes and tumor promoter genes. Targeting epigenetics in cancer is gaining importance in determining better therapeutic approach. Here we present a novel approach towards the treatment of TNBC through the combinations of natural dietary component 3,3′‐diindolylmethane (DIM) and an oral contraceptive centchroman (CC).We observed that the combinations of DIM and CC synergistically inhibit the cell proliferation and inducing cellular apoptosis of TNBC cells significantly compared to the respective individual doses of these compounds. Intriguingly, DIM and CC alone as well as in combinations inhibit stemness of the TNBC cells. The expression profile of epigenetic modulatory enzymes such as DNA methyltransferases (DNMTs) and histone deacetylases (HDACs) were found to be significantly altered by the combinations of DIM and CC in TNBC cells. Further, we performed NMR‐based metabolomics study on TNBC MDA‐MB‐231 cells with treatment of DIM and CC individually, and in combinations of these molecules. ^1^H NMR spectra of polar extracts were used to identify a wide range of metabolites involved in glycolysis, TCA‐cycle, glutaminolysis, energy metabolism, hexosamine pathway, membrane choline phospholipid metabolism and osmo‐regulatory mechanisms. The combinations of DIM and CC, synergistically alter these key energy metabolic pathways in human TNBC cells. Therefore, studying the combinatorial effect of these compounds and their epigenetic modulations as well as metabolic changes can enhance the knowledge which further can be developed as an effective treatment regime against triple‐negative breast cancer.

## Phytochemical screening and chondrogenic efficacy of *Solanum xanthocarpum* ethanolic fruit extract on oxidative stressed primary chondrocytes and prevention of cartilage damage in osteoarthritic induced rat

**N. Shivnath, V. Rawat, Sahabjada, A. Jafri, M. Sajid Khan & M. Arshad**

*Molecular Endocrinology lab, Department of Zoology, University of Lucknow, Lucknow, Uttar Pradesh, India*

*Email:* neelamshivnath@yahoo.co.in

The present study aimed to find out the bioactive compounds present in the ethanolic extract of *Solanum xanthocarpum* fruit (SXF) through GCMS and to evaluate the chondrogenic efficacy on primary chondrocytes and prevention of cartilage damage in collagenase type‐II induced osteoarthritis in rat. The chondrogenic efficacy was evaluated by cell viability assay and cell cycle analysis to study the proliferation of chondrocytes, antioxidant and antiapoptotic efficacy was measured by ROS assay, MTT, DAPI and Hoechst‐PI staining. Chondroprotective activity was illustrated *via* changes in weight of rats, ALP activity, histopathological changes in knee joint, proteoglycan, collagen content and by mRNA expression of Col‐2, MMP‐3 and COX‐2 genes. SXF shows the presence of various phytoconstituents as revealed from GCMS. The results also suggest that SXF promoted the viability of chondrocytes, inhibits the oxidative stress and apoptosis in dose dependent fashion. The treatment of diseased rats with SXF for 30 days reduces symptoms of disease with conservation of cartilage structure, retention of proteoglycan and collagen content. The ALP level was significantly (*P* < 0.001) reduced in all treated group. The expression of Col‐2 gene was up‐regulated while the expression of MMP‐3 and COX‐2 gene was down regulated compared to control. Findings suggest that SXF contains various secondary metabolites that act synergistically to protect chondrocytes through inducing cell proliferation and preventing oxidative stress and apoptosis. The study also reflects the chondroprotective tendency of the SXF in an established osteoarthritic rat model.

## IL‐6 protects the cells from radiation induced cell death by switching metabolism and activating STAT‐3 mediated pro‐survival signalling

**N. Kumari^1,2^, Y. Rai^1^, D. K. Sah^1^, A. Das^2^ & A. N. Bhatt^1^**

*^1^Institute of Nuclear Medicine and Allied Sciences, Timarpur, Delhi, India; ^2^Department of Biotechnology, Delhi Technological University, Delhi, India*

*Email:* kmneeraj05@yahoo.com

IL‐6 is one of the known molecules that induce radio‐resistance in cancer cells by regulating multiple signaling pathways including apoptosis, survival, proliferation, angiogenesis, and most importantly, the metabolism. Thereby, acting as a major obstacle in radiotherapy. Apart from this IL‐6 also protects normal cells like cardiomyocytes, hepatocytes, retinal ganglion, spleen and neuronal cells from oxidative stress, ischemia/reperfusion injury and other stress induced cell death. Therefore, we tested the hypothesis, if IL‐6 treatment before irradiation will confer radio‐resistance in normal cells and protect them from radiation induced cell death. We used the pleiotropic IL‐6 as a prophylaxis for radiation exposure in hematopoietic (Raw264.7), fibroblast (NIH3T3) and intestinal epithelial (INT407) cell lines. First we observed the increase in glycolysis by measuring lactate production and glucose uptake. Then we wanted to check the increase in cell survival and we found a significant increase in cell number (1.3 fold in INT407 cells to 1.4 fold in Raw264.7 cells) after IL‐6 treatment prior(2 h) to radiation exposure. Further, we found that IL‐6 reduces radiation induced cell death/apoptosis which is substantiated by the levels of pro and anti apoptotic proteins. It was observed that STAT‐3 was phosphorylated within 15 min after IL‐6 treatment and inhibition of STAT‐3 phosphorylation using JSI‐124 abrogates the IL‐6 mediated protection from radiation induced cell death. Moreover inhibition of glycolysis by using 2DG and 3 bromopyruvate also reverses the IL‐6 mediated radioprotection. IL‐6 also induces the Nrf2 expression and maintains the antioxidant status of the cells. In our study we found increased levels of GSH, SOD, and reduced ROS, lipid peroxidation and protein carbonylation. Therefore, we can conclude that IL‐6 protects cells from radiation induced cell death by switching metabolism through STAT3 activation that further activates multiple downstream pathways resulted in enhanced antioxidant defense and STAT‐3 mediated pro‐survival signaling.

## Neoplastic transformation of intestinal cells by natural food contaminant Patulin

**N. Singh^1,2^, S. Pal^1,2^, M. Bansal^1,2^, I. Dev^1,2^ & K. M. Ansari^1^**

*^1^Food Toxicology Laboratory, Food Drug and Chemical Toxicology Group, CSIR‐Indian Institute of Toxicology Research, Lucknow, India; ^2^Academy of Scientific and Innovative Research (AcSIR), CSIR‐Indian Institute of Toxicology Research Campus, Lucknow, Uttar Pradesh, India*

*Email:* kmansari@iitr.res.in

Patulin is a low molecular weight natural product, produced as secondary metabolite by *Penicillium, Aspergillus,* and *Byssochlamys sp*. of fungi. Patulin exerts its toxic effect by glutathione depletion, generation of ROS, oxidative DNA damage. Recently, it has been reported that Patulin increases colonic epithelial permeability, modulates tight junction and produced marked decrease in transepithelial electrical resistance. As DNA damaging and enhanced cell proliferation capability are the prerequisite for cell transformation and tumor growth, in the present study, we investigated whether low dose chronic exposure of patulin to intestinal cells *in vitro* and *in vivo* may leads neo‐plastic changes. In *in vitro* studies, rat normal intestinal epithelial cells (IEC‐6) were treated with non toxic dose (250 nmol/L) of patulin up to 16 weeks. We observed that compared to control, chronically exposed cells showed alteration in cellular morphology, increased cell migration and invasion capacity, increased MMPs activities and anchorage independent colony formation abilities. Further, to establish the physiological relevance of these *in vitro* findings, an *in‐vivo* study has been conducted, where low doses of patulin (25 *μ*g/kg and 100 *μ*g/kg body wt.) was orally given to rats up to 90 days. Here, we found that oral exposure of patulin caused significant appearance of colonic aberrant crypt foci as well as mucin depleted foci, which are putative biomarkers of pre‐neoplastic changes in rat colon. Thus, based on our *in vitro* and *in vivo* findings, we can suggest that low dose chronic exposure of patulin may lead to neo‐plastic changes in normal intestinal cells.

## Role of endoplasmic reticulum stress induced apoptosis in type 2 diabetes

**N. Tawar^1^, S. Gupta^2^, S. V. Madhu^3^ & B. D. Banerjee^1^**

*^1^Environmental Biochemistry and Molecular Biology Laboratory, Department of Biochemistry, UCMS and GTB Hospital; ^2^Department of Surgery, UCMS and GTB Hospital, University of Delhi, Delhi, India; ^3^Department of Medicine, UCMS and GTB Hospital, University of Delhi, Delhi, India*

*Email:* tawar.neha145@gmail.com

Endoplasmic reticulum (ER) stress, a common feature of several physiological and pathological conditions affecting the cellular functionality, triggers many rescuer responses, including unfolded protein response (UPR). UPR has a primary role in the stress adaptation and eventually the cell survival; however, under irreversible ER stress condition, damaged cells take switch to pro‐apoptotic signaling pathway through apoptosis. Increasing evidences from the literature have suggested that the ER stress has pivotal role in type 2 diabetes, but its mechanism in these patients is still poorly understood. The aim of the present study is to investigate the role of ER stress in type 2 diabetes. The visceral fat of type 2 diabetes (n = 25) and normal glucose tolerant human subjects (n = 25) was used for the study. The quantitative real time PCR for apoptotic (CCAAT/enhancer‐binding protein homologous protein, CHOP) and cell survival (Glucose regulated protein78) signaling pathways of ER stress was done to compare the differences in their gene expression. In our study, gene expression of CHOP, a pro‐apoptotic marker was elevated up to 3 folds and expression of Grp78, involved in UPR survival, was decreased to 2 folds in cases as compared to controls. The response to excessive aggregation of misfolded proteins (4 folds over expression of IRE gene) and translational attenuation was also observed (4 folds down regulation of PERK gene) in cases as compared to controls. The ER hosts a dynamic signaling network that sense and respond to physiologic changes that affect its environment, and hence influencing overall cell fate. UPR is classic inducers of ER resident signaling pathway. In this study we found the down regulation of genes involved in the translation activity (PERK) and upregulation of gene (IRE) occupied in misfolded protein accumulation in type2 diabetic patients as compared to controls. The downstream effector signals (CHOP and Grp78) are also altered in patients with type 2 diabetes, suggesting the involvement of ER stress response in this disease. The activated pro‐apoptotic pathway of ER stress might be involved in the pathophysiology of type 2 diabetes and hence can be targeted for new therapeutical approach.

## Role of Phosphoglycogen synthase kinase‐3*β* and *β*‐catenin in the pathobiology of urinary bladder cancer

**N. Maurya^1^, R. singh^1^, A. Goel^2^, A. Singhai^3^, U. P. Singh^4^, V. Agrawal^5^ & M. Garg^1^**

*^1^Department of Biochemistry, University of Lucknow, Lucknow, Uttar Pradesh, India; ^2^Department of Urology, King George's Medical University, Lucknow, Uttar Pradesh, India; ^3^Department of Pathology, King George's Medical University, Lucknow, Uttar Pradesh, India; ^4^Department of Urology, Sanjay Gandhi Post Graduate Institute of Medical Sciences, Lucknow, Uttar Pradesh, India; ^5^Department of Pathology, Sanjay Gandhi Post Graduate Institute of Medical Sciences, Lucknow, Uttar Pradesh, India*

*Email:* nihar.maurya@gmail.com

Glycogen synthase kinase‐3*β* (GSK‐3*β*) is a key regulator of many cellular functions including glycogen metabolism, differentiation, embryonic development, migration, apoptosis and survival. It is an essential component of Wnt/*β*‐catenin signal cascade where it sequesters and promotes the proteasomal degradation of *β*‐catenin. Aberrant activation of pS9GSK‐3*β* (phosphorylation at Serine 9) results in stabilization of *β*‐catenin and has been reported in cell proliferation, differentiation and increased aggressiveness in several major human cancers including pancreatic, colorectal, ovarian, prostate, cervical, breast and bladder cancer. This study aims to define the probable clinical significance of pS9GSK‐3*β* and *β*‐catenin in the pathobiology of urinary bladder cancer. Immunohistochemical (IHC) staining was done in a cohort of 90 patients diagnosed with urinary bladder cancer and ten normal bladder tissues from patients with benign prostate hyperplasia (BPH), to quantitatively examine the protein expression and cellular localization of pS9GSK‐3*β* and *β*‐catenin. *β*‐catenin was also analysed by Real Time‐quantitative Polymerase Chain Reaction (RT‐qPCR) to examine its expression at transcriptome level. IHC results were analysed and out of 90 patients, 46 tumors were shown to exhibit either loss of membranous pS9GSK‐3*β* or gain of cytosolic/ nuclear pS9GSK‐3*β* and were examined for significant association with pathological variables including low stage (*P* = 0.01, Mann‐Whitney test) and high grade (*P* = 0.04, Mann‐Whitney test) tumors. Out of 90, 63.3% (57/90) tumors were analysed to exhibit strong expression of total *β*–catenin at transcriptome level but did not exhibit any clinical significance. Further IHC was done on these tumors for *β*–catenin expression. Out of 90 cases, 44 tumors were identified for the presence of aberrant levels of *β*–catenin. Total of 28 tumors were found to show aberrant expressions of both pS9GSK‐3*β* and *β*–catenin. These tumors showed statistical significant association with low tumor stage (*P* = 0.01, Mann‐Whitney test), recurrence (*P* = 0.02, Mann‐Whitney test) and smoking status (*P* = 0.04, Mann‐Whitney test). Kaplan Meier's analysis along with the log rank test was done to identify their abnormal expression with shorter overall survival periods (*P* = 0.03). Significant association of aberrant expressions of pS9GSK‐3*β* and *β*–catenin in combination with non‐muscle invasiveness (low tumor stage), tumor recurrence and poor over survival of patients validates them as potential prognostic markers and strengthens their functions in the pathobiology of urinary bladder cancer.

## Role of phytoestrogens in inhibition of cervical carcinoma *via* modulating estrogen receptors

**N. Bano, A. Jafri, N. Shivnath, V. Rawat, J. Rais, Habiba, M. Tripathi & M. Arshad**

*Molecular Endocrinology Laboratory, Department of Zoology, University of Lucknow, Lucknow, Uttar Pradesh, India*

*Email:* nuzhatbano079@gmail.com

Cervical cancer is one of the deadliest but easily preventable cancers of women, responsible for more than 2,70,000 deaths annually, of which 85% occur in developing countries. In India cervical cancer is one of the leading causes of cancer mortality among women 30 to 69 years of age, accounting for 17% of all cancer deaths. Infection with oncogenic types of human papillomavirus (HPV) is a necessary, but not sufficient cause of cervical cancer. Epidemiological evidence strongly suggests that steroid hormones, primarily estrogens are implicated in cervical cancer. Estrogen promotes the development of cervical cancer in cells infected with high‐risk human papillomaviruses (HPVs). Recent studies provide evidence that estrogen and its nuclear receptors (i.e. ER*α* and ER*β*) promote cervical cancer in combination with HPV oncogenes (i.e. E6 and E7). Both estrogen and HPV oncoproteins regulate Eag1 potassium channel, of which inhibition leads to apoptosis of human cervical cancer cells. Chronic exposure to estrogen induces overexpression of Aurora‐A, a centrosome kinase, and centrosome amplification, leading to chromosomal instability. E6 and E7 each can inhibit cellular DNA damage responses, which in part may contribute to their promoting genomic instability. Phytoestrogens are non‐steroidal plant‐derived natural compounds basically similar to endogenous estrogens, but capable of displaying both estrogenic and anti‐estrogenic properties. Most phytoestrogens, however, are phenolic compounds of which the isoflavones and coumestans are the most widely researched groups. Phytoestrogens are a sub‐class of the polyphenols that have structural similarity to the endogenous hormone 17*β*‐estradiol and bind to estrogen receptors. Phytoestrogens acting as antiestrogens may prevent hormone‐related cancer (i.e. cervical) by blocking the proliferative effects of endogenous estrogens. Interestingly, these phytochemicals and their metabolites have been shown to be effective in preventing cervical disease in HPV transgenic mice. Further clinical studies are needed for the application of promising phytoestrogens for treatment of cervical cancer.

## Role of miR‐195 in mitochondrial dynamics in breast cancer

**P. K. Purohit & N. Saini**

*Functional Genomics Unit, CSIR – Institute of Genomics and Integrative Biology, Delhi, India*

*Email:* paresh.purohit@igib.res.in

Micro RNAs has emerged out as potential therapeutic molecules in various complex diseases including cancer. miRNA regulates gene expression post transcriptionally by binding to un‐translated region of mRNA. miR‐195 has been shown to play very important role in ontogenesis as its expression was significantly down‐regulated in breast tumor tissues as well as in breast cancer cell lines i.e. MDAMB‐231 and MCF‐7. We have shown previously that miR‐195 induces apoptosis by targeting BCL‐2; we have further shown that miR‐195 inhibits proliferation invasion and metastasis. All these properties suggest that miR‐195 is not just a pro‐apoptotic miRNA but it also inhibits aggressiveness of tumor. Pro‐apoptotic miRNA‐195 has also been reported to affect mitochondrial function by unbalancing mitochondrial calcium level and depolarizing mitochondria. Since mitochondria are very critical organelle of the cell so any defect in its function can lead to various pathophysiological diseases. The exact mechanism operated behind these miR‐195 mediated mitochondrial defects is not clear so far. So our objective is to unveil how miR‐195 is affecting mitochondrial function? To unveil miR‐195 mediated mitochondrial functional defect. *In silico* approached has been used to find out the direct target of miR‐195 involved in mitochondrial processes. To validate miR‐195 target western blotting and Luciferase assay will done. The reparatory defect induced by miR‐195 has been estimated using XF‐24 flux analyzer seahorse machine. Further to visualize mitochondrial morphology mitochondrial GFP was used and cells were visualized using Lieca SP8 microscope. *In‐silico* analysis of genes that are predicted to be targeted by miR‐195 revealed mitofusin‐2 as a direct target of candidate miRNA. Further down‐regulation of mfn2 upon over expression of miR‐195 in MDAMB‐231 and MCF‐7 cell line suggests that miR‐195 is targeting mfn‐2. Increased mitochondrial fragmentation upon over‐expression of miR‐195 suggests increased fission events. The OCR measurement using seahorse machine has also shown potential respiratory defect in mitochondria upon over expression of miR‐195 in both breast cancer cell lines. The respiratory defect induced by mir‐195 has to further investigate to know whether it is generalized effect of miR‐195 and that is due to increased fission or miR‐195 is specifically targeting component of electron transfer chain (ETC). Our preliminary result suggests that the miR‐195 is targeting mfn2 and blocking mitochondrial fusion and this can be a possible reason of increased mitochondrial stress and functional impairment. The study will help us to characterize miR‐195 as therapeutic molecules not just in cancer but various other diseases where mitochondrial health plays very important role such as neuro‐degeneration, myocardial infarction and diabetes.

## Association of aldehyde dehydrogenase with response to radiotherapy in head and neck squamous cell carcinoma

**P. Dubey, S. S. Singh, S. A. Malik, M. Asif, J. Miyan, R. Gupta, A. Mishra, V. Kumar, H. Ram, S. Bhadauria & M. L. B. Bhatt**

*Division of Toxicology and Experimental Medicine, Central Drug Research Institute, Lucknow, Uttar Pradesh, India*

*Email:* parul.dubey04@gmail.com

ALDH (aldehyde dehydrogenase) is an intracellular enzyme, normally present in liver. ALDH catalyzes the oxidation of retinol to retinoic acid. It plays a key role in cell proliferation, invasion and chemoresistance. It is also considered as one of the progenitor markers for cancer stem cells in various types of tumors. Its expression may help in predicting radiotherapy treatment response in head and neck squamous cell carcinoma (HNSCC) patients. The present study aims to study the association of ALDH with clinicopathological parameters (age, smoking/drinking history, clinical T‐stages) of HNSCC patients and their radiotherapy treatment response. Tissue specimens of 90 histopathologically confirmed HNSCC patients and 90 matched controls were recruited for the evaluation of ALDH by immunohistochemistry. The association between expression of ALDH, clinicopathological parameters, and radiotherapy treatment response of HNSCC was examined. All patients were recommended for radiotherapy and written informed consent was obtained. Study has been approved by Institutional Ethics Committee of KGMU Lucknow before the start of the study. The results of immunohistochemistry demonstrated significant increase in the protein expression of ALDH in HNSCC when compared with matched control tissues. The increased vulnerability of non‐responder patients involving clinicopathological parameters was also revealed. Final data provided a correlation of this protein with tumor size (*P* = 0.0431), lymph node (*P* = 0.003), grade (*P* = 0.0317), clinical T‐stages (*P* < 0.0001) and radiotherapy treatment response (*P* = 0.0088). No significant result was observed with respect to age and smoking/drinking history. Our analysis confirms the association of ALDH with radiotherapy response, lymph node metastasis, grade, clinical T‐stages and tumor size but no clear association was revealed with age and smoking/drinking history. Since in this regard, an attempt was made to assess the predictive significance of ALDH in determining radiotherapy response was fulfilled and thus it can be used as a predictive marker for HNSCC. The alterations in the protein profile of ALDH showing association with clinicopathological parameters and response to radiotherapy. Thus, the target protein could be used as potential marker for the radiotherapy treatment response.

## Circulating miRNA‐21 and 133a: non invasive biomarker in oral sub‐mucous fibrosis and oral squamous cell carcinoma

**P. singh^1^, A. N. Srivastava^1^, A. Singh^1^ & R. Sharma^2^**

*^1^Department of Pathology, Era's Lucknow Medical College and Hospital, Lucknow, Uttar Pradesh, India; ^2^Department of Biosciences, Integral University, Lucknow, Uttar Pradesh, India*

*Email:* poojaa.singh1989@gmail.com

Oral squamous cell carcinoma is associated with genetic and epigenetic regulations in expression of miRNA (miR). Recently, many studies on cancer focus on exploring detection of cancer using non invasive circulating biomarkers is going on. Circulating miRs may act as potential biomarkers for early diagnosis, treatment and prognosis. In the present study, an attempt has been made to see the expression level of miR‐21 and miR‐133a in serum of 10 OSMF, 10 OSCC cases and 20 healthy volunteers. The expression of miR‐21 was also evaluated in relation to demographical feature, pan‐masala with their aim to identify correlation with expression of miR‐21 in oral pre‐cancer and cancer cases. The relative expression level of miR‐21 and miR‐133a was determined by quantitative real‐time RT‐PCR (qRT‐PCR) in the sera of 10 OSMF, 10 OSCC patients and 20 healthy subjects as a control. Association between expression of miR‐21 and demographical parameter like pan‐masala have also been analyzed in detail. The t‐test results obtained revealed significant increase in the expression level of miR‐21 in OSCC as compared to OSMF. The study also revealed the positive correlation between higher miR‐21 expression and pan‐masala chewers as shown by t‐test. The miR‐133a has not attained its threshold ct value, hence its expression has been considered as down‐regulated in OSMF and OSCC as compared to healthy control. The results of the present study indicated up‐regulation of circulating miR‐21 in serum of OSCC as compared to OSMF (*P* = 0.001) and the study also elucidated the positive correlation between miR‐21 expression in OSCC/OSMF patients and the demographical parameter, pan‐masala. The result also revealed the decreased expression of miR‐133a in OSCC and OSMF as compared to healthy controls. More studies are needed to validate these miRNAs as novel diagnostic and prognostic biomarker for OSMF and OSCC for better management.

## Toxic effects of chronic nandrolone‐decanoate administration on the biomarkers of hepatotoxicity and oxidative stress in Swiss Mice

**P. K. Pandey, J. Dewangan, S. Mishra, S. Srivastava, A. Divakar & S. K. Rath**

*Genotoxicity Lab, Division of Toxicology and Experimental Medicine, CSIR‐Central Drug Research Institute, Lucknow, Uttar Pradesh, India*

*Email:* pandey.prabhash21@gmail.com

Anabolic‐androgenic steroids (AAS) are synthetic molecules similar to the male sex hormone testosterone. The classical therapeutic uses of these substances are the treatment of a number of diseases. Due to their anabolic and androgenic attribute, recreational athletes and bodybuilders are using AAS in an inappropriate and veiled manner with the aim of improving exercise performance or for cosmetic purposes. Individuals usually take doses 10 to 100 fold higher than the therapeutical dose; this abuse can cause many adverse effects. The aim of the present study is to evaluate the hepatotoxic potential of nandrolone‐decanoate (ND) through using the Swiss albino mice. Twenty‐four adult male Swiss mice were randomly assigned to the following groups: control, clinical, intermediate, and suprapharmacological doses of ND during 14 days. Biomarkers of hepatotoxicity and oxidative stress in Swiss Mice were measured in all studied groups. Serial sections of liver tissues were stained with haematoxylin and eosin for histological examination. The supraphysiological doses of ND results in pronounced hepatotoxicity as revealed by elevated level of serum biomarker enzymes. Furthermore, alterations in the activity of major antioxidant enzymes indicating the development of oxidative stress, was significant in using supraphysiological doses of ND. Degenerated liver tissues with histological alterations as compared to control were observed in supraphysiological doses of ND treated groups. The decrease in the level of antioxidant enzymes might be the consequence of decreased *de novo* synthesis of enzymes or irreversible inactivation of enzymes from increased free radical generation through ND metabolism. The alterations in activities of antioxidant enzymes of liver observed in the present study were an indication of oxidative injury brought by the ND dosing. The current observations clearly indicated that using supraphysiological doses of nandrolone‐decanoate can cause hepatotoxicity.

## Modulation of lipid profile and membrane fluidity by Acetyl‐11‐keto‐*β*‐boswellic acid in lung carcinogenesis induced by Benzo (a) pyrene

**P. Bhardwaj, M. L. Garg & D. K. Dhawan**

*Department of Biophysics, Punjab University, Chandigarh, Punjab, India*

*Email:* priti5biophysics@gmail.com

Membrane fluidity is the most important physiochemical property of cell membranes that can seriously affect functional properties of the cell and induction of apoptotic pathways resulting in cell death. The present study was designed to study the role of Acetyl‐11‐keto‐*β*‐boswellic acid (AK) on lipid profile and membrane fluidity in lung carcinogenesis conditions induced by Benzo(a)pyrene (BaP) in female SD rats. The animals were divided randomly into five groups which included (1) Normal Control, (2) Vehicle treated (olive oil), (3) BaP treated, (4) AK treated and (5) AK+ BaP (combined treated). BaP was administered at a dose of 50 mg/kg in olive oil twice a week orally for 4 weeks and AK (50 mg/kg) was given in olive oil thrice a week form 4 weeks prior to BaP exposure till 24th week. Total lipid content, levels of cholesterol, phospholipids, glycolipid and sphingolipids were estimated spectrophotometrically in lung tissue. Membrane fluidity was determined with DPH and Pyrene excimers using spectrofluorometer. Changes in membrane lipids were also analysed using FTIR. BaP treatment resulted in a significant (*P* ≤ 0.001) increase in membrane fluidity. Total lipid content and cholesterol levels were significantly decreased and levels of glycolipids and phospholipids were found to be significantly increased (*P* ≤ 0.001) in BaP treated rats were compared with normal control rats. Administration of AK in BaP treated animals significantly normalized the above indices towards normal controls. The present study suggests that the administration of AK reduces the BaP induced lung carcinogenesis and therefore can be advocated as a potential candidate for chemoprevention.

## Anti‐proliferative effect of *Datura innoxia* leaf extract on human lung adenocarcinoma A549 cells

**P. Agarwal, A. Jafri, M. Arshad & M. Serajuddin**

*Department of Zoology, University of Lucknow, Lucknow, Uttar Pradesh, India*

*Email:* priagar.87@gmail.com

*Daturainnoxia* Mill. is medicinally important plant species from the family Solanaceae, which is considered to be anti cancerous and may play important role in prevention and treatment of lung cancer because of availability of novel therapeutic agents. The current study aims to assess the anti‐proliferative activity of ethanolic leaf extract of *D. innoxia* (ELEDI) on human lung adenocarcinoma A549 cells. Leaves of *D. innoxia* were collected, cleaned, shade dried, powdered and then subjected to soxhlet extraction using ethanol as solvent. The A549 cells were treated with 25–500 *μ*g/mL concentrations of ELEDI for 24 h. The anti‐proliferative activity was evaluated by MTT (cell viability) and morphological study. Apoptotic effect was assessed by DAPI staining for nuclear fragmentation and reactive oxygen species (ROS) generation assay using DCFDA dye. Findings revealed that ELEDI significantly decrease the cell viability of A549 cells in a concentration dependent manner, whereas a significant fragmented nucleus were seen at increasing concentration of ELEDI. The successive increment in ROS was also observed at higher doses of ELEDI as compared to control. ELEDI showed a remarkable anti‐proliferative and apoptotic effect on A549 cells that was evidenced by significant decrease in cell viability, increased nuclear fragmentation and ROS mediated oxidative stress. The results of the present study showed that *D. innoxia* can be a potential candidate for anticancer drug. However, further research is warranted to find out its unexplored efficacy, mechanisms of its anticancer activity and novel bioactive compounds that could serve as scaffold in search of new drugs.

## Role of methyl‐CpG binding protein MBD2 in BRCA1 and p16 gene expression in MCF‐7 breast cancer cell line

**R. K. Sahu^1^, S. Tandon^2^, B. C. Das^2^, R. Mehrotra^3^ & S. T. Hedau^1^**

*^1^Division of Molecular Oncology, National Institute of Cancer Prevention and Research (ICMR), Noida, Uttar Pradesh, India; ^2^Amity Institute of Molecular Medicine & Stem Cell Research, Amity University, Noida, Uttar Pradesh, India; ^3^Division of Molecular Cytology, National Institute of Cancer Prevention and Research (ICMR), Noida, Uttar Pradesh, India*

*Email:* rkmicro18@gmail.com

Breast cancer is leading cause of cancer‐related death in women worldwide and showing rising trend, especially in urban India. BRCA1 gene is involved in DNA damage repair and p16 play important role in cell cycle regulation. How DNA methylation patterns are interpreted into different functional output remains poorly understood. One mechanism involves the ‘readers’ of methylation, which includes the methyl‐ CpG binding domain (MBD) family of proteins. These MBDs proteins have been genetically linked to disease in humans. The MBD family represents a group of proteins that generally act as mediators between methylation, primarily in the CpG context, and other chromatin and histone modifying protein complexes. The MBD protein family consists of MeCP2 and MBD1‐6. Despite the name, not all members of this family bind to methyl‐CpG with exclusively, or at all. Instead the MBD proteins have distinct DNA binding properties and other functional domains that may contribute to their respective functions. MeCP2, MBD1 and MBD2 bind to DNA in a methyl‐CpG density dependent manner via the MBD and associate with co‐repressor and other protein complexes through their transcriptional repression domains. This study's aim was to identify MBD2 as biomarker which regulate the BRCA1 and p16 gene expression in MCF‐7 breast cancer cells by Real Time PCR. MCF‐7 cells were maintained in DMEM media and 30 *μ*mol/L IC50 of resveratrol calculated by MTT assay. Different concentration of resveratrol (*μ*mol/L) treated in MCF‐7 cells. Then RNA extracted and cDNA prepared followed by Real Time PCR was performed. Statistical analysis was done using one way ANOVA. Real time results showed that MBD2 gene expression decreases as resveratrol conc. Increases. BRCA1 mRNA expression level increased but p16 mRNA expression decreased in higher concentration of resveratrol treated MCF‐7 cells. Real time quantitative results showed that significantly altered gene expression in all three genes treated with higher concentration of resveratrol as compare to control. MBD2 gene expression (*P *< 0.0001, *r* ^2^ = 0.6685)decreases up to 40uM of resveratrol and negatively regulate BRCA1 expression (*P *< 0.0001, *r* ^2^ = 0.6663) however in p16 (*P* < 0.0001, *r* ^2^ = 0.9418) it shows positive effect. MBD2 has regulatory role in BRCA1 gene expression and could be used as biomarker for breast cancer and targeted by drug combination therapy.

## Altered nucleotide excision repair mechanism and elevated DNA damage in bladder cancer patients: a case‐control study

**R. Ghanshela, S. Gupta & B. D. Banerjee**

*Environmental Biochemistry and Molecular Biology lab, Department of Biochemistry, Department of Surgery, UCMS and GTB Hospital, University of Delhi, Dilshad Garden, Delhi, India*

*Email:* rasshmi2@gmail.com

Bladder cancer (BC) is the most common malignancy of the urinary system. Exposure to genotoxic agents could result in DNA damage resulting in genetic instability and eventually carcinogenesis. Numerous DNA repair pathways are involved in the repair of DNA damage. One such pathway is Nucleotide Excision Repair (NER). Any alteration in NER genes could result in increased accumulation of DNA damage and increase an individual's susceptibility to BC development. The aim of the present study is to investigate the relationship between NER (XPA and XPG) gene expression and extent of DNA damage in bladder cancer patients. Our study included 35 BC patients and 35 age‐matched healthy controls. XPA and XPG gene expression was determined by quantitative real‐time PCR. DNA damage was assessed by comet assay. We have observed significantly higher levels of DNA damage in cases compared to controls. Also, the expression of both the NER genes was found to be significantly down‐regulated in BC cases compared to healthy controls. XPA gene was 3.23 folds down‐regulated while XPG gene was 3.03 folds down‐regulated in BC patients. Moreover, a significant correlation between extent of DNA damage and gene expression was found. Our observations indicate that down‐regulation of XPA and XPG genes increased the accumulation of DNA damage and might be linked with BC susceptibility. Besides, this study shows that the interaction between increased DNA damage levels and down‐regulated DNA repair genes might magnify the risk of BC & can be a causal factor in the aetiology of BC. However, studies with larger sample size are warranted for further corroboration. The presence of varied DNA repair efficiency because of altered gene expression may be the cause of an increased DNA damage in UBC patients making them more susceptible to developing cancer as compared to controls.

## SWI/SNF complex and Notch signalling pathway in apoptosis of Drosophila larval neural stem cells

**R. Kumar^1^ & R. Joshi**

*^1^Lab of Drosophila Neural Development, Centre for DNA Fingerprinting and Diagnostics, Hyderabad, Karnataka, India*

*Email:* raviranjanibb@gmail.com

The SWI/SNF chromatin‐remodeling complex is important for animal development. Mutations in the components of SWI/SNF lead to cancer. One of the characteristic hallmark of cancer includes resistance to cell death. The mechanism of apoptotic resistance is poorly understood. We have chosen the larval neural stem cells in *Drosophila* as a model to understand the same. To identify the regulator of programmed cell death in larval neural stem cells in *Drosophila* we performed knock down for 120 genes using a neural stem cell specific driver. We have found the role of epigenetic regulation in programmed cell death as well as in differentiation of neural stem cells. We find that SWI/SNF complex members are important for apoptosis of larval neural stem cells in *Drosophila*. Notch pathway plays crucial role in organism development. Our genetic experiments with SWI/SNF complex and Notch further confirm that they work together to controlling the apoptosis of larval neural stem cells. We further observe that SWI/SNF complex and Notch signaling are important for maintenance of the apoptotic enhancer, which in turn is important for activating the downstream apoptotic genes. Taken together, our results indicate that Brm and Notch together control apoptosis by regulating the activity of the apoptotic enhancer in the *Drosophila* larval CNS.

## High grade intraepithelial Neoplasia – a useful tool as a precursor of prostate carcinoma

**S. S. Haider, T. Munsif, S. K. Bhatt & P. Singh**

*Department of Anatomy, Amity University, Lucknow, Uttar Pradesh, India*

*Email:* sshaider241270@gmail.com

Prostatic Intraepithelial neoplasia (PIN), particularly high grade PIN (HGPIN), & atypical small acinar proliferation (ASAP) have been identified as precursor lesions to prostatic carcinoma. PIN refers to the precancerous end of a morphologic spectrum involving cellular proliferation within prostatic ducts, ductules & acini. The incidence of high grade PIN on biopsy ranges from 1.5 to 6.5% with an average of 6%. HGPIN is the most likely precursor of prostatic carcinoma. When HGPIN is identified pathologist carefully searches tissue specimens for evidence of cancer. Patients in whom HGPIN is found are usually advised by urologist to begin continued follow‐up care with serum prostate specific antigen (PSA) testing, physical examination, and repeat biopsies. An estimated 30% of men with HGPIN develop clinical evidence of prostate cancer within one year. The present study has been performed on 100 patients in the department of Surgery, Eras Lucknow medical college and hospital. The age of the patients varied from 40–80 years. Maximum proportions of patients (34%) were aged between 60–69 years followed by 70–79 years (30%). There were 12% patients in age group 40–49 years and 24% were in the age group of 50–59 years. Thus it was seen that the complaints of lower urinary tract system or prostatic disease were common in higher age group. In this study all the precancerous lesions were more common in the higher age groups (60–69 and 70–79 years). However, no statistically significant association between type of lesion and age could be established in this study (*P* = 0.267). In our study out of 11 cases with serum PSA levels above 4 ng/mL, three were diagnosed to be having High grade Prostatic Intraepithelial Neoplasia, thus indicating the positive predictive value of serum PSA to be 27.3%. Out of total 5 cases diagnosed as HGPIN, the serum PSA levels were able to diagnose 3, thus indicating a sensitivity of 60%.

## Cell signaling is synchronized in entotic and entytic cells upon extracellular stimulation

**S. Chourasia, I. Ahmad & D. K. Singh**

*Immunotoxicology Division, Systems Toxicology and Health Risk Assessment Group, CSIR‐Indian Institute of Toxicology Research (CSIR‐IITR), Lucknow, Uttar Pradesh, India*

*Email:* sabita.chourasia@gmail.com

Entosis is non‐apoptotic cell death process that occurs by the invasion of a living cell into the cytoplasm of the other. Modern molecular biology state‐of‐the‐art ‐omics tools like genomics, proteomics, transcriptomics, metabolomics is of little to no value in the study of cell signaling during entosis. We used high‐throughput imaging tools to study the kinetics of cell signaling during entosis in breast cancer cell line MCF‐7 in response to proliferating and anti‐proliferating stimuli. Specifically, we studied the kinetics of cellular signaling in response to both proliferating (EGF and Insulin) and inflammatory stimuli (TNF*α*) using two pairs of phospho‐antibodies against active signaling molecules common to both arm of the stimuli. Surprisingly, we observed that the synchronized intracellular signaling in entotic and entytic cells upon cell surface receptor stimulation (EGFR, Insulin Receptor, TNF*α* receptor) of the entotic cells. Our result suggests that an entytic cell residing within the entotic cell is capable of sensing the receptor stimulation of later by an unknown mechanism that warrants detailed molecular investigation.

## Survivin inhibition limits TGF*β*‐induced fibrogenic response of hepatic stellate cells

**S. Sharma, S. M. Ghufran & S. Biswas**

*Amity Institute of Molecular Medicine and Stem Cell Research (AIMMSCR), Amity University, Noida, Uttar Pradesh, India*

*Email:* sachin.research@hotmail.com

Activation of Hepatic Stellate Cells (HSCs) is the key initiating event in hepatic fibrogenesis of damaged liver characterized by secretion of extra‐cellular matrix (ECM) proteins such as collagen, fibronectin. Transforming Growth Factor *β*1 (TGF*β*1), most potent pro‐fibrotic cytokine responsible for the activation of HSCs and induces the secretion of ECM proteins. Here, we are investigating the role of survivin protein in HSCs activation and fibrogenic response using its pharmacological inhibitor YM155. Human HSCs, LX2 cells were treated with TGF*β*1 with or without YM155 for 24 h. The viability of cells was measured by MTT assay and with annexin‐propidium iodide (PI) staining using flow cytometry. We have analyzed the HSCs activation using specific markers of *α*‐SMA, vimentin. The gene expressions of ECM related genes, COL1A1, COL4A1 and fibronectin were quantified through qPCR. Effect of survivin inhibition on HSCs were evaluated with cell migration and contraction ability assays. Here we have shown that the fibrogenic responses of LX2 were induced with TGF*β*1 treatment having significant upregulation of survivin, HSCs activation marker *α*‐SMA, vimentin and ECM related genes COL1A1, COL4A1, fibronectin etc. To understand the effect YM155 on TGF*β*1 activated LX2 cells, we first measured the IC50 value of 55 nmol/L upon treatment of different concentration of YM155 on LX2 cells for 24 h. Here we found that YM155 treatment clearly reduced the fibrogenic responses of LX2 cells with decreasing expression of survivin, *α*‐SMA, vimentin, COL1A1, COL4A1, fibronectin in HSCs. There were also significant reduction of cell migration and contraction ability of TGF*β*1 activated HSCs upon treatment of YM155, implies the inhibitory effect of survivin limits the fibrogenic responses of stellate cells. We concluded that TGF*β*1 induces the expression of survivin along with activation of HSCs. Targeting the hepatic stellate cells with pharmacological inhibitor of survivin is a promising anti‐fibrotic approach in liver diseases.

## Antiproliferative efficacy of curcumin mimics through microtubule destabilization

**S. Khwaja^1^, K. Fatima^1^, Hassanain^2^, C. Behera^3^, A. Kour^3^, A. Singh^1^, N. Pathak^1^, S. Luqman^1^, J. Sarkar^2^, D. Chanda^1^, K. Shanker^1^, A. K. Gupta^1^, D. M. Mondhe^3^ & A. S. Negi^1^**

*^1^CSIR‐Central Institute of Medicinal and Aromatic Plants, Lucknow, Uttar Pradesh, India; ^2^CSIR‐Central Drug Research Institute, Lucknow, Uttar Pradesh, India; ^3^CSIR‐Indian Institute of Integrative Medicine, Jammu, Jammu and Kashmir, India*

*Email:* sadiyakhwaja89@gmail.com

Cancer has been a challenge for public healthcare sector because of its leading cause of morbidity and mortality. The economic impact of cancer is significant and is increasing. Curcumin is a phenolic compound isolated from the rhizome of *Curcuma longa* L. (Zingiberaceae). Three curcuminoids of major occurrence are curcumin, demethoxycurcumin, and bisdemethoxycurcumin. Curcumin possesses an attractive chemical structure with highly conjugated diferuloylmethane. Curcumin mimics have been designed and prepared with an additional bridged phenyl ring in conjugation for better efficacy. Fourteen diverse analogues were prepared through chemical synthesis and evaluated for detailed pharmacology as possible anticancer agents. The best analogue, 6a exhibited potent cytotoxicity against A431, epidermoid carcinoma cell line (IC50 = 1.5 *μ*mol/L) and DLD1, colorectal adenocarcinoma (IC50 = 6.9 *μ*mol/L). 6a destabilized tubulin polymerization process (IC50 = 4.68 *μ*mol/L), exerted G2/M phase arrest and induced apoptosis. In Ehrlich Ascites Carcinoma in Swiss‐albino mice, 6a showed 78.6% tumour reduction at 80 mg/kg dose. It was well tolerable up to 300 mg/kg dose in acute oral toxicity. Curcumin depolymerizes interphase microtubules at 25 *μ*mol/L and 40 *μ*mol/L concentrations. Confocal microscopy showed destabilization of microtubules clearly at 20 *μ*mol/L concentration of 6a. Interference in microtubule dynamics disturbs mitosis and such compounds cause G2/M phase arrest. Induction of cell cycle arrest in cancer cells constitutes one of the most prevalent strategies to stop or limit cancer spreading. Ehrlich Ascites Carcinoma is known as undifferentiated carcinoma which is more malignant than a differentiated carcinoma and difficult to treat. The present study shows that the novel curcumin mimic 6a is safe and efficacious anticancer compound. However, it needs to be optimized for better activity.

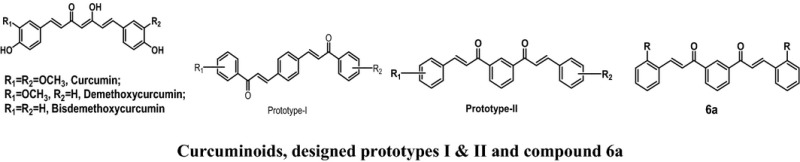

## Effect of cypermethrin nanoparticles on freshwater murrel, *Channa punctatus*

**S. Amjad, G. Kumar, A. K. Sharma & M. Serajuddin**

*Department of Zoology, University of Lucknow, Lucknow, Uttar Pradesh, India*

*Email:* saimaamjadlko@gmail.com, lu.fisheries@gmail.com

Nanotechnology has made a rapid progress in the fields of environmental protection, hygiene, therapy, and agriculture. The recent application of nanotechnology in agriculture is responsible for the effective delivery of pesticide active ingredients in the form of nanopesticides. The current level of knowledge is considered to be not enough for the understanding of the fate assessment of the use of nanopesticides. Therefore it is considered to be essential to evaluate the toxicity of nanopesticides before their application in order to preserve the quality of both the food chain and the aquatic environment. The aim of this study was to determine the effects of cypermethrin nanoparticles on *Channa punctatus*, and to establish whether the nanoformulation of cypermethrin was less toxic for *C. punctatus* than the common formulation. The animals were administered with different doses of 1/10 and 1/20 of 96 h LC50 value of cypermethrin and cypermethrin nanoparticles for 15 days. Biochemical analysis of blood serum was evaluated by Alanine aminotransferase (ALT), aspartate aminotransferase (AST) and alkaline aminotransferase (ALP) biomarker. Significant increased in ALT, AST and ALP of blood serum on the exposure of both 1/10 and 1/20 of 96 h LC50 was noted with cypermethrin and cypermethrin nanoparticles. However, the severity of these effects was less due to cypermethrin nanoparticles compared to the common preparation. The high level of ALT was recorded in response of different doses of cypermethrin and cypermethrin nanoparticles which indicated highly stressed condition in *C. punctatus*. The significant elevation in ALP and AST were recorded at the end of the exposure period indicated highly damaged liver in the exposed fish. Our study indicated that the nanoformulation of insecticides may be safer to apply to reducing the environmental and human adverse effects. The results of this study suggest that the presence of cypermethrin nanoformulation may cause low harmful effect on aquatic organisms. This study will be very helpful for the assessment of ecotoxicological risks of cypermethrin nanoparticles.

## Role of uncharacterized proteins in organ development and functions

**S. Fatma, R. Kumar, A. Dixit & R. K. Swain**

*Institute of Life Sciences, Nalco square, Bhubaneswar, Odisha, India*

*Email:* fatmasana123@gmail.com

Advances in computational biology have led to prediction of protein coding genes with high degree of accuracy. The proteins which do not have a homologue in protein database with identified function are classified as uncharacterized proteins. They comprise a substantial fraction of prokaryotic and eukaryotic genomes. Some of them are conserved among organisms. Many computational tools are available for predicting their function, but this give only limited information. Zebrafish has emerged as a major vertebrate model organism due to its high sequence identity to human genes. Expression analysis of uncharacterized genes in zebrafish embryos and generation of knockout fishes may provide a way to predict their function. A computational approach has been made to assemble uncharacterized proteins present in human and other vertebrates frequently used for modelling human disease biology such as mice, chicken, xenopus, and zebrafish. List of uncharacterized proteins which are present in zebrafish was compiled. The protein was said conserved if it has homologue in three species. mRNA expression analysis were done in zebrafish embryos by doing whole mount in situ hybridization. Further characterization of the genes which are showing organ specific expression will be done by using CRISPR/Cas or TALEN. To identify their interacting partner yeast two hybrid screening and pull down assay will be done. mRNA expression analysis of uncharacterized proteins in zebrafish embryos showed that most of them have ubiquitous expression pattern. Some of the genes are showing organs specific expression, this suggests that these genes may have role in the development and function of these organs. During expression analysis we identified two genes which are expressing in zebrafish pronephros. The functional characterization of uncharacterized proteins will help us to expand our knowledge related to organs development, physiology, disease and drug response. Because of the simplicity zebrafish pronephros emerges as a relevant nephrogenesis model. The structural organization of nephros and its function is associated with kidney health, and defects in it cause disease condition which may lead to renal cancer. The functional characterization of uncharacterized genes expressing in zebrafish pronephros may help in the identification of new molecular pathway associated with kidney development and function. We have identified two uncharacterized genes which may have role in kidney development and function. Our proposed work may lead to the identification of new biomarkers.

## Multimodal lyotropic liquid crystalline nanoparticles with aggregation‐induced effect for image‐guided cancer chemotherapy

**S. Urandur, V. T. Banala, S. Sharma, R. P. Shukla & P. R. Mishra**

*CSIR‐Central Drug Research Institute, Lucknow, Uttar Pradesh, India*

*Email:* sandeep.urandur@gmail.com

Drug delivery systems encompassed with imaging properties has unwrapped new avenues for the development of cancer theranostics. Hexosomes are colloidal dispersions of an inverted type of hexagonal phase lyotropic liquid crystalline particles they have been receiving ascending interest in pharmaceutical applications owing to their physical stability and high payload over liposomes. For non‐invasive bioimaging purpose biocompatible organic fluorophores with aggregation induced emission property (AIE) are better alternative than the inorganic fluorophores and to lower toxicity of cancer treatment ligand based targeted therapy will help us to improve the therapeutic efficacy. The present study aims for development of Anisamide (AA) anchored multifunctional lyotropic liquid crystalline particles ensembled with Formononetin (FMN), a phytoestrogen with known anticancer activity and Tetraphenylethene (TPE), an iconic optical beacon with AIE effect for simultaneous tumor imaging and therapy. Two formulations such as Anisamide conjugated hexosomes (HEX‐AA) and non conjugated hexosomes (HEX) were doped with FMN and TPE. Further, the physicochemical characterization was carried to determine the size and structural integrity of the nanoparticles. NMR studies were carried out to confirm the conjugation of AA to the hexosomes. In vitro studies like cellular cytotoxicity, apoptosis and cell cycle arrest were carried out in MDA‐MB 231 and 4T1 cancer cell lines. In vivo optical imaging and in vivo anti‐tumor efficacy were also performed in 4T1 breast carcinoma bearing mice. Ordered 3D mesoporous internal structure and high lipid volume fraction of hexosomes frame the outer compartment for the better settlement of payloads. SAXS, and electron microscopic techniques which were used in this study confirmed the inverse hexagonal phase of the nanoparticles. Embellishment of nanoparticles by AA, using carbodiimide coupling chemistry ensured hexosomes as an outstanding vehicle for the surveillance of tumor location as well as FMN delivery through active AIE imaging. The existence of AIE effect in the nanoparticles was evidenced through the photophysical studies that advocate the application of HEX‐AA NPs in fluorescence‐based bioimaging. Moreover, confocal microscopy illustrated the single living cell imaging ability endowed by the HEX‐AA NPs. In vitro and in vivo studies supported the enhanced efficacy of targeted nanoparticles (HEX‐AA) in comparison to non‐targeted nanoparticles (HEX) and free drug. These findings suggests that HEX‐AA NPs elicited the dual function of targeted tumor delivery and tracing the delivery of FMN with strategic use of TPE, which could provide a plausible platform for image‐guided diagnosis and treatment simultaneously in breast cancer.

## Investigating the patulin mycotoxin induced renal toxicity in Wistar rats

**S. Pal^1,2^, N. Singh^1,2^, M. Bansal^1,2^, S. Srivastava^1^, I. Dev^1,2^, A. Manoj^1^ & K. M. Ansari^1,2^**

*^1^Food Toxicology Laboratory, Food Drug and Chemical Toxicology Group, CSIR‐Indian Institute of Toxicology Research, Lucknow, India; ^2^Academy of Scientific and Innovative Research (AcSIR), CSIR‐Indian Institute of Toxicology Research Campus, Lucknow, Uttar Pradesh, India*

*Email:* srvpal888@gmail.com

Patulin (PAT) is a secondary metabolite produced by Aspergillus and Penicillium fungal species. The leading source of PAT is apples and apple juices made from infected fruits. Chemically it is a polyketide lactone (C7H6O4), and heat stable mycotoxin, not destroyed by the pasteurization during recent surveillance studies. PAT has been reported one of the major fruit juices contaminants in India. Thus systemic exposure of PAT would directly affect the kidney as one of its target organs. Since kidney plays an obligatory role in excretion, reabsorption, and general homeostasis. In the current study, we evaluated the renal toxicity after PAT exposure using the rat as an animal model. Adult rats were exposed to PAT for 7, 14 and 28 days through gavage at 25 and 100 *μ*g/kg of body weight. At the end of the experiment, animals were sacrificed and kidney tissues were collected, immediately weight, perfused and fixed in formalin. A novel method was developed to detect the presence of PAT in the urine of treated animals by using UPLC. Acute renal injury biomarkers (viz. Kim‐1, TIMP‐1, Clusterin and Calbindin, Lipocalin, VEGF, Osteopontin) along serum albumin, creatinine, and uric acid were measured in urine and serum samples. Kim‐1, TIMP‐1, Clusterin, and Calbindin, VEGF were found to be significantly up‐regulated in 7 and 14 days treatment groups. Similarly, creatinine, urea, and uric acid were also found to enhance significantly. The acute renal biomarkers were further confirmed by immunoblotting. Moreover, the histopathological analysis indicated that PAT exposure showed changes in renal tubules and glomeruli along tissue hyalinization Overall, our findings suggest that dietary exposure of PAT may lead renal toxicity.

## Cytotoxic action of 3‐bromopyruvate against tumor cells of thymoma origin: implication of diverse molecular mechanisms

**S. Yadav^1^, S. K. Pandey^1^, A. Kumar^2^, P. K. Kujur^3^, R. P. Singh^3^ & S. M. Singh^1^**

*^1^School of Biotechnology, Institute of Science, Banaras Hindu University, Varanasi, Uttar Prades, India; ^2^Department of Zoology, Institute of Science, Banaras Hindu University, Varanasi, Uttar Prades, India; ^3^School of Life Sciences, Jawaharlal Nehru University, New Delhi, India*

*Email:* savegdv09@gmail.com

Bromopyruvate (3‐BP), a brominated derivative of pyruvate, exerts a broad range of antineoplastic actions. As there is no report regarding the antitumor action of 3‐BP against malignancies of thymus, we investigated its antineoplastic action against cells of a murine transplantable lymphoma of thymoma origin. Antitumor efficacy of 3‐BP was investigated to appraise its therapeutic potential and underlying molecular mechanisms. Tumor cells were treated with 3‐BP in vitro followed by estimation of cell survival by MTT assay and trypan blue dye exclusion test. Cell death was determined by Wright‐Giemsa staining using light microscopy and Annexin‐V/PI staining using fluorescence microscopy and flowcytometric analysis. Expression of genes, proteins and cytokines were evaluated by RT‐PCR, Western blotting and ELISA respectively. Production of reactive oxygen species (ROS) was examined by fluorescence of indicator dye and intracellular pH by spectrofluorometric method. Glucose and lactate contents were determined by biochemical methods. Tumor cells treated with 3‐BP displayed inhibition of survival by induction of apoptosis and necrosis. It also altered expression of metabolism, chemosensitivity and cell survival regulatory molecules. Pre‐treatment with MCT‐1 inhibitor *α*‐cyano‐4‐hydroxycinnamate and siRNA gene silencing of HK 2 implicated the role of MCT‐1 and HK 2 in 3‐BP cytotoxicity. 3‐BP also altered expression of cell death regulatory Bcl‐2, Mcl‐1, caspase‐3 accompanied by increased cytochrome c release, indicating mitochondrial mode of cell death. Mechanisms of 3‐BP triggered tumor cell death indicate the involvement of altered pH homeostasis, inhibited target enzymes, modulated metabolism, and inhibited HIF‐1*α* expression, modulated cytokines & mediators of oxidative stress, tumor microenvironment and regulators of cell death and chemosensitivity. The study collates possible molecular mechanisms of cytotoxic action of 3‐BP, which will help to optimize the therapeutic efficacy of 3‐BP against tumors of thymic origin.

## Therapeutic role of nanotized phytochemicals in cervical cancer

**S. Parveen, N. Yadav, S. Kumar & M. Banerjee**

*Molecular & Human Genetics Laboratory, Department of Zoology, University of Lucknow, Lucknow, Uttar Pradesh, India*

*Email:* shamaparveen1192@gmail.com, mhglucknow@yahoo.com

Cervical cancer is the second most common cancer among women in the developing world, and the largest cancer killer among women in most developing countries, including India. Phytochemicals and their derived metabolites present in root, leaf, flower, stem and bark affect human systems. These elements or their altered forms have shown significant antitumor potential. Phytochemicals play a role in inhibiting tumorogenesis in cancer cells through the antioxidant, anti‐inflammatory, anti‐proliferative and pro‐apoptotic mechanism. Quercetin is one of the major dietary polyphenol found in several fruits vegetable and beverages. Quercetin downregulates Bcl‐2 through inhibition of NF‐kB and phosphorylation of EGFR thus, suppressing downstream signaling in cervical carcinoma cells. Myricetin is one of the major phytochemicals present in onions and berries and has been found to inhibit angiogenesis *via* the inhibition of PI3K and the suppression of matrix metalloproteinase responsible for vascular growth. Apigenin is another abundant flavonoid found in onions and induces apoptosis in cervical adenocarcinoma cells. Similarly, sulforaphane found in cruciferous vegetables has been demonstrated to trigger cell cycle arrest in cervical cancer cells. Camptothecin and its analogs induce the apoptosis of cancer cells by inhibiting DNA topoisomerase I. Nanotechnology is the approach to manufacture nanomaterials at the atomic and molecular levels. It has improved the management and appears as a promising technique for treating cancers. Nano‐phytochemicals inhibit cell proliferation, tumorogenesis and induce cell cycle arrest *via* different signaling pathways. In cervical cancer, nanotechnology has been increasingly examined to enhance early diagnosis and improve treatment efficacy. Use of nanophytochemicals as nanomedicine may help overcome the barrier to drug delivery and achieve higher efficacy in cervical cancer treatment.

## NLGP rescue TGF*β*‐mediated switching of pro‐ to‐anti apoptotic signaling of RGS5 to normalize pericytes in tumor

**S. Dasgupta^1^, T. Ghosh^1^, J. Dhar^2^, I. Guha^1^, A. Saha^1^, P. N. S. Majumdar^2^, P. Chakrabarti^2^, R. Baral^1^ & A. Bose^1^**

*^1^Department of Immunoregulation and Immunodiagnostics, Chittaranjan National Cancer Institute, Kolkata, India; ^2^Bose Institute, Kolkata, India*

*Email:* shayani.dasgupta7@gmail.com

Interactions between tumor cells and stromal cells modify the functions and phenotype of stromal cells to facilitate tumor progression. Among the various populations of tumor‐residing non‐hematopoietic stromal cells (NHSCs) functionally altered pericytes (cells wrapping blood capillaries) show strong expression of pro‐apoptotic molecule, ‘regulator of G‐protein Signaling 5′ (RGS5) which may confer its immunoregulatory phenotypes. However, the frequency of RGS5^high^ pericytes within tumor increased with disease progression, signifying the presence of altered cell death pathway within tumor microenvironment (TME).To decipher how despite the presence of pro‐apoptotic/lethal RGS5, tumor pericytes ensure its survival within tumor and to elucidate its mechanism. Pericytes were either isolated from tumor and non‐tumor organs by flow‐sorting or *in vitro* generated from C3H10T1/2 using TGF*β*/PDGF. Differential RGS5 expression was induced by using RGS5‐expressing plasmid‐vector, Thiazolidinedione, RGS5‐specific Si‐RNA. Apoptosis was measured using Annexin V‐PI staining. RT‐PCR, Western blot, and flow cytometry were used to study the various downstream molecules responsible for classical and alternate apoptotic RGS5 signature. Binding of RGS5 with either Galpha or Smad proteins were studied by co‐immunoprecipitation followed by western‐blotting. Bioinformatic analysis and ChIP assay were used to study the binding of Smads and RGS5 with Smad Binding Element (SBE) in upstream of bik promoter. Induced‐RGS5 expression promote apoptosis, by G*α*i activation and terminate PI3K‐AKT mediated survival signaling by downregulating Bcl2 and initiating PUMA‐P53‐Bax mediated mitochondrial damage. However, TGF*β* within TME inhibits binding of RGS5 with G*α*i and rescue RGS5‐mediated termination of survival signaling. Exposure of TME promotes binding of RGS5 with Smad2 and rapid nuclear translocation of RGS5 to initiate its alternate functions. NLGP treatment significantly modifies the TME by down‐regulating TGF*β* to ensure functional normalization of tumor‐pericytes as well as death of altered pericytes. We are reporting the classical pro‐apoptotic RGS5 signaling in normal‐pericytes and how TGF*β* within TME switch this classical signaling to alternate signaling by promoting crosstalk between RGS5 and TGF*β*‐mediator Smads which eventually alter the cellular distribution of RGS5. NLGP therapy downregulates TGF*β* without affecting RGS5 in pericytes which support sustained tumor growth restriction. Our study reporting a novel mechanism where inflammatory TME alters the RGS5‐mediated classical cell death signaling in pericytes to ensure its survival and tumor promoting immunoregulatory functions

## Isothymusin, a natural polyphenolic constituent as a promising inhibitor of cancer cell proliferation

**S. Singh, D. Kanojiya, P. Kumari, S. Luqman & A. Meena**

*Molecular Bioprospection Department and Academy of Scientific & Innovative Research, CSIR‐Central Institute of Medicinal and Aromatic Plants, Lucknow, Uttar Pradesh, India*

*Email:* a.meena@cimap.res.in

The plant‐based molecules as an anticancer agent are known for centuries in both traditional and contemporary medicine. In the present treatment era, the toxicity and non‐targeted nature of many anticancer drugs led to the search of novel molecules with potent anticancer effect. We have identified some of the molecules that retard the growth of cancer cells by modulating various mechanisms including proliferation, differentiation, apoptosis, angiogenesis, and metastasis. Isothymusin (6,7‐Dimethoxy‐5,8,4′‐trihydroxyflavone), a polyphenolic constituent present in *Ocimum sanctum*,* Limnophilla geoffrayi*,* Becium grandiflorum* is one of the promising molecule that target carcinogenesis process both at cellular and molecular level. We have explored the antiproliferative potential of Isothymusin and its possible mechanism of action by studying various targets (ornithine decarboxylase, cathepsin D, hyaluronidase, dihydrofolate reductase, cyclooxygenase‐2 and lipoxygenase‐5) involved in initiation, promotion and progression of cancer cell. The relationship with redox control of cellular events was also investigated by determining the radical scavenging and antioxidant properties of the molecules. The antiproliferative potential of Isothymusin was measured by performing MTT, SRB and NRU assays in different human cancer cell lines. The inhibitory effect against selected targets was measured using established colorimetric method. *In silico* studies were done to find out receptor‐ligand binding interaction, toxicity analysis and ADMET profiling. Isothymusin showed antiproliferative activity (IC50 24.44–71.71 *μ*g/mL) against leukaemia, colon skin and breast cancer cell lines. The proliferation of other cell lines (HaCaT, PC‐3, HEK‐293, A‐549, WRL‐68, NCIH‐520, MCF‐7) was decreased up to 48.18% at a tested concentration of 100 *μ*mol/L. Isothymusin inhibited cathepsin D (44.53 ± 2.54%), ornithine decarboxylase (37.67 ± 1.67%), dihydrofolate reductase (16.95 ± 1.03%), hyaluronidase (18.73 ± 1.28%), cyclooxygenase‐2 (22.22 ± 2.75%) and lipoxygenase‐5 (16.92 ± 0.59%) activity. It also scavenges the formation of DPPH radical (57.67 ± 1.5%) with moderate ferric reducing ability (41.97 ± 0.70 FSE) and reducing potential (0.58 ± 0.00). The production of the nitric oxide was also restrained by 47.72 ± 0.72%. *In silico* analysis revealed a strong binding interaction (binding energy ranged from −6.6 to −8.8 kcal/mol) of Isothymusin with the tested receptors/enzymes. The toxicity prediction of Isothymusin implies that it is non‐carcinogenic, non‐mutagenic, non‐skin irritant and moderate ocular irritant. The 4′ hydroxy in ring C, 5 hydroxy ring A and conjugated double bond in ring B might be responsible for the antiproliferative activity of Isothymusin and the free 4′‐hydroxyl group and p‐hydroquinone nature of the A ring possibly contribute towards the reducing power and scavenging potential. Isothymusin revealed antiproliferative, radical scavenging and reducing potential and could be taken up as a lead for further derivatization to prepare better analogues with improved activity.

## Vitamin D and gallic acid combination induces PI3K/Akt mediated apoptosis in MCF‐7 cells and regression of mammary tumor in SD rats

**S. Dixit^1^, J. G. Meher^2^, M. K. Chourasia^2^ & R. Konwar^1^**

*^1^Division of Endocrinology, CSIR‐Central Drug Research Institute, Lucknow, India; ^2^Pharmaceutics, Pharmacokinetics and Metabolism Division, CSIR‐Central Drug Research Institute, Lucknow, Uttar Pradesh, India*

*Email:* shivani.dixit.cdri@gmail.com

Vitamin D (VitD3) is effective against various cancers but hyperglycaemia at higher dose is an obstruction for its usage. A combination therapy with phytochemicals is hypothesized to be fruitful in using VitD3 in cancer treatment. Present study is directed towards exploration of anticancer activity of VitD3 in combination with gallic acid against breast cancer. Cytotoxicity of VitD3 and GA were done by MTT assay. *In vitro* anticancer evaluations *viz*. cellular morphology, DNA fragmentation, cell cycle distribution, ROS, MMP alteration, apoptosis and Western blotting were performed. *In vivo* anti‐tumor activity was performed in syngenic mammary tumor model in SD rats. VitD3+GA treatment caused 1.7 fold increase in percent cell inhibition in MCF‐7 cells than VitD3 and GA alone. Marked cellular changes and DNA fragmentation were observed in combination treated groups. G2/M phase arrest, increased‐apoptotic population, increased‐ROS level and disrupted MMP were also seen. Increased expression of Bax, p21, cytochrome c, cleaved caspase‐9, cleaved caspase‐8, cleaved caspase‐7 and decrease in Bcl2 level was observed and apoptosis in MCF‐7 cells was through PI3K/Akt pathway. VitD3+GA caused regression of mammary tumor in SD rats and did not increase serum calcium level significantly. VitD3+GA groups showed less number of metastatic lung nodules and apoptotic changes in tumor sections. Cytotoxicity and other *in vitro* anticancer evaluation suggested that the combination of VitD3+GA could bring apoptosis in MCF‐7 cells in a greater extent than individual components. The improved therapy is attributed to the combinatory action and sensitization of MCF‐7 cells leading to enhanced anticancer action. Further it was found that anticancer and pro‐apoptotic activities in MCF‐7 cells, are mediated through upstream IGF‐IR*β*/PI3K/Akt signalling pathway followed by caspase mediated intrinsic and extrinsic pathway of apoptotic cell death. In the *in vivo* model, VitD3+GA caused regression of syngenic mammary tumor in SD rats and did not increase serum calcium significantly. VitD3 and GA combination exhibited potent anti‐breast cancer activity in comparison to VitD3 alone; hence this combination can be validated with more specific studies and employed for further clinical investigation.

## Potential of *Moringin,* an isothiocyanate against triple negative breast cancer

**S. Mishra, S. S. Verma, V. Rai, N. Awasthee & S. C. Gupta**

*Laboratory for Translational Cancer Research, Department of Biochemistry, Institute of Science, Banaras Hindu University, Varanasi, Uttar Pradesh, India*

*Email:* shruti25mishra87@gmail.com

Although triple negative breast cancer (TNBC) likes other cancer types, is a multigenic disease, most of the currently available drugs are based on the modulation of more specifically a single target. Therefore, these drugs are less likely to be effective. The main focus of this study was to examine the potential of moringin against TNBC. We delineated the underlying mechanism by which moringin exhibits anti‐cancer activities against TNBC. We used triple negative MDAMB‐231 cell line during the study. Moringin containing benzyl‐isothiocyanate, is produced by myrosinase‐catalyzed hydrolysis of glucomoringin (GMG). The MTT and clonogenic assays were used for the cytotoxicity; DAPI staining for apoptosis and wound healing assay for cell migration. Moringin inhibited the viability and proliferation of MDAMB‐231 cells in a way that is proportional to the dose as well as time. When given a minimal concentration of 25 *μ*mol/L, moringin inhibited the proliferation of cancer cells after 24 h of treatment. The IC50 of moringin against TNBC was observed in the range of 21 *μ*mol/L to 4.5 *μ*mol/L after treatment for 24 and 48 h, respectively. The long‐term colony formation by cancer cells that mimics *in vivo* situation, was also significantly inhibited by moringin. DAPI staining suggested that the moringin induced apoptosis in breast cancer cells. Moringin also induced ROS generation in MDAMB‐231 cells. As examined by wound healing assay, moringin inhibited the migration of cells. Overall, these observations suggest that moringin exhibit anti‐cancer activities against triple‐negative breast cancer cells. The generation of ROS may contribute to its anti‐cancer activities. Studies are in progress to substantiate these observations using multiple TNBC cells lines and to elucidate the in‐depth molecular mechanism.

## Competitive binding of peptides out of Ras binding domain RBD downregulate mTORC2 activation

**S. A. Malik, J. Miyan, M. Asif, R. Srivastava, P. Dubey & S. Bhaduria**

*Division of Toxicology, CSIR‐Central Drug Research Institute, Lucknow, Uttar Pradesh, India*

*Email:* malik.showkat1988@gmail.com

Identifying and deciphering the dynamics of signalling pathways that are uniquely operational in cancer cells is of prime significance because this could enable selective targeting of cancer cells with minimal adverse effect on normal cells. Such pathways can be effective targets for devising anti‐cancer strategies of improved efficacy. The mammalian target of rapamycin complex2 (mTORC2) is a promising target candidate for such an inhibitor. The mTORC2 is a multiprotein complex comprised of Rictor (rapamycin insensitive companion of mTOR) mSIN, mLST8, Deptor and Protor. Recent studies shows that mTORC2 activity is essential for cancer cell vitality and transformation, but not much required in normal cells; due to which the interest in developing specific mTORC2 targeted inhibitor is intensified. The main aim of this study was to devise selective mTORC2 inhibitory strategy and evaluation of its anticancer efficacy. mTORC2 expression in control and peptide treated cells where analysed by western blotting and chemiluminescence. Effect of peptides on mTORC2 regulated actin remodelling and cell invasion was done by confocal microscopy and cell invasion respectively. Pertinent to localization of active RAS at plasma membrane and also reports about interaction of this active Ras with mSIN an integral component of mTORC2 we evaluated the interaction of mSIN Ras as a therapeutic target to inhibit mTORC2. mSIN an integral part of mTORC2 interacts with active GTP bound form of Ras through its Ras binding domain (RBD) and inhibits its signalling. This prompted us to synthesize various peptide sequences that might compete for interaction with Ras. Results that we get showed all the peptide fraction were able to inhibit mTORC2 to a varied degree and no peptide had any inhibitory effect on the mTORC1 activation. mTORC2 is a known regulator of cytoskeleton, it regulates the actin remodelling. The p4 and p4.1 treated cells prevented actin polymerization with a more prominent inhibition in cells treated particularly with p4.1. Insulin and serum mediated activation of mTORC2 led to higher invasive potential in MDA‐MB 231 and DU‐145 Cells which was diminished to a significant level in cells pretreated p4 and p4.1 peptides. Our preliminary studies suggest that peptides created out of RBD domain of mSIN prevent ras mSIN interaction which inturn causes the mTORC2 downregulation. The results of our study will have important clinical implications for anticancer therapy as there is growing appreciation of mTORC2's vital function in tumorigenesis. This can act as a rational selection for combination therapies and will enhance the success of mTOR directed drug therapies.

## In‐vivo toxicity and efficacy study of SM‐01’ Hydrogel for the management of dermal irradiation induced wounds

**S. Kulshrestha, S. Singh, R. Chawla & M. Basu**

*Division of CBRN Defence, Institute of Nuclear Medicine and Allied Sciences, DRDO New Delhi, India*

*Email:* kulsshweta13@gmail.com

In radiotherapy cases, radiation toxicity remains a significant clinical issue that influences treatment outcome, patient quality of life and survivorship. Exposure to a large external dose of radiation can cause local or loco‐regional radiological topical or cutaneous injury and combine damage to surrounding tissue. Conventional treatments for skin desquamation or skin breakdown are ineffective and complicated. Till date, there is no well known therapeutic modality is available that can overcome the topical radiation effect. Introduction of new therapeutics and expanded knowledge considering treatment of dermal radiation wounds are necessary. Therefore, we herein proposed the toxicity and efficacy of SM‐01 hydrogel, as a new emerging therapeutic that could be used to treat dermal radiation induced wounds. It sustains the cGMP‐enhancing effect of nitric oxide (NO), thereby regulating L‐arginine‐NO‐cGMP pathway in dermal tissues. To study the *in‐vivo* dermal, cyto‐dermal toxicity and efficacy of SM‐01 for the management of dermal radiation induced wound. Repeated dose dermal toxicity of SM‐01 was performed according to OECD guideline 410 and cellular toxicity has been carried out using a technique flow cytometry. Further, radiation induced wound was optimized and developed on Sprague Dawley rats flanked skin with a single large dose of ionization radiation. Acute cutaneous damage & wound healing efficacy of SM‐01 was evaluated by clinical skin grading, histology and area contraction followed by anti‐oxidant assays. SM‐01 hydrogel was found to be non‐toxic at dermal and cellular level. In‐vivo efficacy of SM‐01 treatment reduced the size of irradiation induced wound by 80%–85% compared to untreated. Other healing efficacy parameters such as body weight, skin damage, tensile strength was also found to be higher in SM‐01 treated wounds. Further, histopathology validated higher collagen crosslinks and skin integrity in SM‐01 groups. SM‐01 hydrogel were found to be safe and helps in reducing acute radiation damage in rats and could be used as a better healing agent.

## Prenatal exposure to arsenic induces epidermal hyperplasia leading to local inflammation in skin and an aggravated response in a two stage model of cutaneous carcinogenesis in mice

**S. Gangopadhyay^1,2^, S. Shukla^1,3^, R. D. Singh^1,2^, H. Khan^1,2^, R. Tiwari^1^ & V. Srivastava^1^**

*^1^Systems Toxicology and Risk Assessment Group, CSIR‐Indian Institute of Toxicology Research, Lucknow, Uttar Pradesh, India; ^2^Academy of Scientific and Industrial Research (AcSIR), CSIR‐Indian Institute of Toxicology Research, Lucknow, Uttar Pradesh, India; ^3^Dr. Shakuntala Misra National Rehabilitation University, Department of Microbiology, Lucknow, Uttar Pradesh, India*

*Email:* sid9843@gmail.com

Chronic low level exposure to arsenic in drinking water likely puts the subjects at increased risk for tumorigenesis. Most studies in this field are confined to adult exposure to arsenic at high doses. The following study shows that only gestational or prenatal exposure to arsenic at environmentally relevant doses is sufficient to induce an aggravated response towards a two stage chemical carcinogenesis model in mice. Using 7,12dimethylbenz[a]anthracene as a tumour initiator and 12‐O‐tetradecanoyl phorbol‐13‐acetate (TPA) as promoter we analysed the progression of tumours in prenatally arsenic exposed swiss albino mice. The percentage of tumour bearing mice were 23%, 68% and 63% respectively in control, 0.4 ppm and 4 ppm prenatal exposure groups at termination. The histological features showed an aggravated response in the treatment groups with pathological signs of malignant squamous cell carcinoma compared to control. The mice that did not receive any initiator/promoter treatment showed no gross macroscopic features after 1 year but microscopically the skin showed a marked thickening of the epithelial layer and hyperkeratosis that developed without any further treatment of arsenic. By the tested protocols and doses, our data suggests that, only prenatal exposure to low levels of arsenic in drinking water is sufficient to compromise skin quality and with additional exposure to environmental carcinogens may cause cancer at an early age of onset.

## Assessment of the cytotoxicity of diethylene glycol monoethyl ether on a neuroblastoma cell line via *in vitro* assays

**S. Srivastava, S. Mishra, J. Dewangan, A. Divakar, P. K. Pandey & S. K. Rath**

*PCS 103 Genotoxicity Lab, Division of Toxicology and Experimental Medicine, CSIR‐Central Drug Research Institute, Lucknow, Uttar Pradesh, India*

*Email:* sonal.srivastava24@gmail.com

Diethylene Glycol Monoethyl Ether (DEGEE) is an industrial solvent and a drug excipient used in formulation of drugs and for administration of drug through IP or oral routes. Experimental screening of the toxicity of these type of chemical compounds is crucial to assure its safety and effectiveness. In an isolated case report, an alcoholic male (aged 44) drank approximately 300 mL of a liquid containing 47% DEGEE (about 2000 mg/kg). Severe symptoms of central nervous system, dyspnoea, thirst and acidosis occurred. The subject recovered following symptomatic treatment. The Neuro2A cells were used to evaluate the toxic potential of DEGEE and the mechanism of cell death induction. In the present study, the cytotoxicity of DEGEE against Neuro2A neuroblastoma cell line was evaluated via Sulforhodamine B (SRB) assay in dose as well as time dependent manner. The effect on cellular DNA fragmentation, generation of reactive oxygen species (ROS) and alteration in mitochondrial membrane potential (MMP) was studied via fluorescence microscopy observation using Hoechst 33258, H2DCFDA and JC‐1 stains, respectively. The effect on cell apoptosis induced by DEGEE was investigated via flow cytometry analysis. The results showed that the cytotoxic effect of DEGEE on Neuro 2A cells was dependent on the concentration and length of exposure. Nucleus of untreated cells were intact, round in shape and stained dimly blue, whereas brightly stained punctuated nuclei were observed upon DEGEE exposure. Dose dependent increase in intracellular ROS levels were observed 12 h post treatment. Untreated cells were found with high JC‐1 aggregates which emits red colour. However, cells exposed to DEGEE expressed significant more JC‐1 monomeric form which emits green fluorescence, confirming alteration of the mitochondrial membrane potential. An increase the fraction of cells undergoing apoptosis was observed in a dose dependent manner. Intracellular ROS accumulation causes disruption in MMP which regulates inflammation and programmed cell death pathways. The result of this study suggests that DEGEE has toxic potential when tested *in vitro*. As it has clinical application as an excipient, evaluation of its toxic potential on *in vivo* models becomes an important aspect.

## PDE5 inhibitors as cancer chemo preventive agents in alcohol‐aflatoxin B1 induced hepatocellular carcinoma

**S. K. Chhonker, D. Rawat & R. K. Koiri**

*Biochemistry Lab, Department of Zoology, Dr. Harisingh Gour Central University, Sagar, Madhya Pradesh, India*

*Email:* saurabhchandra516@gmail.com

Hepatocellular carcinoma (HCC) is the fifth common malignancy worldwide and the third leading cause of cancer related death. Frequency of HCC occurrence has increased globally, particularly in China, Asia, Africa and curative approaches for HCC are very confined. HCC is a chemo‐resistant tumor and conventional cytotoxic chemotherapy has not provided clinical benefit or prolonged survival for patients with advanced HCC. The present study aims to investigate the therapeutic efficacy of phosphodiesterase‐5 inhibitor against alcohol‐aflatoxin B1 induced HCC. Rats were randomly divided in five groups’ i.e. normal control, alcohol control, HCC control and HCC experimental (HCC+Tad, HCC+Sil). Except normal control group, rats of all other groups were fed with 5% alcohol via drinking water for 3 weeks. After 3 weeks alcohol treatment, two successive doses of AFB1 (1 mg/kg/bw) was administered to induce HCC in rats. HCC rats were treated with PDE5 inhibitor (Tadalafil & Sildenafil) via drinking water for two weeks. During HCC progression, significant increased expression of placental form of GST & alpha‐fetoprotein was observed with a concomitant alternation in liver morphology and histology. Further enhanced production of lactate and increased expression of Glut1, LDH, PFKFB3, Akt and c‐Myc & decreased expression of genes of antioxidant pathway (SOD, Catalase, GPx and GR) with a concomitant alteration in the level of lipid peroxidation was observed. Post treatment with phosphodiesterase 5 inhibitor (tadalafil & sildenafil) significantly restored the above parameters towards normal via modulation of cGMP regulated pathways. Results suggests that PDE5 inhibitors may prevent the development and progression of HCC by increasing the level of cGMP and modulating the key parameters of glycolytic‐antioxidant pathway.

## Buccal cell changes in oral submucous fibrosis

**S. Jaiswal, S. Jahan, S. Nigam, R. K. Srivastava & P. K. Sharma**

*Department of Anatomy, Era Medical University, Lucknow, Uttar Pradesh, India*

*Email:* jaiswalsonia2008@gmail.com

Oral submucous fibrosis (OSMF) is a condition that was first described in the 1950s. It is caused as a result of addiction to harmful areca nut products with or without tobacco. The rationale of using exfoliative cytology or liquid based cytology (using saliva as a biofluid) in our study lies in the epithelial physiology where continuous exfoliation of epithelial cells is a part of physiological turnover. Deeper cells, which are strongly adhered in normal conditions, become loose in the case of malignancy and exfoliate along with superficial cells. Our aim in this study was to compare the cellular changes such as formation of micronuclei within the cell and cytomorphometric analysis of the buccal mucosal cells of OSMF patients with that of normal controls. We identified 33 such cases of OSMF on the basis of oral inspection and examination. We used exfoliative cytology and liquid based cytology to obtain buccal cells. The smear thus prepared was stained with feulgen fast green, acridine orange and papanicolaou. Micronuclei were identified and cytomorphometric analysis was done using Adelta software. There was a change in the hue of Papanicolaou from pink to purplish indicating the degree of keratinization from normal cells to cells affected by OSMF. Acridine orange gave a green emission at wavelength 480–490 to normal cells, while it gave a bright red fluorescence in cells undergoing apoptosis. Mean cellular diameter decreased from normal‐cells affected oral lesions. Mean nuclear cytoplasmic ratio increased from normal‐cells to those affected by oral lesions. Frequency of micronuclei increased from normal to the cells affected by oral lesions. Buccal cell mutations in premalignant and malignant lesions can serve as a useful tool for the bio‐monitoring of oral lesions. Exfoliative Cytology being minimally invasive and cost effective can help in mass screening programmes.

## MAPKAPK2 regulates the progression of head and neck squamous cell carcinoma by stabilizing TNF‐*α*, VEGF and destabilizing p27, MKP‐1 transcripts

**S. Soni^1,2^, P. Anand^1,2^ & Y. S. Padwad^1,2^**

*^1^Pharmacology and Toxicology Lab, Food and Nutraceuticals Division, CSIR‐Institute of Himalayan Bioresource Technology (CSIR‐IHBT), Palampur, Himachal Pradesh, India; ^2^Academy of Scientific and Innovative Research, Chennai, Tamil Nadu, India*

*Email:* sourabhsoniplp@gmail.com

The p38 mitogen‐activated protein kinase (MAPK) pathway has been implicated in pathological conditions like inflammation and metastasis. Post‐transcriptional regulation of genes harboring adenine/uridine‐rich elements in their 3′‐untranslated region (3′‐UTR) is controlled by MAPK‐activated protein kinase 2 (MAPKAPK2 or MK2), a downstream substrate of the p38/MAPK. In response to diverse extracellular stimuli, MK2 influences crucial signaling events, regulates inflammatory cytokines, mRNA stability and cellular processes including cell‐cycle regulation, angiogenesis, proliferation, metastasis and cell death. Till date, the biological significance of MK2 pathway in cancer is not well understood. In this study, we have tried to elucidate the mechanistic role of MK2 in head and neck squamous cell carcinoma (HNSCC) progression. Human clinical malignant and adjacent normal tissue samples were processed for histopathological, immunohistochemical, western blotting and quantitative real time‐polymerase chain reaction analysis. The findings were validated *in vitro* in HNSCC cell lines. Transcript levels of susceptible genes harboring RNA‐binding sites in their 3′‐UTRs were evaluated along with their post‐transcriptional stability in MK2‐knockdown (MK2KD) cells in both normoxic and hypoxic conditions. We have reported reproducible MK2 overexpression in HNSCC and its crucial role in HNSCC pathogenesis by altering the translational landscape. Increased stability of cyclin‐dependent kinase inhibitor 1B (p27) and mitogen‐activated protein kinase phosphatase‐1 (MKP‐1) transcripts and decreased half‐life of tumor necrosis factor‐alpha (TNF‐*α*) and vascular endothelial growth factor (VEGF) transcripts in MK2KD cells suggested that MK2 regulates their mRNA turnover. Post‐transcriptional regulation of gene expression in tumor versus normal tissues is a highly unexplored area. By better understanding the role that MK2 plays in tumor progression could provide novel insights into the unsolved puzzles of the post‐transcriptional gene regulation in cancer and metastasis. This will eventually pave the way for novel therapies and better tailoring of present treatment modalities for the patients. Taken together, our results portray a critical role of MK2 in modulating HNSCC pathogenesis and implicate MK2 as a prominent tumor marker. Majority of p38 inhibitors have already failed in the clinical trials, thus, we have tried to unveil MK2 as a potential novel anticancer therapeutic target in the management of HNSCC.

## Survival of gliomas through protease activated receptor (par) mediated pathway

**S. Tripathy & A. Prakash**

*Department of Biotechnology, Babasaheb Bhimrao Ambedkar University, Lucknow, Uttar Pradesh, India*

*Email:* sukanya3bcs@gmail.com

Malignant brain tumors account for almost 2 percent of all cancers. The most common malignant brain tumor is glioblastoma multiforme, and patients with this type of tumor have poor prognoses. The invasiveness prevents effective surgical resection or chemotherapy. In order to overcome the barriers of treatment options, a continual search is going on in the field of cancer neurobiology. In similar stream it was found that protease activated receptors (PARs), a family of G‐protein coupled receptors play a polar role in blood coagulation and fibrinolysis. In many cancers like breast cancer, PAR1 fails to down‐regulate leading to persistent activation of ERK1/2 that is involved in the cellular invasion. Similarly in gliomas activation of PARs leads to altered ion channel which further affects the apoptosis and other survival pathway molecules. These receptors are found to be activated by specific peptides like TFLLR and thrombin which further causes cellular growth and invasion. The various inhibitor based study like argatroban, a thrombin antagonist causes attenuation of tumor mass development and hence prolonging the survival time of organism. Specifically, PAR‐1 deletion in the glioma leads to the reduced HIF‐1*α* and VEGF expression levels. Thus, from above studies, we may predict that the study of PAR and its related molecule may prove as one of the potential therapeutic targets for glioma growth regulation.

## Isodeoxyelephantopin, a novel sesquiterpene lactone, induces apoptosis, inhibits invasion, and abolishes cancer cell proliferation through suppression of nuclear factor‐ĸB (NF‐ĸB) activation

**S. S. Verma^1^, N. Awasthee^1^, V. Rai^1^, S. Mishra^1^, M. S. Nair^2^ & S. C. Gupta^1^**

*^1^Laboratory for Translational Cancer Research, Department of Biochemistry, Institute of Science, Banaras Hindu University, Varanasi, Uttar Pradesh, India; ^2^Division of Organic Chemistry, CSIR‐National Institute for Interdisciplinary Science and Technology, Thiruvananthapuram, Kerala, India*

*Email:* sumit.mhg.bhu14@gmail.com

Although therapeutic plants have been used since ancient time, their active ingredient and underlying mechanism remains poorly understood. *Elephantopus scaber* Linn. is one such medicinal plant that has been used for the treatment of chronic diseases. Several agents including Isodeoxyelephantopin (IDET) and deoxyelephantopin (DET) have been isolated from this plant. The aim of this study was to examine if IDET and DET possess anti‐tumorigenic activities. If so, whether these agents can modulate pro‐inflammatory NF‐*κ*B activation in cancer cells? We used different breast cancer cells (MCF‐7, MDA‐MB‐231, MDA‐MB‐468, MDA‐MB‐453) and C6‐glioma cell lines. The proliferation and viability of cancer cells was examined by MTT assay. We also examined if IDET can sensitize breast cancer cells to doxorubicin. The ROS inducing potential of IDET was examined by DCF‐DA staining. The other parameters used were AO/PI staining, DAPI staining and subG1 analysis for apoptosis, and western blotting for cell survival proteins. IDET was found to suppress the viability and proliferation of different breast cancer cells and glioma cell lines in a dose‐ and time‐dependent manner. IDET was more effective as compared to DET. The sesquiterpene promoted apoptosis in cancer cells. The sesquiterpene also suppressed expression of cell survival proteins in tumor cells. Besides, IDET was found to sensitize cancer cells to doxorubicin. Furthermore, the migration of MDA‐MB‐231 was suppressed by IDET. Although okadaic acid induced NF‐kB activation in breast cancer cells, the same was suppressed by the use of IDET. The sesquiterpene also induced ROS generation in breast cancer cells. Overall, these observations suggest that IDET is more potent as compared to DET against breast and glioma cells. Further, the inhibitory impacts of IDET on NF‐*κ*B activation may contribute to its anti‐cancer potential. Studies are in progress to examine if ROS is involved in the suppression of NF‐*κ*B activation induced by IDET.

## Discovery of the first DNA ligase‐I inhibitor with *in‐vivo* breast cancer activity

**D. K. Singh, M. Kamil Hussain, S. Kumar, S. Krishna, M. I. Siddiqi, D. Datta, K. Hajela & D. Banerjee**

*MSB Division CSIR‐Central Drug Research Institute, Lucknow, Uttar Pradesh, India*

*Email:* ksushil343@gmail.com

Worldwide breast cancer is the leading cause of cancer in women, even surpassing cervical cancer in the recent past. Due to high level of drug resistance in cancer cells, treatment of breast cancer is the major concern, and results in poor prognosis and recurrence of breast cancer. Thus, this area needs the screening, identification and development of novel therapeutic targets and molecules. In this study, a novel class of Benzocoumarin‐Stilbene hybrid molecules were synthesized and evaluated for their antiproliferative activity against various cancer cell lines followed by *in vivo* antitumor activity in a mouse model of cancer. The most promising molecule among the series, i.e. compound (E)‐4‐(3,5‐dimethoxystyryl)‐2H‐benzo[*h*]chromen‐2‐one (compound 19) showed excellent antiproliferative activity in breast cancer cell lines (MDA‐MB‐231 and 4T1) and decreased the tumor size in the *in‐vivo* 4T1 cell‐induced orthotopic syngeneic mouse breast cancer model. The mechanistic studies of compound 19 by various biochemical, cell biology and biophysical experiments suggest that the compound binds to and inhibits the human DNA ligase I enzyme activity. The inhibition of this activity leads to the accumulation of DNA strand breaks that might be the cause for significant reduction in tumor growth and may constitute a promising next‐generation therapy against breast cancers.

## Renal cell carcinoma a case report

**T. Haider**

*Department of Anatomy, Era Medical University, Lucknow, Uttar Pradesh, India*

*Email:* drtahsinh@gmail.com

In the current era, because of prevalence of sonography, renal cell carcinoma usually can be detected in early stages and a huge tumor is rarely encountered RCC account for approximately 80%–85% of primary renal tumor and are most common form of malignant renal tumor. Renal cell carcinoma was first reported by Paul Grawtz in 1883. It was first named after him as Grawitz tumor or hypernephroma according to his belief that the tumor originated in adrenal rests at the upper pole of kidney. Later the origin of this tumor in renal tubular cells was documented. We report a case of 60 years old male with history of two episodes of hematuria O However,no flank pain, abdomen pain, nausea, vomiting or bowel habit changes were reported by the patient. Examination per abdomen there was no palpable mass. Ultrasonography revealed a renal mass. On CT abdomen plain and contrast a well defined large lobulated mass was seen. Patient got operated.

## Metabolic and immunophenotypic characterization of oral squamous cell carcinoma to ascertain tumor aggressiveness

**T. S. Saluja & S. K. Singh**

*Stem Cell/Cell Culture Lab., Centre for Advance Research, King George Medical University, Lucknow, Uttar Pradesh, India*

*Email:* salujatajindersingh@gmail.com

Cancer is one of the principal causes of mortality in developing and developed nations. Globally, in the year 2012, approximately 3 lakh new cases and 1.5 lakh deaths were ascribed to oral cancer. An increased morbidity and mortality associated with oral squamous cell carcinoma (OSCC) is not only due to its delayed diagnosis but also to the inadequate response of tumor to treatment. Tumor initiating cells known as cancer stem cells (CSC) drive tumor growth resulting in heterogeneous subpopulations within the tumor. The lack of knowledge in oral CSCs and heterogeneity in OSCC makes our understanding very limited about the inherent nature of this malignancy. The present study aims at evaluating metabolic phenotype and isolation of cancer stem cells using putative cancer stem cell markers in different grades of oral squamous cell carcinoma. The metabolic phenotype was ascertained immunohistochemically using LDH5 antibody. Different cancer stem cell populations were analyzed by flow cytometry using putative surface cancer stem cell markers (CD24, CD44 etc.).Metabolic phenotype varied between different grades of OSCC. The primary culture of OSCC cells was best achieved using explant and multi‐step enzymatic digestion method. Primary OSCC cells expressed combination of stem cell markers that did not change in series of cell passages. The inhomogeneity and varied extent of tissue oxygenation between tumours with similar histopathological grading underlines the fact that tumor behaviour cannot be ascertained alone on histological basis. Evaluation of metabolic phenotype could help in segregating aggressive tumors from non‐aggressive ones. The multi‐marker panel refines our search for CSCs. Immunophenotypic characterization of oral cancer showing tumor cells expressing combination of CSC markers may be more aggressive.

## Benzimidazole based self‐assembled organic nanoparticles exhibit enhanced cytotoxic potential towards proliferating human breast cancer cells

**V. Dhanwal^1,2^, A. Singh^3^, D. Nayak^2^, G. Kaur^1^, N. Singh^3^, A. Goswami^2^ & N. Kaur^4^**

*^1^Centre for Nanoscience & Nanotechnology, Panjab University, Chandigarh, India; ^2^Cancer Pharmacology Division, CSIR‐Indian Institute of Integrative Medicine, Jammu, India; ^3^Department of Chemistry, Indian Institute Technology, Ropar (Punjab), India^4^Department of Chemistry, Panjab University, Chandigarh, India*

*Email:* Vandna.dhanwal22@gmail.com

Benzimidazole derivatives (heterocyclic organic compound with fusion of benzene and imidazole ring) have gained significant importance in medicinal chemistry due to their remarkable cytotoxic/anticancer activities. On the other hand, organic nanoparticles (ONPs) have gain significant research interests because of certain advantages like ease of preparation, biodegradability, superior stability and solubility than organic compounds in biological fluids. The aim of this study was to prepare ONPs of some benzimidazole derivatives and comparative cytotoxic/anticancer activity studies of these compounds with their respective ONPs. Another goal of the study was to investigate the molecular mechanism of action of apoptosis induced by the most potent derivative and its ONPs in *in vitro* cancer models. ONPs of synthesized benzimidazole compounds were formulated by re‐precipitation method. Cell viability assays were carried out to determine the cytotoxicity of the compounds and their ONPs. Apoptosis assay and colony formation assay were performed to examine the effect of the most active compound and its ONPs on apoptosis induction and cell proliferation. ROS determination assay, caspase 3/7 activity assay, mitochondrial membrane potential studies and western blot analyses were to unveil the molecular mechanism of apoptosis induction by the ONPs. Preparation, characterization, and investigation of cytotoxic activities of 1,2‐disubstituted benzimidazole derivatives (ASH1 – ASH15) and their respective ONPs in various cancer and normal cell lines were performed successfully. The most potent derivative (ASH6) displayed significant increase in cytotoxic potential (˜5 fold) in MCF7 breast cancer cells in the form of organic nanoparticles (ASH6 ONPs). The ONPs induced ROS activity leading to mitochondrial damage, loss of cell proliferation, increase in caspase activity and induction of apoptosis in proliferating MCF7 cells. Western blot experiments unveiled that ASH6 ONPs downregulate the expressions of oncogenic proteins such as AKT, NF‐kB, Vimentin and Survivin, whereas upregulate the expression of tumor suppressor Par‐4 in a concentration dependent manner much more effectively compared to the control and ASH6 alone. Our study reveals the ease in preparation of heterocyclic ONPs with enhanced cytotoxic/apoptosis inducing potential, its selectivity towards mammalian breast cancer cells and less toxicity proclaim these nanoparticles as promising anti‐cancer agents.

## Inhibition of dimethylnitrosamine induced hepatic cancer by zinc oxide nanoparticles in laboratory rat

**V. Rani, Y. Verma & S. V. S. Rana**

*Toxicology laboratory, Department of Zoology, Ch. Charan Singh University Meerut, Uttar Pradesh, India*

*Email:* sharmavarsha1091@gmail.com

Nanoparticles with their selective target capabilities and efficacy are becoming increasingly important in modern cancer therapy and starting to overshadow traditional cancer therapies. Zinc oxide nanoparticles with their unique properties such as biocompatibility, high selectivity, enhanced cytotoxicity may be promising anticancer agent against various cancers including liver cancer. Dimethylnitrosamine is a potent hepatotoxin which exerts carcinogenic effects through metabolic activation mechanisms in liver of experimental animals. Various drugs/nutrients have been examined for their protective effects against dimethylnitrosamine induced hepatotoxicity/hepatocarcinogenicity. However anticancer/protective nature of zinc oxide nanoparticles against dimethylnitrosamine induced hepatic cancer is not known. Therefore an attempt was made to assess the protective effects of zinc oxide nanoparticles, if any, against dimethylnitrosamine induced carcinogenicity. The aim of present study was to examine the ameliorating effects of zinc oxide nanoparticles against dimethylnitrosamine induced hepatic carcinogenesis in laboratory rat as an *in vivo* model. Experimental animals (rats of Wistar strain) were divided into four groups each containing five rats. Rats of one of four groups were injected dimethylnitrosamine (2 *μ*L/100 g b.w. ip) for fifteen days and rats of other group treated with dimethylnitrosamine subsequently and administered dose of zinc oxide nanoparticles (50 mg/kg) for thirty days. Rats of other group were also treated by only with zinc oxide nanoparticles. Rats of group four were injected saline only and treated as control. After scheduled treatments blood samples were obtained through cardiac puncture and processed for the estimation of ALT, AST, ALP, LDH, bilirubin, TNF‐*α*, IL‐12 and VEGF. Liver were also carefully removed and processed for MDA, NO, H2O2 and for histological studies. The size and shape of zinc oxide nanoparticles were confirmed by SEM, TEM, EDAX, X‐RD and by estimating the zeta potential. Results of present study showed that a post treatment of zinc oxide nanoparticles to dimethylnitrosamine exposed rats reduces ALT, AST, LDH, LPO, fibrosis showing protective function of zinc oxide nanoparticles. Decreased values of inflammatory cytokines such as TNF‐*α*, IL‐12 and growth factor (VEGF) also support the inhibitory function of zinc oxide nanoparticles. Histopathological observations also confirmed the protective role of zinc oxide nanoparticles. Post treatment of zinc oxide nanoparticles to dimethylnitrosamine treated rats improved liver functions. It also inhibited the oxidative stress, diminishes liver cell necrosis and cytokines level. Furthermore, depressed level of serum vascular endothelial growth factor shows that zinc oxide nanoparticles display protective effects against dimethylnitrosamine induced hepatotoxicity/carcinogenicity.

## Boosting combination chemotherapeutic efficacy of Metformin and Topotecan using ion trapping assisted ratiometric delivery via pseudo cell like mesoporous silica nanoparticles

**V. T. Banala, S. Urandur, R. Shukla, G. Pandey, N. Mittaplley, M. Dwivedi & P. R. Mishra**

*Pharmaceutics Division, CSIR‐Central Drug Research Institute Lucknow, Uttar Pradesh, India*

*Email:* venkateshteja56@gmail.com

Although metformin possess reasonable anticancer activity, its therapeutic application is limited due to high water solubility, rapid elimination and sub‐IC50 tumor concentrations. The present study aims in addressing the translational challenges of metformin chemotherapy by high drug pay load intracellular controlled co‐delivery of synergistic metformin (MET) and topotecan (TPT). This was accomplished by the development of novel in‐situ hydrophobic ion pairing(HIP) driven active loading approach using lipid bilayer coated Mesoporous Silica Nanoparticles (MSNs) as templates. HIP between Metformin/Pamoic acid and Topotecan/Pamoic acid was initially evaluated using H^1^ NMR. Further Mesoporous silica nanoparticles were prepared by Stober's process. The active loading procedure was developed and optimized using pamoic acid loaded lipid bilayer coated MSNs as templates for the first time. Process parameters that effect the drug loading was evaluated separately for both drugs, in situ HIP formation inside the MSNs were determined using IR. The developed delivery system was evaluated for the size, zeta potential using DLS technique (Malvern Zeta sizer) and TEM analysis. Drug loading and in vitro drug release was evaluated using HPLC. *In vitro* cytotoxicity, cell cycle arrest, apoptosis and cell uptake were evaluated. H^1^‐NMR confirms the HIP formation between Metformin/Pamoic acid and Topotecan/Pamoic acid with enhanced hydrophobicity of ˜ 40‐fold and 7.7‐fold respectively. The mean particle size of MSNs before and after drug loading was found to be 97.33 ± 6.89 nm and 110 ± 9.65 nm, respectively. *In situ* HIP was confirmed by their characteristic FTIR peaks. The maximum drug loading for metformin (40% w/w) and topotecan (29% w/w) was accomplished in the presence of gradient ion triethylamine and the external phase with pH 9.0 at 65°C. In vitro drug release was controlled up to 48 h with <85% and <65% in case of metformin and topotecan MSNs. Synergy score was increased for drug loaded MSNs compared to plain drugs in cytotoxicity studies. Similar trend was found in cell cycle arrest and apoptosis studies. The drawbacks associated with metformin and topotecan were successfully addressed using *in situ* hydrophobic ion pairing approach with high drug payload and controlled release profile.

## Erastin mediated non‐apoptotic cell death in head and neck cancer stem cells

**V. K. Srivastava^1^, N. Rastogi^2^, I. J. Saifi^1^, P. Sexana^1^, M. Kaleem Ahmed^3^, D. P. Mishra^2^, M. L. B. Bhatt^4^ & K. M. Ansari^1^**

*^1^Food Toxicology Division, CSIR‐Indian Institute of Toxicology Research, Lucknow, India; ^2^Division of Endocrinology, CSIR‐Central Drug Research Institute, Lucknow, India; ^3^Department of Biochemistry, King George's Medical University, Lucknow, India; ^4^Department of Radiotherapy, King George's Medical University, Lucknow, India*

*Email:* vikascdri@gmail.com

Tumour growth is highly dependent upon the metabolic rearrangement and stemness. Recently, it has been shown that some cancer cells exhibits cancer stem cells (CSCs)like property. These CSCs consist two biologically distinct CSC phenotypes both of which have high levels of expression of CD44 but differ in their levels of expression of epithelial specific antigen (ESA) One phenotype has a CD44^high^/ESA^high^ marker pattern, is proliferative, forms cohesive colonies in vitro, and has epithelial characteristics and the other has a CD44^high^/ESA^low^ marker pattern, is migratory, and has mesenchymal characteristics. In the present study we used squamous cell carcinoma of head and neck (SCCHN) cells lines (Cal‐27 and FaDu) and patients derived primary cells for flow cytometry based cancer stem cell sorting to identify biologically distinct CSC phenotypes and also inhibits altered energy metabolism pathways key components with specific inhibitors along with conventional therapeutic strategy to overcome therapy resistance and recurrence of disease. The cytotoxicity of Cisplatin alone in CSCs was low, however in the combination of Erastin (potent ferroptosis inducer) and 2DG (glycolysis inhibitor) increased cytotoxicity *in vitro*. Most importantly, combined treatment intensified inhibition of clonogenicity and spheroid formation indicating that CSCs characteristics has targeted. In *ex‐vivo* validation experimentation, the combination treatment inhibits the cell proliferation and induces cell death significantly. No pronounced cytotoxicity was observed in normal cells *in vitro*. On the basis of present study, we can conclude that Erastine in the combination with 2DG and Cisplatin selective induces ferroptosis mediate cell death in CSCs isolated from SCCHN and may be used as a potential therapeutic strategies.

## Endocrine disruptor Bisphenol A enhances metastatic potential in androgen‐dependent LNCaP cells through epithelial to mesenchymal transition

**V. K. Singh^1,2^, M. I. Ansari^1,2^ & P. K. Sharma^1,2^**

*^1^Environmental Carcinogenesis Laboratory, Food Drug and Chemical Toxicology Group, CSIR‐Indian Institute of Toxicology Research, Lucknow, India; ^2^Academy of Scientific and Innovative Research, New Delhi, India*

*Email:* vipendra04@gmail.com

Endocrine disrupting chemicals such as Bisphenol A (BPA) have been shown to induce malignant transformation in androgen‐dependent tissues including prostate. However, the effect of BPA in prostate cancer metastasis remains poorly understood. Here, we have investigated the effect of BPA on metastatic potential in androgen‐dependent LNCaP cells exposed to environmentally relevant doses. Androgenic potential of BPA was confirmed through reporter cell line (MDA‐kb2) assay. Various parameters related to growth, proliferation, epithelial‐mesenchymal transition and invasion assays were studied in BPA treated LNCaP cells. Luciferase reporter assay demonstrated androgenic potential of BPA and enhanced androgen receptor (AR) expression in BPA treated LNCaP cells strongly suggested the androgenic potential of BPA. Both single and multiple exposure of BPA enhanced proliferation, colony formation ability and anchorage independent growth of LNCaP cells. Additionally, BPA exposure augmented migration and invasion potential of LNCaP cells. BPA also induced epithelial to mesenchymal transition (EMTs) in LNCaP cells as demonstrated by enhanced expression of various EMT markers via gene expression profiling. Furthermore, western blot analysis of metastatic and EMT markers confirmed the metastatic potential of BPA in AR positive LNCaP cells. Interestingly, BPA‐induced growth and metastatic changes were not recorded in AR negative prostate (PC‐3) cancer cells. In conclusion, our observations clearly suggest that environmental endocrine disruptors like BPA could regulate EMT and metastatic potential in AR‐positive prostate cancer cells that may have clinical relevance to androgen‐dependent prostate cancer.

## EZH2 mediated epigenetic repression of microRNA‐338‐5p/‐421 drives SPINK1‐positive prostate cancer

**V. Bhatia^1^, A. Yadav^1^, R. Tiwari^1^, S. Nigam^1^, S. Goel^1^, A. Goel^2^ & B. Ateeq^1^**

*^1^Molecular Oncology Lab, Department of Biological Sciences and Bioengineering, Indian Institute of Technology, Kanpur, Uttar Pradesh, India; ^2^Department of Urology, King George's Medical University, Lucknow, Uttar Pradesh, India*

*Email:* vipul@iitk.ac.in

Prostate cancer (PCa) is a clinically heterogeneous disease marked by variability in patient prognosis. The intra‐tumoral and inter‐patient heterogeneity formed the basis of molecular stratification of the disease. Overexpression of Serine Peptidase Inhibitor, Kazal type‐1 (*SPINK1*) was found to be the second most recurrent (˜10%–15%) PCa subtype, after highly recurrent (˜50%) androgen‐driven *TMPRSS2‐ERG* genetic rearrangement. Unlike amplification of *ERBB2* in breast cancer, SPINK1 overexpression has not been associated with gene amplification or rearrangement. Nonetheless, molecular mechanism underlying its upregulation in cancer is poorly understood and remains a matter of conjecture. Using *in‐silico* microRNA prediction tools, we shortlisted three miRNAs, of which miR‐338‐5p and miR‐421 revealed an inverse correlation with *SPINK1* expression in The Cancer Genome Atlas (TCGA) prostate adenocarcinoma RNA‐Seq dataset, which was validated in our PCa specimens. Here, we showed that miR‐338‐5p and miR‐421 post‐transcriptionally regulate *SPINK1* by binding to its 3′UTR. We established that miR‐338‐5p and miR‐421 mediate several cellular responses against *SPINK1*‐positive cancer by inducing S‐phase cell‐cycle arrest, inhibiting epithelial‐to‐mesenchymal transition (EMT), cancer stemness and drug resistance. Moreover, ectopic expression of miR‐338‐5p and miR‐421 abrogates SPINK1‐mediated oncogenesis, tumor growth and distant metastases in murine xenograft model. Further, mechanistically we demonstrate that Polycomb group protein Enhancer of zeste homolog 2 (EZH2) mediates epigenetic silencing by establishing histone H3K27me3 repressive marks and DNA methylation, 5′methyl cytosine (5mC) marks on the promoters of miR‐338‐5p and miR‐421 in SPINK1‐positive subtype. Thus, restoring miR‐338‐5p and miR‐421 expression using either epigenetic drugs or synthetic mimics could abrogate SPINK1‐mediated oncogenesis by targeting multiple oncogenic pathways and eliciting anti‐cancer pleiotropic effects. Taken together, the present study for the first time unravels the molecular mechanism underlying SPINK1 overexpression and thereby opens up new avenues using miR‐338‐5p and miR‐421 replacement therapy for the treatment of SPINK1‐positive malignancies.

## Ellagic acid helps combat Cisplatin induced hepatotoxicity in murine colon carcinogenesis

**Y. Goyal, A. Koul & P. Ranawat**

*Department of Biophysics, Punjab University, Chandigarh, Punjab, India*

*Email:* goyalyasmeen@gmail.com

The clinical use of Cisplatin (CP), one of the most widely used chemotherapeutic drug, is limited by its associated side effects such as neurotoxicity, hepatotoxicity and nephrotoxicity among others. The mechanism of action of CP involves excessive generation of reactive oxygen species (ROS) culminating in oxidative stress, which is a cause of the side effects associated with its use. Ellagic acid (EA), a polyphenol is known to possess multiple health benefits owing to its antioxidant properties. The present study was therefore, carried out to explore the protective potential of EA on CP induced hepatotoxicity in colon tumor bearing mice. The administration of EA (10 mg/kg b.wt. p.o daily for 6 weeks) significantly ameliorated the toxicity caused by CP (5 mg/kg b.wt. i.p once a week for 4 weeks). Activities of liver marker enzymes and lactate dehydrogenase were brought back to normal. EA co‐treatment also led to a marked reduction in the extent of peroxidative damage to liver tissue as was evident from the improvement in the histopathological changes observed and FT‐IR analysis. The present study therefore suggests that the administration of EA reduces the CP‐induced hepatotoxicity, thereby emerging out as a potential candidate for chemopreventive action.

## Effect of EPA and DHA (Omega‐3 fatty acids) on the growth and apoptosis of human lung adenocarcinoma cells *in vitro*

**F. Jameel, A. Jafri, M. Serajuddin & M. Arshad**

*Department of Zoology, University of Lucknow, Lucknow, Uttar Pradesh, India*

*E‐mail:* farheenj60@gmail.com

Eicosapentaenoic acid (EPA) and docosahexaenoic acid (DHA) are the two major types of Omega‐3 polyunsaturated fatty acids (PUFAs), known to occur in fish, have been reported an inverse correlation with the development of cancer. The *in vitro* and *in vivo* studies have shown that PUFA inhibit the growth of various kinds of cancers including lung cancer through inhibition of proliferation and leading to apoptosis in cancer cells. Preliminary study was done to investigate the effects of omega‐3 PUFA *i.e.,* EPA (20:5 *n*−3) and DHA (22:6 *n*−3) on the proliferation and apoptosis of human lung adenocarcinoma A549 cells. The cells were exposed to increasing concentrations (40–180 µM) of EPA and DHA for 24 h. Cell viability was assayed by MTT (3‐(4,5‐dimethylthiazolyl‐2)‐2,5‐diphenyltetrazolium bromide) and morphological studies. The apoptotic effect was analysed by DAPI dye for detection of nuclear condensation and reactive oxygen species (ROS) were monitored using the 2′,7′‐dichlorofluorescin diacetate (DCFDA) staining using inverted fluorescence microscope. Our findings revealed that both EPA and DHA significantly inhibits the cell growth and induces apoptosis in A549 cells, however the effect of DHA was more pronounced. MTT assay showed a progressive loss of cell viability in a dose dependent manner, fluorescence imaging of condensed and fragmented nuclei by DAPI dye indicates cell death by an apoptotic process. The excessive ROS generation was also observed at the higher doses. The two well‐known Omega‐3 fatty acids showed an anti‐proliferative effect on non‐small lung cancer, A549 cells *via* decrease in cell viability, increasing number of fragmented and condensed nuclei and increase in lipid peroxidation. Our result suggests that EPA and DHA have significant apoptotic potential on A549 cells. However, further studies are needed to find out its efficacy as a chemo‐preventive agent.

## Chronic exposure of low dose Bisphenol A enhances proliferating potential of breast cancer cells via genomic instability

**M. I. Ansari^1,2^, V. K. Singh^1,2^ & P. K. Sharma^1,2^**

*^1^Environmental Carcinogenesis Laboratory, Food Drug and Chemical Toxicology Group, CSIR‐Indian Institute of Toxicology Research, Lucknow, India; ^2^Academy of Scientific and Innovative Research, New Delhi, India*

*Email:* imranansarilko@gmail.com

Humans are routinely exposed to environmental relevant dose of Bisphenol A (BPA). BPA is a high volume produced plasticizer used in the production of food and beverage container worldwide. Although a number of studies have investigated the adverse effects of high dose as well as acute exposure of BPA. However, there has been a lack of mechanistic study of chronic effect of low dose BPA. In the present study we have investigated that chronic exposure (200 days) of BPA enhanced cellular proliferation and migration potential in human breast cancer cells. Various parameters such as MTT assay, growth kinetics for proliferation and soft agar assay for anchorage independent growth etc were performed. As compared to acute exposure, the chronic exposure of BPA significantly enhanced the proliferation as well as mitochondrial biogenesis in ER‐dependent MCF‐7 breast cancer cells. Moreover, the chronic exposure of BPA enhanced micronuclei formation (MN) and DNA double stranded breaks (DSBs) in hormone dependent breast cancer cells. Furthermore, these genomic alterations enhanced proliferation rate and anchorage independent growth in MCF‐7 cells. Here, we concluded that chronic exposure of low dose BPA significantly promote cancer proliferation and progression via genomic alterations. However, the mechanism underlying such BPA‐mediated altered growth and proliferation in ER‐dependent breast cancer cells needs more critical evaluation.

## Tumur promoting potential of Bisphenol A in 2‐stage mouse skin model of carcinogenesis

**I. Singh & Y. Shukla**

Environmental Carcinogenesis Lab Food, Drug & Chemical Toxicology Group, CSIR‐ Indian Institute of Toxicology Research, Lucknow, Uttar Pradesh, India

Bisphenol A (BPA,4,4′‐(propane‐2‐2‐diyl) diphenol) is used widely in making plastics, epoxy resins and thermal papers, but it is also a potential xenoestrogen, having endocrine disruptive property reported in the incidence of cancers. The aim of the present study was to evaluate carcinogenic effects of BPA on mouse skin using 2‐ stage mouse skin carcinogenesis model. The onset of tumorigenesis was recorded in the animals of positive control group II i.e. DMBA + TPA after 52 days of promotion. However, tumor development started in the animals of group III (DMBA + BPA (low dose)) after 84 days of promotion and in the animals of group IV (DMBA + BPA (high dose)) after 64 days of promotion. At the time of termination of experiment i.e. 32 weeks, 100% of the animals developed tumors on the dorsal region of the skin in positive control group and 30% in group III and 60% in IV, respectively. The total number of tumors in group II was 78 while in group III was 10 and IV was 21. No tumor development was observed in the animals of groups I (untreated control) during the entire period of study. Similarly, the average number of tumors/mouse was 6.1 ± 1.4 in group II; however, in group III and group IV, it was 1.3 ± 2.0 and 2.6 ± 2.9, respectively. These tumors were initiated as a minute wart like growth, which progressed during the course of experiment and average tumor volume was 89.4 ± 5.1 mm^3^ in group II and 19.57 ± 5.5 mm^3^ in group III and 55.71 ± 4.1 mm^3^ in group IV. These results clearly indicate significant tumor promoting potential of BPA in mouse skin model of carcinogenesis. Further studies are warranted to validate the carcinogenic potential of BPA using additional relevant skin cancer and *in vitro* models.

